# Supplementary material for: Experimental and theoretical investigation on the conductivity of complex fracture network in unconventional gas reservoirs
Source: PLoS One. 2025 Jun 13;20(6):e0325825. doi: 10.1371/journal.pone.0325825 (PMC12165408; doi:10.1371/journal.pone.0325825)
Supplement: Attachment — Submitted filename: PONE-D-25-07188_R1.pdf [file pone.0325825.s003.pdf]

PLOS ONE

Experimental and theoretical investigation on the conductivity of complex fracture network in unconventional gas reservoirs

--Manuscript Draft--

Manuscript Number: PONE-D-25-07188R1

Article Type: Research Article

Full Title: Experimental and theoretical investigation on the conductivity of complex fracture network in unconventional gas reservoirs

Short Title: Experimental and theoretical investigation on the conductivity of complex fracture network

Corresponding Author: jinjian gao
jiangxi flight university
Nanchang, CHINA

Keywords: Complex Fracture Network; New Conductivity Chamber; Fracture Conductivity; Secondary Fracture; Closure Pressure; New Model of Fracture Network Conductivity

Abstract: With the depletion of conventional oil and gas resources, unconventional oil and gas resources have become crucial as alternative hydrocarbon sources. Unconventional gas reservoirs typically have low porosity and permeability, requiring reservoir stimulation to create complex fracture networks. Unlike single fractures, the fracture networks exhibit complicated structures due to multiple fractures and their various distribution manners. To study the conductivity of fracture networks, a new conductivity chamber is designed based on a discretized fracture network model. Experiments and orthogonal analysis are conducted to investigate the effects of critical influential factors such as fracture network structure, fracture height, proppant concentration, proppant particle size, and proppant type on the conductivity of the fracture network. The results show that compared to a single fracture, the T-shape, F-shape, E-shape, ╪-shape, ł –shape fracture network increases conductivity by 49%, 131%, 150%, 188%, and 201% respectively. Closure pressure and secondary fracture distribution significantly impact the conductivity of the fracture network. Proppant particle size, the number of secondary fractures, and proppant concentration also have substantial effects. Based on the Kozeny-Carman equation and the similarity principle of water and electricity, a new model of fracture network conductivity is established. The advantage of new model of fracture network conductivity is that it combines a theoretical method and an experimental method. This study provides valuable insights for optimizing volume fracturing in tight reservoirs, facilitating their rapid and efficient development.With the depletion of conventional oil and gas resources, unconventional oil and gas resources have become crucial as alternative hydrocarbon sources. Unconventional gas reservoirs typically have low porosity and permeability, requiring reservoir stimulation to create complex fracture network. Unlike single fracture, the fracture network exhibit complicated structures due to multiple fractures and their various distribution manners. To study the conductivity of complex fracture network, a new conductivity chamber is designed based on a discretized fracture network model. Experiments and orthogonal analysis are conducted to investigate the effect of critical influential factors such as fracture network structure, fracture height, proppant concentration, proppant diameter, and proppant type on the conductivity of complex fracture network. The results show that compared to a single fracture, the T-shape, F-shape, E-shape, ╪-shape, ł –shape fracture networks conductivity increase by 49%, 131%, 150%, 188%, 201%, respectively. Closure pressure and secondary fracture distribution significantly impact the conductivity of complex fracture network. Proppant diameter and proppant concentration also have substantial effects. Compared with existing experimental studies focusing on single fracture conductivity, this study presents experimental research on the conductivity of complex fracture network, thereby addressing a research gap in this field. Through synergistic integration of the Kozeny-Carman hydrodynamic theory with hydraulic-electrical analogy principle, an innovative model of fracture network conductivity is formulated. The advantage of new model of fracture network conductivity is that it combines a theoretical method and an experimental method. This study provides valuable insights for optimizing volume fracturing in tight reservoirs, facilitating their rapid and efficient development.

Powered by Editorial Manager® and ProduXion Manager® from Aries Systems Corporation

**Order of Authors:**	jinjian gao
	Lanxiao hu
**Opposed Reviewers:**	qingzhi wen<br>Peking University<br><br>Professor Wen has long been engaged in reservoir modification research, mainly focusing on hydraulic fracturing, and has profound academic achievements
	fankun meng<br>Yangtze University<br><br>Professor Wen has long been engaged in reservoir modification research, mainly focusing on hydraulic fracturing, and has profound academic achievements
**Response to Reviewers:**	First and foremost, I would like to express my sincere gratitude for the thorough review and insightful comments provided on our manuscript titled " Experimental and theoretical investigation on the conductivity of complex fracture network in unconventional gas reservoirs ". Those comments are all valuable and very helpful for revising and improving our paper, as well as the important guiding significance to our researches. We have carefully considered each point raised and have made the following revisions to our manuscript.
**Additional Information:**	
**Question**	**Response**
**Financial Disclosure**<br><br>Enter a financial disclosure statement that describes the sources of funding for the work included in this submission. Review the submission guidelines for detailed requirements. View published research articles from *PLOS ONE* for specific examples.<br><br>This statement is required for submission and **will appear in the published article** if the submission is accepted. Please make sure it is accurate.<br><br>**Funded studies**<br>Enter a statement with the following details:<br>• Initials of the authors who received each award<br>• Grant numbers awarded to each author<br>• The full name of each funder<br>• URL of each funder website<br>• Did the sponsors or funders play any role in the study design, data collection and analysis, decision to publish, or preparation of the manuscript?<br><br>Did you receive funding for this work?	Yes

Please add funding details.<br>as follow-up to "**Financial Disclosure**<br><br>Enter a financial disclosure statement that describes the sources of funding for the work included in this submission. Review the submission guidelines for detailed requirements. View published research articles from *PLOS ONE* for specific examples.<br><br>This statement is required for submission and **will appear in the published article** if the submission is accepted. Please make sure it is accurate.<br><br>**Funded studies**<br>Enter a statement with the following details:<br>• Initials of the authors who received each award<br>• Grant numbers awarded to each author<br>• The full name of each funder<br>• URL of each funder website<br>• Did the sponsors or funders play any role in the study design, data collection and analysis, decision to publish, or preparation of the manuscript?<br><br>Did you receive funding for this work?"	the Natural Science Foundation of Sichuan Province (2023NSFSC0945)
Please select the country of your main research funder (please select carefully as in some cases this is used in fee calculation).<br>as follow-up to "**Financial Disclosure**<br><br>Enter a financial disclosure statement that describes the sources of funding for the work included in this submission. Review the submission guidelines for detailed requirements. View published research articles from *PLOS ONE* for specific examples.<br><br>This statement is required for submission and **will appear in the published article** if the submission is accepted. Please make sure it is accurate.	CHINA - CN

Powered by Editorial Manager® and ProduXion Manager® from Aries Systems Corporation

**Funded studies**<br>Enter a statement with the following details:<br>• Initials of the authors who received each award<br>• Grant numbers awarded to each author<br>• The full name of each funder<br>• URL of each funder website<br>• Did the sponsors or funders play any role in the study design, data collection and analysis, decision to publish, or preparation of the manuscript?<br><br>Did you receive funding for this work?"	
**Competing Interests**<br><br>Use the instructions below to enter a competing interest statement for this submission. On behalf of all authors, disclose any competing interests that could be perceived to bias this work—acknowledging all financial support and any other relevant financial or non-financial competing interests.<br><br>This statement is required for submission and **will appear in the published article** if the submission is accepted. Please make sure it is accurate and that any funding sources listed in your Funding Information later in the submission form are also declared in your Financial Disclosure statement.<br><br>View published research articles from *PLOS ONE* for specific examples.	The authors have declared that no competing interests exist.

Powered by Editorial Manager® and ProduXion Manager® from Aries Systems Corporation

**NO authors have competing interests**<br><br>Enter: *The authors have declared that no competing interests exist.*<br><br>**Authors with competing interests**<br><br>Enter competing interest details beginning with this statement:<br><br>*I have read the journal's policy and the authors of this manuscript have the following competing interests: [insert competing interests here]*<br><br>* typeset	
**Ethics Statement**<br><br>Enter an ethics statement for this submission. This statement is required if the study involved:<br><br>• Human participants<br>• Human specimens or tissue<br>• Vertebrate animals or cephalopods<br>• Vertebrate embryos or tissues<br>• Field research<br><br>Write "N/A" if the submission does not require an ethics statement.<br><br>General guidance is provided below. Consult the submission guidelines for detailed instructions. **Make sure that all information entered here is included in the Methods section of the manuscript.**	N/A

Powered by Editorial Manager® and ProduXion Manager® from Aries Systems Corporation

**Format for specific study types**<br><br>**Human Subject Research (involving human participants and/or tissue)**<br>• Give the name of the institutional review board or ethics committee that approved the study<br>• Include the approval number and/or a statement indicating approval of this research<br>• Indicate the form of consent obtained (written/oral) or the reason that consent was not obtained (e.g. the data were analyzed anonymously)<br><br>**Animal Research (involving vertebrate animals, embryos or tissues)**<br>• Provide the name of the Institutional Animal Care and Use Committee (IACUC) or other relevant ethics board that reviewed the study protocol, and indicate whether they approved this research or granted a formal waiver of ethical approval<br>• Include an approval number if one was obtained<br>• If the study involved *non-human primates*, add *additional details* about animal welfare and steps taken to ameliorate suffering<br>• If anesthesia, euthanasia, or any kind of animal sacrifice is part of the study, include briefly which substances and/or methods were applied<br><br>**Field Research**<br><br>Include the following details if this study involves the collection of plant, animal, or other materials from a natural setting:<br>• Field permit number<br>• Name of the institution or relevant body that granted permission	
**Data Availability**<br><br>Authors are required to make all data underlying the findings described fully available, without restriction, and from the time of publication. PLOS allows rare exceptions to address legal and ethical concerns. See the PLOS Data Policy and FAQ for detailed information.	Yes - all data are fully available without restriction

A Data Availability Statement describing where the data can be found is required at submission. Your answers to this question constitute the Data Availability Statement and **will be published in the article**, if accepted.<br><br>**Important:** Stating 'data available on request from the author' is not sufficient. If your data are only available upon request, select 'No' for the first question and explain your exceptional situation in the text box.<br><br>Do the authors confirm that all data underlying the findings described in their manuscript are fully available without restriction?	
**Describe where the data may be found in full sentences. If you are copying our sample text, replace any instances of XXX with the appropriate details.**<br><br>• If the data are **held or will be held in a public repository**, include URLs, accession numbers or DOIs. If this information will only be available after acceptance, indicate this by ticking the box below. For example: *All XXX files are available from the XXX database (accession number(s) XXX, XXX.).*<br>• If the data are all contained **within the manuscript and/or Supporting Information files**, enter the following: *All relevant data are within the manuscript and its Supporting Information files.*<br>• If neither of these applies but you are able to provide **details of access elsewhere**, with or without limitations, please do so. For example:<br><br>*Data cannot be shared publicly because of [XXX]. Data are available from the XXX Institutional Data Access / Ethics Committee (contact via XXX) for researchers who meet the criteria for access to confidential data.*<br><br>*The data underlying the results presented in the study are available from (include the name of the third party*	All relevant data are within the manuscript and its Supporting Information files.

and contact information or URL).<br>• This text is appropriate if the data are owned by a third party and authors do not have permission to share the data.<br><br>* typeset	
Additional data availability information:	

Dear Editors,

We are pleased to submit our manuscript titled “Experimental and theoretical investigation on the conductivity of complex fracture network in unconventional gas reservoirs” for consideration in PLOS ONE. This study investigates the conductivity of fracture networks. A new conductivity chamber is designed based on a discretized fracture network model. Experiments and orthogonal analysis are conducted to investigate the effects of critical influential factors. A new model of fracture network conductivity is established. The advantage of new model of fracture network conductivity is that it combines a theoretical method and an experimental method. We believe our findings will be of broad interest to the scientific community, as they provide valuable insights for optimizing volume fracturing in tight reservoirs, facilitating their rapid and efficient development..

We confirm that this manuscript is original, has not been published elsewhere, and is not under consideration by any other journal. All authors have approved the manuscript and its submission to *PLOS ONE*. We have no conflicts of interest to disclose.

We would like to suggest the following potential reviewers for our manuscript:

1,Fankun Meng, Yangtze University, mengfk@yangtzeu.edu.cn

2, Zehua Cheng, China University of Petroleum East China, chenzehua1210@163.com

Alternatively, we are happy to rely on the expertise of the editorial board to select appropriate reviewers.

Thank you for considering our submission. We look forward to the possibility of contributing to PLOS ONE. Please do not hesitate to contact us if you require any additional information or materials.

Sincerely,

Jinjian Gao

Click here to access/download;Manuscript;manuscript 20250210.docx

Experimental and theoretical investigation on the conductivity of complex fracture network in unconventional gas reservoirs

Jinjian Gao[1*], Lanxiao Hu[2]

1 Airport department, Jiangxi Flight University, Nanchang, 330088, Jiangxi, China

2 College of Energy Resources, Chengdu University of Technology, Chengdu, 610059, Sichuan, China

*Corresponding author, gaojinjian1990@163.com

These authors contributed equally to this work.

Abstract

With the depletion of conventional oil and gas resources, unconventional oil and gas resources have become crucial as alternative hydrocarbon sources. Unconventional gas reservoirs typically have low porosity and permeability, requiring reservoir stimulation to create complex fracture networks. Unlike single fractures, the fracture networks exhibit complicated structures due to multiple fractures and their various distribution manners. To study the conductivity of fracture networks, a new conductivity chamber is designed based on a discretized fracture network model. Experiments and orthogonal analysis are conducted to investigate the effects of critical influential factors such as fracture network structure, fracture height, proppant concentration, proppant particle size, and proppant type on the conductivity of the fracture network. The results show that compared to a single fracture, the T-shape, F-shape, E-shape, ╪-shape, Ɨ –shape fracture network increases conductivity by 49%, 131%, 150%, 188%, and 201% respectively. Closure pressure and secondary fracture distribution significantly impact the conductivity of the fracture network. Proppant particle size, the number of secondary fractures, and proppant concentration also have substantial effects. Based on the Kozeny-Carman equation and the similarity principle of water and electricity, a new model of fracture network conductivity is established. The advantage of new model of fracture network conductivity is that it combines a theoretical method and an experimental method. This study provides valuable insights

for optimizing volume fracturing in tight reservoirs, facilitating their rapid and efficient development.

Keywords: Complex Fracture Network; New Conductivity Chamber; Fracture Conductivity; Secondary Fracture; Closure Pressure; New Model of Fracture Network Conductivity

1.Introduction

Unconventional gas reservoirs generally have no natural productivity without fracturing. Hydraulic fracturing has become one of the pivot technologies in the development of tight gas reservoirs, enabling them to achieve industrial gas flows and making the development of tight gas reservoirs feasible [1-5]. Typically, the fractures formed in conventional reservoirs are single, symmetrical, bi-wing fractures. However, unconventional gas reservoirs have many natural fractures and possess high brittleness [6-8], which tends to create complex fracture networks rather than the single fracture; these observations have been extensively found in the North American hydraulic fracturing practices [9-13]. During the fracturing process, brittle rocks and natural fractures continuously open or shear, forming a complex network of artificial and natural fractures. This results in a significant increase in stimulated reservoir volume [14-17]. The technique to enhance production and ultimate recovery by creating such complex fracture networks is known as volume fracturing, which plays an increasingly important role in the development of unconventional oil and gas reservoirs.

As ~~is~~ well known, fracture conductivity is a key parameter for volume fracturing. In recent years, experiments of fracture conductivity have evolved from single proppant types and sizes to multiple types and mixed sizes. Wen et al. [18] pioneered the use of the long-term fracture conductivity instrument in China to study proppant embedding by using formation cores. However, research object was single fracture. Lu et al. [19] simulated fracture networks using various grooves with rectangular cross-sections. They examined the conductivity of five different network structures: "一", "T", "L", "F", and "╪". It proves that the fracture network created by volume fracturing have different influence on fracture conductivity. This experiment ignored

crushing and embedment of proppant. Wen et al. [20] designed a complex fracture network simulation device to study proppant settlement in "一", "T", "H", and "TF" network configurations based on discretized fracture network model. This provides a foundation for the experiment in this article. Liang et al. [21] evaluated the fracture conductivity with various ratios of sands to ceramic proppants by use of standard Fracture Conductivity Evaluation System under different effective closure stresses. Wang et al. [22] conducted triaxial experiments to explore the effects of displacement, stress difference, and weak surfaces. Shi et al. [23] examined the fracture conductivity of tight dolomite reservoirs, analyzing various influencing factors. They found that proppant diameter and sand concentration significantly impacted fracture conductivity. Katende et al. [24] employed experimental and analytical simulation methods to study proppant embedment and fracture conductivity within Caney Shale fractures. Improved fracture conductivity is attributed to proppant size. Liu et al. [25] conducted experiments on long-term non-uniform sand spreading in hydraulic fractures of tight reservoirs, considering different influencing factors. Based on the conductivity loss mechanism, the calculation model of the long-term conductivity of heterogeneous artificial fractures is established.

In order to guide hydraulic fracturing field, the single hydraulic fracture conductivity model has been established. There are two types of models, one is an empirical model based on experimental results, and the other is a theoretical model derived from fluid mechanics in porous medium and capillary model. Li et al. [26] created a new mathematical model to calculate fracture conductivity and proppant embedment. Moreover, factors affecting the fracture conductivity have been analyzed including proppant diameter, closure pressure, and proppant type. Liu et al. [27] proposed an innovative fracture conductivity calculation model based on a "fracturing efficiency index". Liu et al. [28] established a new model for predicting the fracture conductivity considering the particle size redistribution caused by proppant crushing based on Kozeny–Carman equation. However, the most important point has been overlooked: the fracture network is composed of multiple fractures.

In previous research, experimental research on fracture conductivity has mainly focused on single fracture. The experimental instrument for fracture conductivity is still traditional diversion chamber which cannot meet the requirements of testing the conductivity of complex fracture network. There is very little research on the calculation model of the conductivity of fracture network.

Firstly, a new diversion chamber was designed and manufactured based on a discrete fracture network model. Then, rock slab is combined into fracture network. Experiments with different fracture structures will be conducted using a new type of diversion chamber. Orthogonal experiments were used to study the primary and secondary factors affecting the conductivity of fracture network. Finally, Based on the Kozeny-Carman equation and the similarity principle of water and electricity, a new model of fracture network conductivity is established.

2 Experimental methods

Traditional conductivity measurement chambers can only test the conductivity of a single fracture, falling short of testing complex fracture network conductivity. In this study, we propose a new conductivity measurement chamber based on the experimental observations obtained from the settlement and transportation of proppant in the complex fracture networks [20] and discrete fracture networks [29, 30]. This new chamber features a larger inner cavity space enabling the investigation of fracture networks and can be integrated with the fracture conductivity measurement setup.

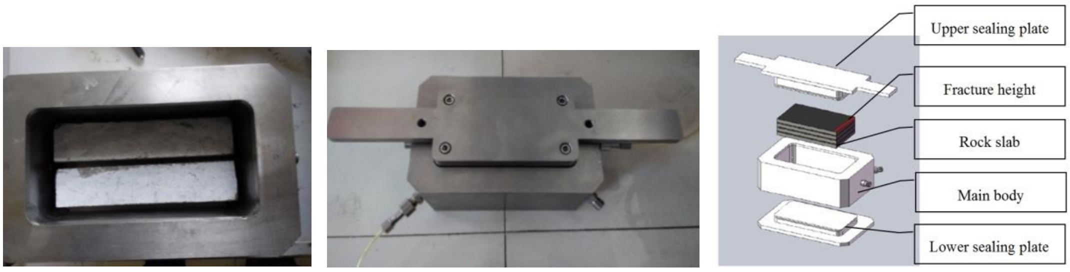

（a） **Internal diagram** （b） **External image** （c） **Explosion view**

Figure1. Structure diagram of new conductivity measurement chamber

Figure 1 illustrates the internal structure of the new conductivity measurement chamber with a dimension of 170mm×90mm×50mm. The chamber is equipped with stainless steel pads to adjust the fracture height. Various shapes of rock slabs simulate

different fracture networks, the surrounding space is sealed with foam rubber. There are four interfaces to monitor the inlet and outlet pressures and flow rates. Compared with the standard conductivity measurement cell, the new proposed chamber possesses the following features:

(1) The diversion chamber, constructed from stainless steel, undergoes wire cutting internally to ensure smooth cavity walls.

(2) The rock slab is positioned 10mm from the edge wall on one side. This arrangement ensures even gas distribution at the fracture's inlet and outlet, mitigating the end-effect and enhancing test accuracy.

(3) Due to the application of sealant on both sides of the rock slab, pressure measurement on the walls is not feasible. Pressure measuring ports are located on the inlet and outlet side walls to obtain the pressure differences. The effective fracture length is 160mm.

(4) Filter plugs are installed in the gas inlet and pressure measuring ports to prevent the leakage of the crushed proppant during the experimental process.

(5) The maximum closure pressure is 100 MPa.

The experimental instrument primarily comprises six components, as illustrated in **Figure 2**.

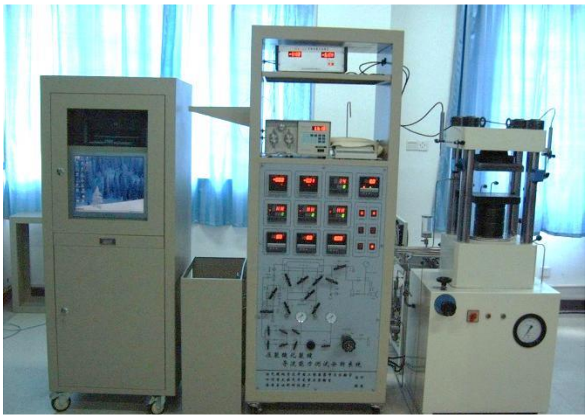

Figure 2. Fracture conductivity instrument of FCES-100

This experiment uses the volume equivalence principle, where the void volume in the fracture network equates to an imaginary single fracture's volume. The fracture network width is assumed to be the same as the width of the imaginary single fracture.

This width is calculated by dividing the total volume by the surface area parallel to the seepage direction:

$$w_f = V / S \tag{1}$$

Where w_f is equivalent fracture width (cm), V is proppant volume (cm^3), S is horizontal area of fracture network (cm^2).

Calculation equation for fracture conductivity can be written as:

$$kw_f = \frac{Q\mu L w_f}{A_f \Delta P} \tag{2}$$

Where k is permeability (μm^2), Q is flow rate(cm^3/s), μ is gas viscosity chamber(mPa·s), L is fracture conductivity length (cm), A_f is cross section area of fracture network (cm^2), ΔP is pressure difference between inlet and outlet(0.1MPa).

We construct several typical fracture network structures based on the discretized fracture network model. **Figure 3** summarizes these structures, including "─" type, "T" type, "F" type, "E" type, "+" type, "╪" type, and " Ɨ " type (Sun et al. 2024). For the conductivity measurements, it is assumed that the width of the secondary fracture is equal to that of the main fracture.

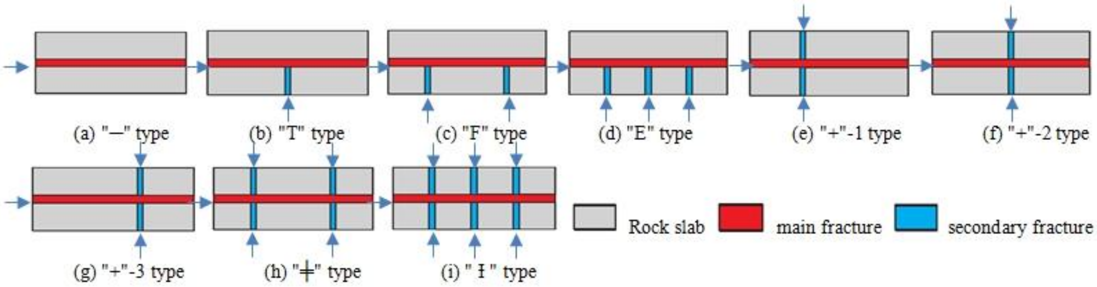

Figure 3. Schematics of different types of fracture networks

Taking the "─" type fracture network as an example, the experimental scheme for changing the sand concentration is as follows: the fracture height is 50mm, and the sand concentrations are 1.5, 2, 2.5, 3, 3.5, and 4 kg/m^2. The proppant used is 20-40 mesh ceramic proppant. The experimental scheme for changing the fracture height is as follows: the fracture heights are 23, 36, and 50 mm, with a sand concentration of 4 kg/m^2. The proppant used is 20-40 mesh ceramic proppant. The experimental scheme for changing the proppant diameter is as follows: the fracture height is 50 mm, the sand

concentration is 4 kg/m², and the proppants are 20-40, 40-70, and 80-100 mesh ceramic proppants. For the experimental scheme involving different types of proppants, only the proppant type is changed to ceramsite and quartz sand. The experimental scheme for other fracture structures is similar to that of the "—" type fracture.

Experimental procedure: Cut the rock slab and combine it into a fracture network. Place a specific amount of proppant in the fractures. Seal the gap and unpropped fracture with sealant, and install the sealing plate in the measurement chamber. Connect the inlet and outlet pressure and flow ports to the chamber. Pressurize the diversion chamber with closure pressures of 10MPa, 20MPa, 30MPa, 40MPa, 50MPa, and 60MPa.

3.Results and discussion

3.1 Experimental results of "—type" fracture network

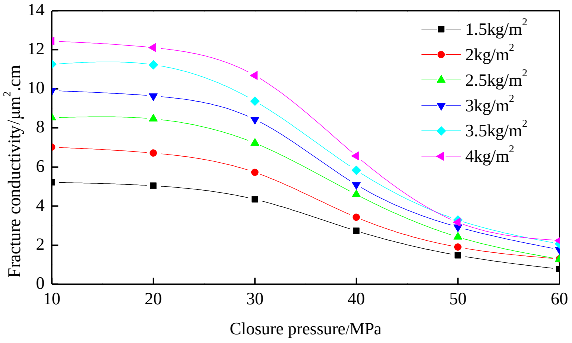

Figure 4. Fracture conductivity of "—" fracture network with different ceramic concentration

Figure 4 shows the conductivity of the fracture network under different closure pressures and sand concentrations. The trend of the fracture network's conductivity with closure pressure is similar across different sand concentrations; As closure pressure increases, the conductivity of the fracture network decreases. When the sand concentration is 4 kg/m², the conductivity is 12.446 μm²·cm at 10 MPa, decreases to 10.681 μm²·cm at 30 MPa, and drops to 2.228 μm²·cm at 60 MPa. Based on the analysis of six sets of curves, the decrease in conductivity under high closure pressure (60MPa) is between 81.7% and 85.1% compared to low closure pressures (10MPa). At low closure pressure, the proppant stays intact. The large pores formed by the proppant results in high permeability and greater fracture conductivity. The decrease in conductivity is due to the elastic deformation of the proppant, which slightly reduces

pore size. At high closure pressure, the proppant breaks. The broken ceramic proppant has low porosity, resulting in lower permeability and significantly reduced fracture conductivity.

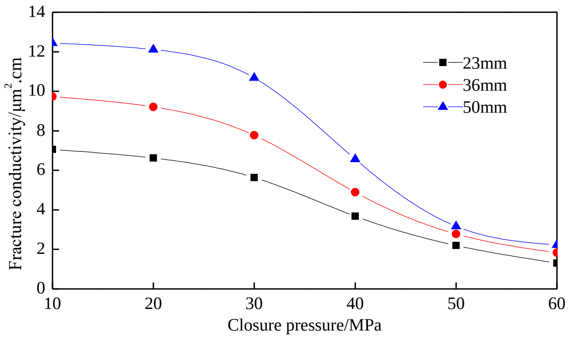

Figure 5. Fracture conductivity of "—" fracture network with different fracture height

From **Figure 5**, it can be observed that an increase in closure pressure leads to a decrease in fracture conductivity. The conductivity of the fracture network increases with fracture height. The fracture conductivity increases exponentially with fracture height. To increase fracture height in the deep part of the fracture, it's necessary to use high displacement and high viscosity fracturing fluid to carry the proppant, forming a high and long propped fracture to improve the conductivity.

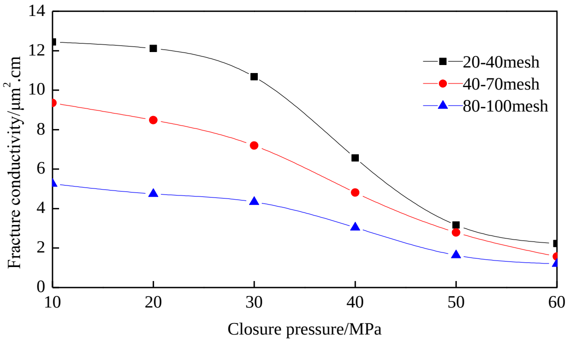

Figure 6. Fracture conductivity of "—" fracture network with different proppant size

Figure 6 shows that the conductivity of the fracture network increases with the proppant diameter. The 20~40 mesh proppant has the highest conductivity, while the 80~100 mesh proppant has the lowest due to its smaller particle diameter. The difference in conductivity among the three proppant diameters is significant under low-pressure conditions but less notable under high-pressure conditions. Due the complex structure and various fracture width of the fracture network, larger proppant cannot easily enter the small fractures. Based on the experimental results, the optimal

proppant size for volume fracturing is 40~70 mesh, which has a smaller decrease in conductivity compared to the 20-40 mesh proppant and can be carried into the narrows fractures.

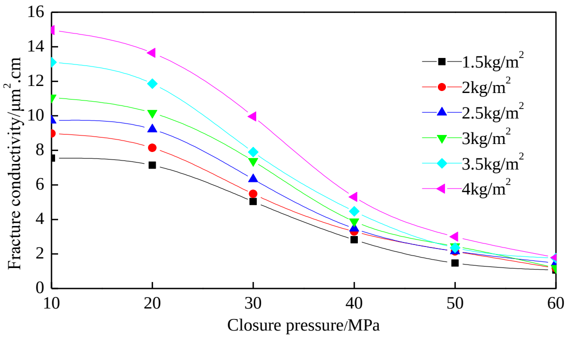

Figure 7. Fracture conductivity of "—" fracture network with different quartz sand concentration

Comparing **Figure4** and **Figure7**, the fracture conductivity of quartz sand at 10 MPa is slightly higher than that of ceramic proppant. However, under high pressure, it is lower than that of ceramic proppant. The fracture conductivity decreases by 92.1% with the closure pressure, indicating that the quartz sand possess neglible conductivity. It can be concluded that when the closure pressure is greater than 30 MPa, quartz sand cannot effectively provide higher conductivity and is unsuitable for high closure pressure conditions.

3.2.The influence of fracture network structure on conductivity

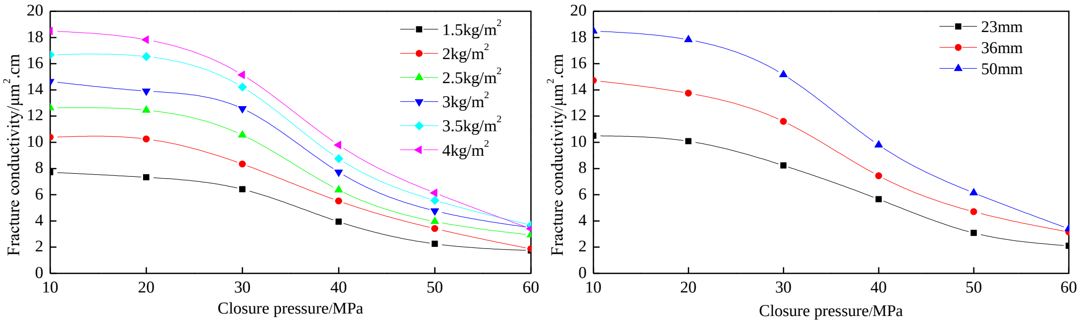

(a) Concentration of ceramic proppant **(b) Fracture height**

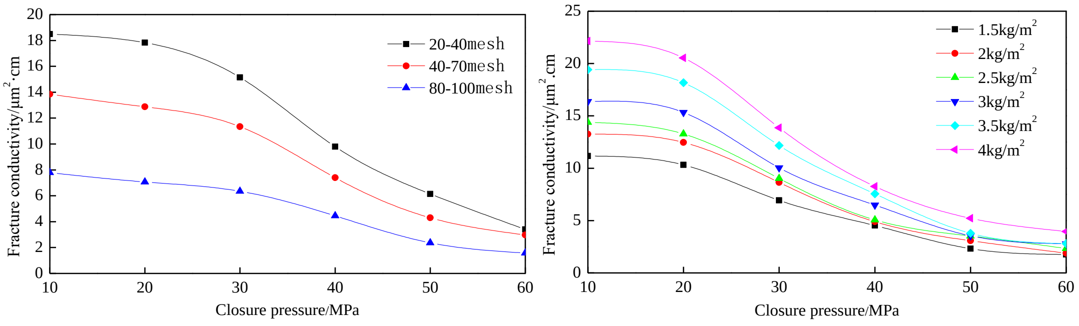

(c) Proppant size (d) Concentration of quartz sand

Figure 8. Experimental results of "T" fracture network

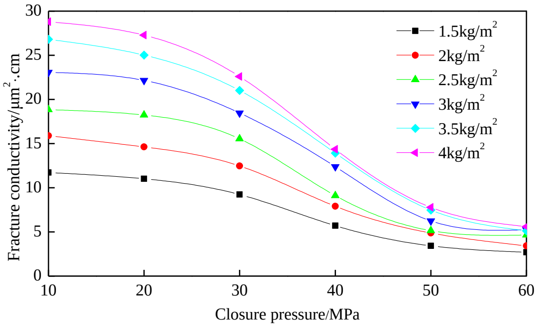

(a) Concentration of ceramic proppant

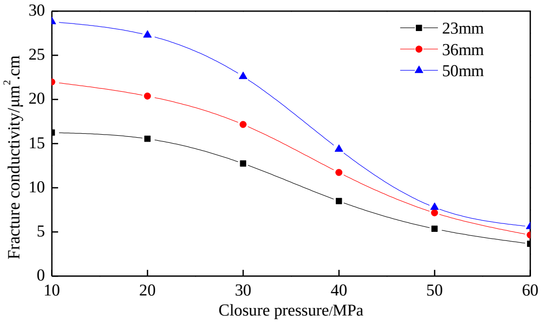

(b) Fracture height

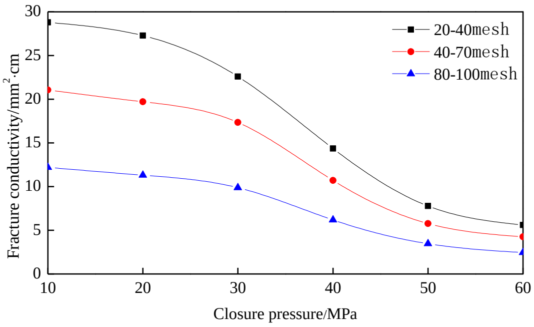

(c) Proppant size

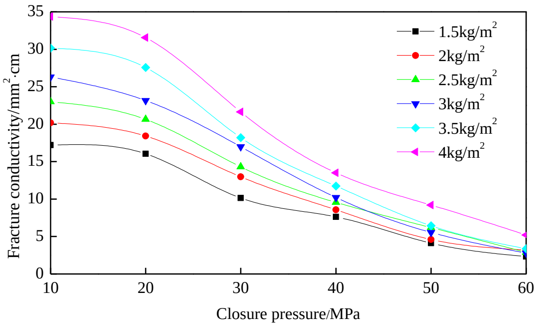

(d) Concentration of quartz sand

Figure 9. Experimental results of "F" fracture network

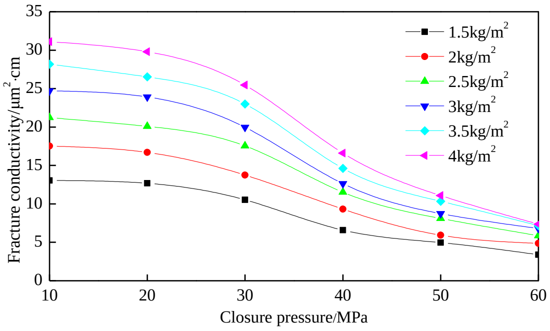

(a) Concentration of ceramic proppant

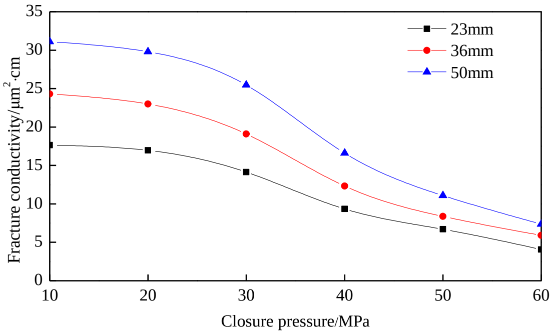

(b) Fracture height

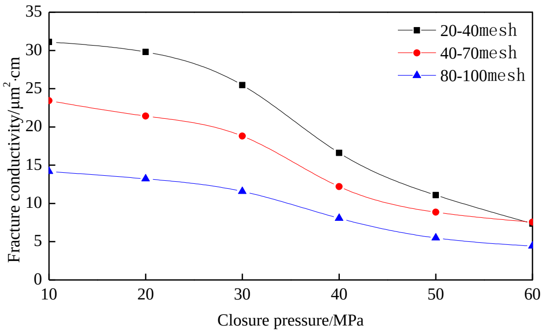

(c) Proppant size

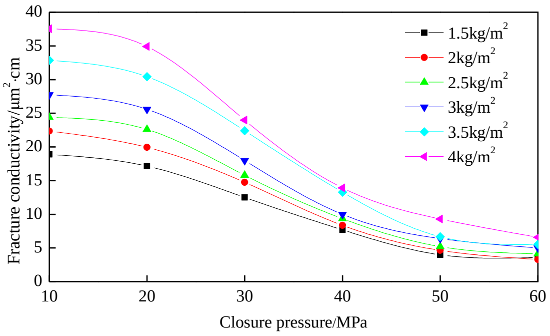

(d) Concentration of quartz sand

Figure 10. Experimental results of "E" fracture network

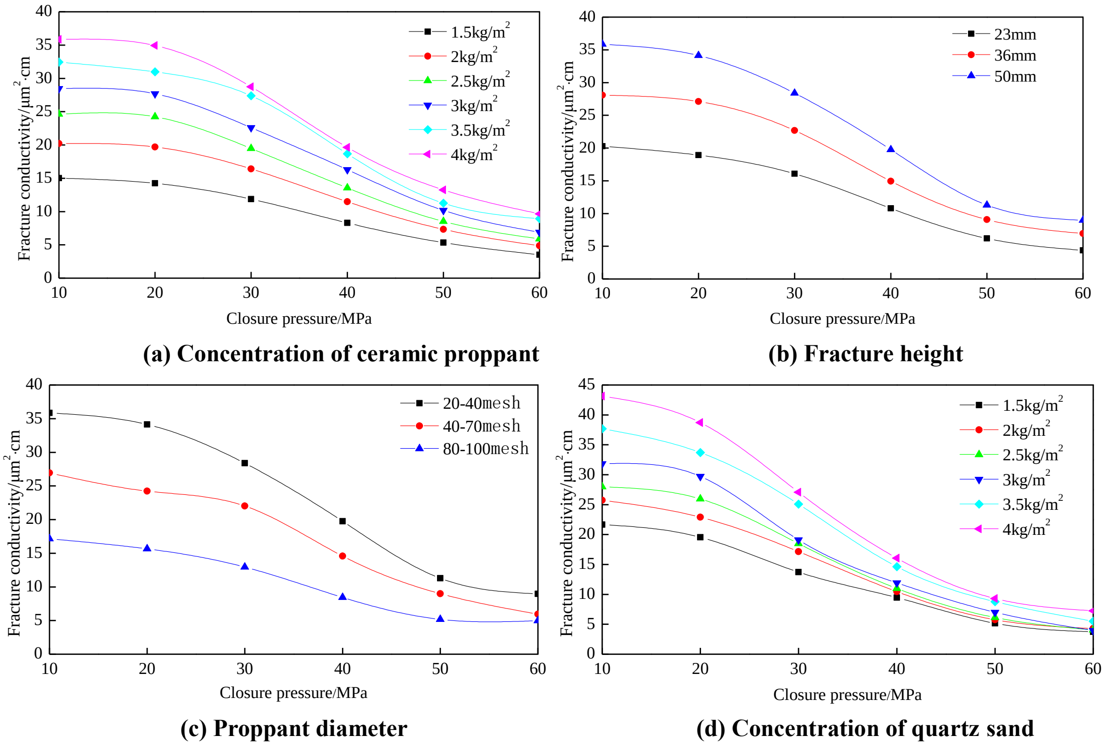

(a) Concentration of ceramic proppant **(b) Fracture height**

(c) Proppant diameter **(d) Concentration of quartz sand**

Figure 11. Experimental results of "╪" fracture network

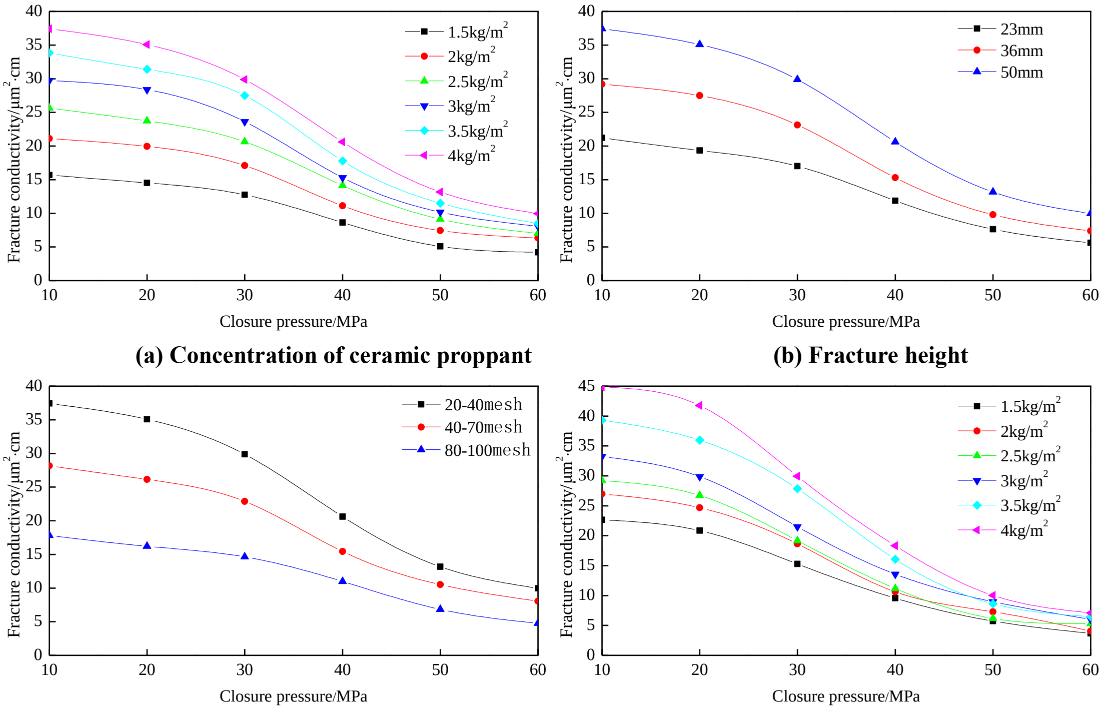

(a) Concentration of ceramic proppant **(b) Fracture height**

(c) Proppant diameter **(d) Concentration of quartz sand**

Figure 12. Experimental results of "Ɨ" fracture network

Figure 8~12 shows the conductivity of different fracture networks under different condition.

The conductivities of different types of fracture networks are analyzed under the same conditions.

Table1 Influence of secondary fracture number on fracture conductivity

Fracture network	"T" type	"F" type	"E" type	"╪" type	" Ɨ" type
Number of secondary fractures	1	2	3	4	6
Initial fracture conductivity growth factor	1.49	2.31	2.50	2.88	3.01
Final conductivity /Initial conductivity	18.4%	19.5%	23.7%	26.9%	29.3%

From **Table 1**, it can be seen that compared to the "─ type" fracture network, the initial fracture conductivity continuously increases with the number of secondary fractures. The growth factors of the fracture network conductivity are 1.49, 2.31, 2.50, 2.88, and 3.01 for "T", "F", "E", "╪", and "Ɨ" types, respectively. The ratio of the final conductivity (60MPa) to the initial conductivity (10MPa) also indicates that more secondary fractures lead to greater final fracture conductivity. Sufficient secondary fractures under high pressure can ensure that the formation maintains a certain degree of diversion capacity.

Table2 Influence of secondary fracture position on fracture conductivity

Fracture network	“+”-1 type	“+”-2 type	“+”-3 type
Distance from air outlet /mm	120	80	40
Initial fracture conductivity growth factor	1.20	1.66	2.82
Final conductivity /Initial conductivity	20.6%	22.1%	24.9%

From **Table 2**, it can be seen that the closer the secondary fractures are to the outlet of the chamber, the greater the increase in the conductivity of the fracture network. From the ratio of the final conductivity (60MPa) to the initial conductivity (10MPa), it can be seen that the closer the secondary fractures are to the outlet, the greater the conductivity of the fracture network under high pressure. The closer the secondary fracture is to the outlet, the smaller the resistance during the flow process and the lower the energy loss in the secondary fractures. When the fluid enters the main fracture, the closer it is to the outlet, the lower the energy of the fluid in the main fracture, which means that the energy loss of mixing the fluid in the main and secondary fractures is the smallest and the increase in flow capacity is the largest.

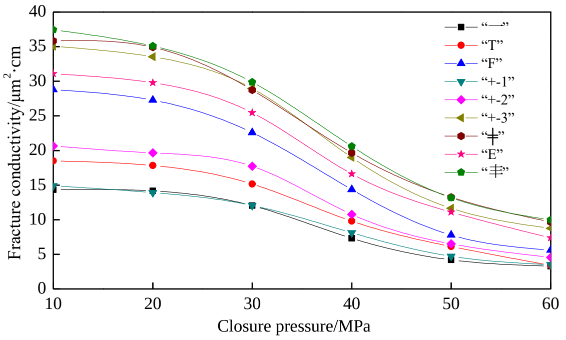

Figure 13. Comparison of the conductivities of ten types of fracture network

Using the 20~40 mesh ceramic proppant, with a sand concentration of 4 kg/m² and a fracture height of 50 mm, conductivity values obtained from nine types of fracture networks were plotted in **Figure 13**. The order of conductivity is as follows: "—" type < "+"-1 type < "T" type < "+"-2 type < "F" type < "E" type < "+"-3 type < "╪" type < "Ɨ" type. The following observations have been found: (1) The conductivities of the "—" and "+"-1 fracture networks are close to each other and exhibit lowest conductivity values. Although the "+"-1 fracture network has two secondary fractures, their locations are far away from the outlet, resulting in high pressure consumption during flow and significant energy loss when the fluid converges from the secondary fractures into the main fracture. (2) The conductivity of the "+"-2 fracture network is slightly higher than that of the "T" fracture network. The secondary fracture positions are the same, but the "+"-2 network has an additional secondary fracture. (3) The conductivity of the "F" and "E" fracture networks is similar and significantly improved. Both have one secondary fracture closest to the outlet, indicating that the position of the secondary fracture significantly impacts the conductivity. (4) The conductivity of the "+"-3, "╪", and "Ɨ" types of fracture networks is the highest because they have two secondary fractures closest to the outlet. The "Ɨ" type has 6 secondary fractures, the "╪" type has 4, and the "+"-3 type has 2. Therefore, the "Ɨ" type has the highest conductivity, followed by the "╪" type, and the "+"-3 type has the lowest among the top three.

3.3 Orthogonal experimental analysis

Results of the orthogonal experiments are showed in **Table 3.**

Table3 Results of the orthogonal experiments

Number	Proppant	Number of secondary fractures	Distance from air outlet /mm	Fracture height /mm	Sand concentration /(kg/m^2)	Proppant diameter /mesh	Closure pressure /MPa	Error	Conductivity /μm^2·cm
1	Ceramsite	1	120	23	2	20-40	20	1	4.623
2		1	80	36	3	40-70	40	2	5.027
3		1	40	50	4	80-100	60	3	2.994
4		2	120	23	3	40-70	60	3	1.215
5		2	80	36	4	80-100	20	1	6.948
6		2	40	50	2	20-40	40	2	10.936
7		4	120	36	2	80-100	40	3	2.073
8		4	80	50	3	20-40	60	1	6.875
9		4	40	23	4	40-70	20	2	15.804
10	Quartz sand	1	120	50	4	40-70	40	1	6.305
11		1	80	23	2	80-100	60	2	1.147
12		1	40	36	3	20-40	20	3	17.339
13		2	120	36	4	20-40	60	2	1.674
14		2	80	50	2	40-70	20	3	6.624
15		2	40	23	3	80-100	40	1	3.402
16		4	120	50	3	80-100	20	2	6.378
17		4	80	23	4	20-40	40	3	8.627
18		4	40	36	2	40-70	60	1	2.774

According to the verification results in **Table 4**, the closure pressure and the position of secondary fractures have the greatest impact on the conductivity of the fracture network. The proppant diameter, the number of secondary fractures, and the sand concentration also significantly affect conductivity, while the type of proppant and the fracture height have a weaker impact.

Table4 Analyses of the variances

Source of variance	Sum of squares	free degree	Mean sum of squares	F value	Significance
Proppant	0.275	1	0.275	0.088	
Number of secondary fractures	11.536	2	5.768	1.851	Saliency
Distance from air outlet	80.685	2	40.343	12.945	High saliency
Fracture height	2.631	2	1.315	0.422	
Sand concentration	19.490	2	9.745	3.127	Saliency
Proppant diameter	61.517	2	30.758	9.870	Saliency
Closure pressure	140.412	2	70.206	22.527	High saliency
Error	9.349	3	3.116		

Under high closure pressure, the proppant is crushed, leading to a significant reduction in fracture width and pore size, sharply decreasing permeability and conductivity. The structure of the fracture network, including the number and position

of secondary fractures, significantly impacts conductivity, especially the position of secondary fractures. When the number of secondary fractures is the same, the closer they are to the outlet, the lower the energy loss caused by fluid mixing when converging into the main fracture, resulting in greater fracture conductivity. For cases with similar locations, more secondary fractures lead to greater conductivity. Due to the diversion effect of secondary fractures, the loss caused by gas flow in the fracture network is greatly reduced, resulting in higher measured conductivity. Larger sand concentration results in wider fracture width, which significantly impacts conductivity. Under the same conditions, higher fracture height increases conductivity, but the impact is not as significant as the discussed five conditions. The influence of the type of proppant on diversion capacity is the weakest.

4. Analytical investigation of fracture network conductivity

Based on experimental data of fracture network conductivity and DFN, the sand layer inside the fracture is divided into two types: the sand layer on the fracture wall and the sand layer inside the fracture. Considering factors such as proppant embedding, fragmentation, and deformation, an analytical model of single fracture conductivity is first established. Using the principle of hydroelectric similarity, an analytical model of fracture network conductivity is ultimately established, and an accuracy analysis of the analytical model is carried out.

Certain simplified assumptions are necessary regarding the internal gas flow in the fracture network and the complex sand laying method of the proppant:

1. The shape of the fracture network is fish bone-like
2. Gas flow is Darcy flow;
3. Energy loss caused by gas collisions is neglected;
4. Secondary fractures are orthogonal to the main fracture;
5. The fluid flow in the rock matrix is neglected.

4.1 A new model of conductivity of fracture network

The basic assumptions of the analytical model for the conductivity of a single fracture are as follows: The proppant is a rigid sphere that does not deform or break;

The proppant is arranged in a prismatic pattern; Based on the Kozeny-Carman model [31, 32], the proppant sand layer is treated as a capillary model (**Figure 14**).

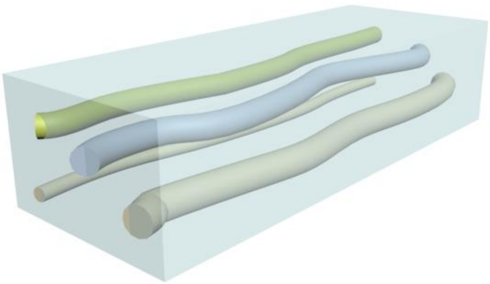

Figure 14. Schematic of the capillary porous rock physical model

The pores formed in the fracture can be divided into two types (**Figure 15**): one type is the pores near the fracture wall, which can be partially affected by the proppant embedding, the other type is the internal pores formed by the proppant, which is influenced by the proppant size.

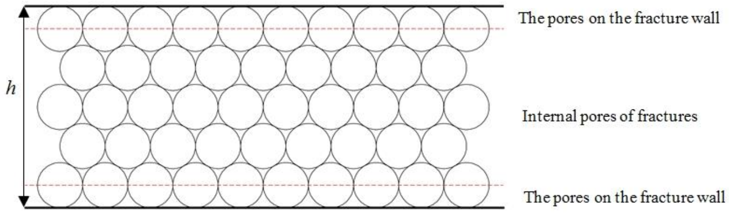

Figure 15. Schematic diagram of proppant bedding pores

(1) Calculation of the number of sand layers

$$h = c / \rho_p \tag{3}$$

Where c is sand concentration (kg/m^2), ρ_p is bulk density of proppant (kg/m^3), h is proppant stacking height (m).

$$n = \left(\frac{c / \rho_p - 2r_p}{\sqrt{3} r_p} + 1 \right) \tag{4}$$

$$m = (n)_{\text{int}} \tag{5}$$

Where n is number of sand layers, r_p is proppant radius (m), m is the integer of n.

(2) Calculation of the proppant layers inside the fracture and the proppant layer near the fracture wall

Use N to represent the total number of proppants in the sand layer. The

placement method of proppants is cumulative layer by layer, with the same number placed in each layer

$$N=\left[n\left(\frac{H-2r_p}{\sqrt{3}r_p}+1\right)-A\right]\left(\frac{L}{2r_p}\right) \tag{6}$$

Where H is fracture height (m), L is fracture length (m), A is constant, N is total number of proppant placement (integer). $m=3i-2$, $A=2i-2$; $m=3i-1$, $A=2i-1$; $m=3i$, $A=2i$; m、 I、 A=1、2、3……

Due to the difference between the proppant layers adjacent to the fracture wall and other proppant layers, its layer number needs to be calculated.

$$N_f=\left[2\left(\frac{H-2r_p}{\sqrt{3}r_p}+1\right)-B\right]\left(\frac{L}{2r_p}\right) \tag{7}$$

Where N_f is the number of proppants adjacent to the fracture wall (integer), B is constant. $m=3i-2$, $B=0$; $m=3i-1$、 $m=3i$, $B=1$.

(3) Calculation of fracture width

The stacked proppant exhibits a triangular distribution under high closing pressure. The height of adjacent layers of proppant can be calculated by $2r_p\sqrt{6}/3$. Based on the fracture height, the fracture width can be evaluated by:

$$w=(n-1)2r_p\sqrt{6}/3+2r_p \tag{8}$$

Where w is fracture width (m).

(4) Porosity calculation

The porosity of the sand layer is the ratio of the pore volume of the sand layer to the total volume of the fractures supported by the proppant. According to the definition of porosity, the calculation equation for porosity is:

$$\phi=\frac{LHw-4N\pi r_p^{\ 3}/3}{LHw} \tag{9}$$

Where ϕ is porosity of main and secondary fractures.

(5) Permeability calculation

Based on the assumption that the sand layer is viewed as a capillary model, the

relationship between permeability and pore size is obtained using the Kozeny-Carman equation.

$$K = \frac{10^{12}\phi r^2}{8\tau^2} \tag{10}$$

Where r is pore radius supporting fractures (m), τ is tortuosity (based on this configuration, it is a constant of 1.1547), K is fracture permeability (μm^2),

$$\tau = \left(\frac{L_w}{L}\right)^2 \tag{11}$$

Where L_w is actual length of capillary tube (m).

Substitute the porosity calculation equation 9 into 10 to obtain the single fracture permeability calculation equation, as follows:

$$K = \frac{\left[LHw - N\left(4\pi r_p^3/3\right)\right] r^2 \times 10^{12}}{8LHw\tau^2} \tag{12}$$

Calculation for pore radius of the propped fractures:

$$r = \frac{2}{n+1} r_1 + \frac{n-1}{n+1} r_2 \tag{13}$$

Where r_1 is fracture wall pore radius (m), r_2 is internal pore radius of fractures (m)

The pore channel near the fracture wall is enclosed by proppant hemisphere and the fracture plane, which is different from the pore channel composed of proppant inside the fracture. The calculation equation for the pore radius adjacent to the fracture wall is described as:

$$r_1 = \sqrt{\frac{2LHr_p - N_f \dfrac{2\pi r_p^{\,3}}{3}}{N_1 \pi L}} \tag{14}$$

Where N_1 is the number of pores on the fracture wall surface.

The pores inside the fracture are composed of proppants, and their calculation equation is as follows:

$$r_2 = \sqrt{\frac{LH\left[(n-1)2r_p\frac{\sqrt{6}}{3}\right]-\left(N\frac{4\pi r_p^{\ 3}}{3}-N_f\frac{2\pi r_p^{\ 3}}{3}\right)}{N_2\pi L}} \tag{15}$$

Where N_2 is number of internal pores in fracture.

$$N_1 = 2\left[\left(H-2r_p\right)/\left(\sqrt{3}r_p\right)\right]-C \tag{16}$$

$m=3i-2$, $C=0$; $m=3i-1/m=3i, C=1$.

$$N_2 = (n-1)\left[\left(H-2r_p\right)/\left(\sqrt{3}r_p\right)\right]-D \tag{17}$$

$m=3i-2/m=3i-1$, $D=2i-2$; $m=3i$, $D=2i-1$.

(6) Calculation of fracture conductivity

The equation for calculating the conductivity of a single fracture without considering embedding, proppant fragmentation, etc. is as follows:

$$Kw = \frac{\left[LHw-N\left(4\pi r_p^3/3\right)\right]r^2\times 10^{12}}{8LH\tau^2} \tag{18}$$

The breakage of proppant is not considered in the analytical model of conductivity of single fracture. Based on experimental data from the "一 type" fracture network, the analytical model for single fracture conductivity is modified.

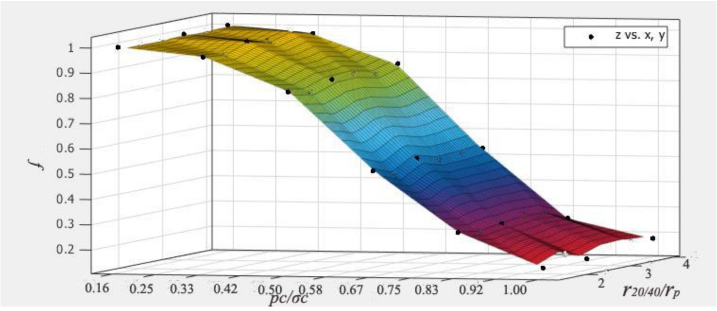

Figure 16. Results of the correction factor

Regression is performed to improve the accuracy of the derived analytical model and to apply for the facture network conductivity calculation. The z-values are the ratio of experimental measured conductivity to the calculated ones; the ratio of closure pressure to proppant compressive strength is treated as x, and the ratio of proppant radius to 20-40 mesh proppant radius are plotted as the y-axis (**Figure 16**).

$$f = 0.1946e^{-\left(\frac{P_c/\sigma_c-0.227}{0.56}\right)^2}\left(1.932\frac{r_{20/40}}{r_p}-0.9304\right) \tag{19}$$

Where f is correction factor, P_c is closure pressure (MPa), σ_c is proppant compressive strength (MPa), $r_{20/40}$ is average radius of 20-40 mesh proppant (m), r_p is average radius of proppant (m).

The modified equation for calculating the conductivity of single fracture is as follows:

$$(Kw)_f = \frac{\left[LHw - N\left(4\pi r_p^3/3\right)\right] r^2 \times 10^{12}}{8LH\tau^2} \cdot 0.1946e^{-\left(\frac{P_c/\sigma_p - 0.227}{0.56}\right)^2} \left(1.932\frac{r_{20/40}}{r_p} - 0.9304\right) \tag{20}$$

Where $(Kw)_f$ is the conductivity of single fracture considering the closure pressure, proppant fragmentation (μm^2·cm).

The principle of hydrodynamic-electrical similarity is that the seepage law of fluid flow in porous media has a certain similarity to the law of charge flow in conductive materials. These two physical processes share similar forms of governing equation and flow states.

During the experiment, a fracture network was constructed using rock slabs and proppants, as shown in Figure 17.

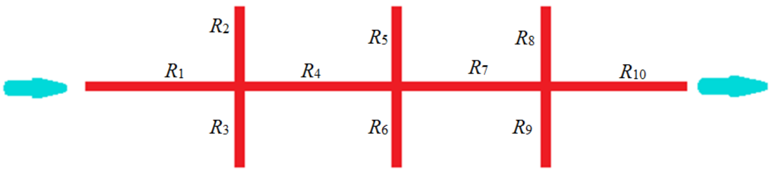

Fig.17 Schematics of "I" fracture network

Due to the equal inlet pressures of the main and secondary fractures, the incoming gas enters the fracture network from the main fracture R_1 and secondary fractures R_2, R_3, R_5, R_6, R_8, and R_9. The flow rate inside the fracture is inversely related to the seepage resistance. The gas flowing into the main fracture R_1 and secondary fractures R_2 and R_3 converges at the intersection point, and then flows into the main fracture R_4. Gas flows in other fractures, R_5~R_{10}, follows the similar patterns that are similar to the series-parallel current flow. In a parallel circuit, the potential is equal, and the current

magnitude is inversely related to the resistance. In a series circuit, the current is equal, and the potential is additive (Fig. 18).

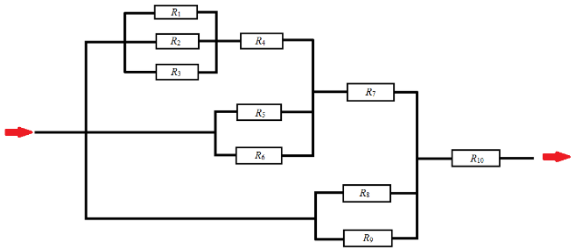

Fig.18 Equivalent circuit diagram of "I" fracture network

The total flow rate inside the chamber remains unchanged, and the inlet and outlet flow rates are equal. This is similar to the equal current in series-parallel circuits. The outlet pressure is affected by the structure of the fracture network and seepage resistance, similar to how the negative electrode potential in a circuit is affected by resistance and the structure of the circuit. Seepage resistance is influenced by factors like permeability, fracture length, and width, while electrical resistance is influenced by resistivity, resistance length, and cross-sectional area. The two are similar in this regard.

The analytical model of fracture network conductivity is established based on a single fracture conductivity model. Firstly, using the modified single fracture conductivity model (equations (3~20)), the conductivity of the main and secondary fractures is obtained. Then, using equations (21), the seepage resistance R_1~R_{3j+1} of the main and secondary fractures is calculated

$$R_k = \frac{\mu L}{(Kw)_f H} \qquad (k=1、2、3\cdots\cdots 3j+1) \tag{21}$$

Where μ is viscosity of air under standard conditions, 0.01789mPa.s, R_k is seepage resistance (mPa·s/(μm^2.cm)).

According to the equivalent circuit diagram and Ohm's law, the seepage resistance is calculated. When the secondary fracture is closing or the sand concentration is zero, the seepage resistance of the crack is infinite.

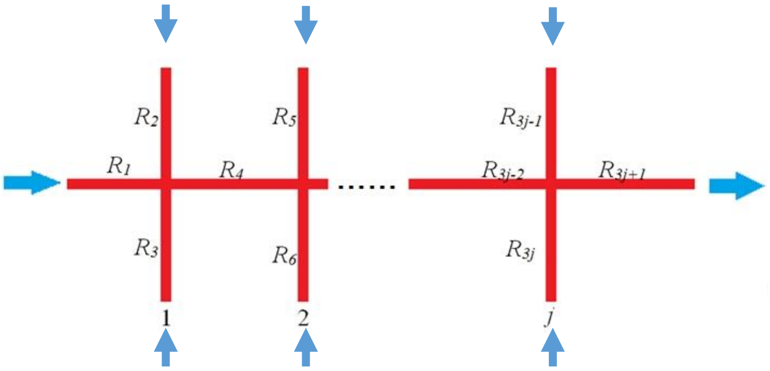

Figure 19. Schematics of shark's fin shape fracture network

As shown in **Figure 19**, there are 2j secondary fractures, and the general equation for calculating the seepage resistance is shown in Equation 22:

$$R_{t1} = \frac{1}{\frac{1}{R_1} + \frac{1}{R_2} + \frac{1}{R_3}} \qquad (j=1)$$

$$R_{tj} = \frac{1}{\frac{1}{R_{t(j-1)} + R_{3j-2}} + \frac{1}{R_{3j-1}} + \frac{1}{R_{3j}}} \qquad (j \geq 2) \tag{22}$$

$$R = R_{tj} + R_{3j+1}$$

According to equations 22 obtain the seepage resistance of complex fracture networks, and then use equations 23 to obtain the permeability of complex fracture networks.

$$K_{nw} = \frac{\mu L}{RwH} \tag{23}$$

Where K_{nw} is the permeability of complex fracture network (μm^2).

$$F_{nwc} = K_{nw} w_f \tag{24}$$

Where F_{nwc} is the conductivity of complex fracture network (μm^2.cm).

4.2 Verification and application of the fracture network conductivity model

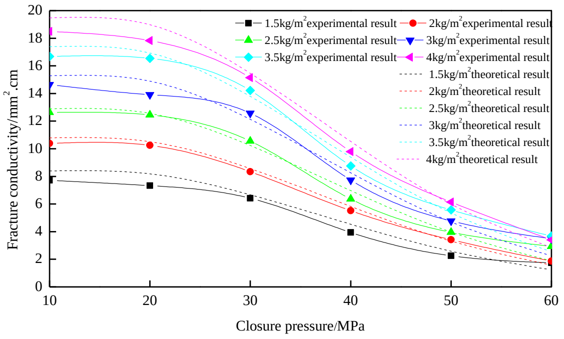

Figure 20. Comparison between theoretical and experimental results under different concentrations of "T" type fracture network

From **Figure 20**, it can be seen that the conductivity of the fracture network evaluated by the analytical model agrees well with the experimental data. The error for different sand concentrations is about 13%. When the closing pressure is less than 50 MPa, the root mean square error is 5.82%. Under high closing pressure, the error is relatively large, but the difference is small, and the error is within a reasonable range.

The fracture conductivity of different numbers of secondary fractures was calculated based on the new model.

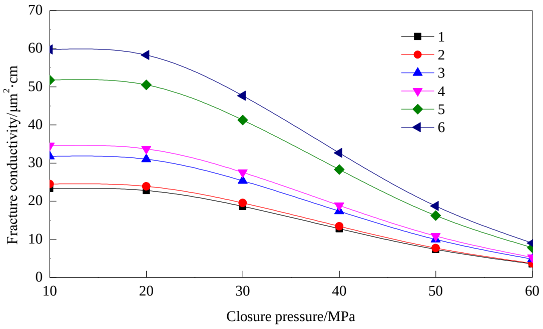

Figure 21. Conductivity of fracture network under different numbers of secondary fractures

From **Figure 21**, the more secondary fractures, the greater the conductivity of the fracture network. The increase of conductivity caused by the increase in the number of secondary fractures were 5%, 29.63%, 8.79%, 49.89%, and 15.45%, respectively. The more secondary fractures there are, the more seepage channels in the matrix increase, and the seepage resistance decreases.

Conclusions

In this study, experiments are conducted to investigate the effects of critical influential factors on the conductivity of the fracture network. A new model of fracture network conductivity is established. The following conclusion can be drawn:

1. The experiment showed that as closure pressure increases, the conductivity of the fracture network decreases. Quartz sand proppants show a similar trend, but the conductivities are lower compared with ceramics.
2. The higher the sand concentration, the greater the conductivity. As the fracture height and proppant diameter increase, the conductivity increases. The closer

the position of the secondary fracture is to the outlet, the greater the increase in the conductivity of the fracture network.

3. The orders of the conductivity is as follows: "-" type < "+"-1 type < "T" type < "+"-2 type < "F" type < "E" type < "+"-3 type < "╪" type < "Ɨ" type. Orthogonal experimental analysis shows that closure pressure and the position of secondary fractures significantly impact the conductivity of the fracture network. The proppant diameter, number of secondary fractures, and sand concentration also have a greater impact, while the type of proppant and fracture height have a weaker impact.
4. An analytical model of single fracture conductivity was established based on the Kozeny-Carman equation and experimental data. Based on the principle of hydroelectric similarity, a new model of the fracture network conductivity was ultimately established.

Conflicts of interest

The authors declare on conflict of interest.

Funding

This work was supported by the Natural Science Foundation of Sichuan Province under Grant 2023NSFSC0945.

References

[1] Lu, S., W. Huang, F. Chen, J. Li, M. Wang, H. Xue, W. WangX. Cai. 2012. Classification and evaluation criteria of shale oil and gas resources: Discussion and application. *Petroleum Exploration and Development,* 39**,** 268-276, doi: https://doi.org/10.1016/S1876-3804(12)60042-1.

[2] Nobakht, M.C. R. Clarkson. 2012. Analysis of production data in shale gas reservoirs: Rigorous corrections for fluid and flow properties. *Journal of Natural Gas Science and Engineering,* 8**,** 85-98, doi: https://doi.org/10.1016/j.jngse.2012.02.002.

[3] Hui, G., Z. Chen, R. Schultz, S. Chen, Z. Song, Z. Zhang, Y. Song, H. Wang, M. WangF. Gu. 2023. Intricate unconventional fracture networks provide fluid diffusion pathways to reactivate pre-existing faults in unconventional reservoirs. *Energy,* 282**,** 128803, doi: https://doi.org/10.1016/j.energy.2023.128803.

[4] C Chen, M., J. GengL. Zhao. 2024. A hybrid numerical model for coupled hydro-mechanical analysis during CO2 injection into heterogeneous unconventional reservoirs. *Gas Science and Engineering,* 124, 205244, doi: https://doi.org/10.1016/j.jgsce.2024.205244.

[5] Zhao, S., Q. Zhao, X. Zhang, X. Jiang, H. WuJ. Yan. 2024a. Key policy needs for the success of China's shale gas revolution. *Energy Reports,* 11, 4015-4020, doi: https://doi.org/10.1016/j.egyr.2024.03.052.

[6] Alhemdi, A.M. Gu. 2022. Method to account for natural fracture induced elastic anisotropy in geomechanical characterization of shale gas reservoirs. *Journal of Natural Gas Science and Engineering,* 101, 104478, doi: https://doi.org/10.1016/j.jngse.2022.104478.

[7] Zhao, Y., H. Yang, J. Wu, C. Liu, C. Chang, W. YuK. Sepehrnoori. 2022. Choke management simulation for shale gas reservoirs with complex natural fractures using EDFM. *Journal of Natural Gas Science and Engineering,* 107, 104801, doi: https://doi.org/10.1016/j.jngse.2022.104801.

[8] Zhao, Y., W. Xiao-Tao, C.-L. Li, Y. LiuC.-L. Xie. 2024b. Systematic prediction of the gas content, fractures, and brittleness in fractured shale reservoirs with TTI medium. *Petroleum Science*, doi: https://doi.org/10.1016/j.petsci.2024.04.015.

[9] Cipolla, C. L., N. R. Warpinski, M. J. Mayerhofer, E. P. LolonM. C. Vincent. 2008. The Relationship Between Fracture Complexity, Reservoir Properties, and Fracture Treatment Design. In *SPE Annual Technical Conference and Exhibition*.

[10] Ebrahimi, A. N., F. K. Wittel, N. A. M. AraújoH. J. Herrmann. 2014. Multi-scale approach to invasion percolation of rock fracture networks. *Journal of Hydrology,* 519, 353-363, doi: https://doi.org/10.1016/j.jhydrol.2014.07.012.

[11] Liu, Y., N. Xiu, Y. Ding, X. Wang, Y. Lu, J. Dou, Y. YanT. Liang. 2015. Analysis of multi-factor coupling effect on hydraulic fracture network in shale reservoirs. *Natural Gas Industry,* 2, 162-166, doi: https://doi.org/10.1016/j.ngib.2015.07.005.

[12] Ren, L., Z. Wang, J. Zhao, R. Lin, J. Wu, Y. SongD. Tang. 2023. Shale gas effective fracture network volume prediction and analysis based on flow back data: A case study of southern Sichuan Basin shale. *Geoenergy Science and Engineering,* 228, 211963, doi: https://doi.org/10.1016/j.geoen.2023.211963.

[13] Matthäi, S. K., C. M. Bui, H. HansikaM. S. A. Perera. 2024. Influence of inertial and centrifugal

forces on rate and flow patterns in natural fracture networks. *Advances in Water Resources*, 104741, doi: https://doi.org/10.1016/j.advwatres.2024.104741.

[14] Liu, X. 2016. Shale-gas well test analysis and evaluation after hydraulic fracturing by stimulated reservoir volume (SRV). *Natural Gas Industry B,* 3, 577-584, doi: https://doi.org/10.1016/j.ngib.2017.05.008.

[15] Zhang, Q., Y. Su, W. Wang, M. LuL. Ren. 2017. Performance analysis of fractured wells with elliptical SRV in shale reservoirs. *Journal of Natural Gas Science and Engineering,* 45, 380-390, doi: https://doi.org/10.1016/j.jngse.2017.06.004.

[16] Jiang, R., F. Zhang, Y. Cui, X. QiaoC. Zhang. 2019. Production performance analysis of fractured vertical wells with SRV in triple media gas reservoirs using elliptical flow. *Journal of Natural Gas Science and Engineering,* 68, 102925, doi: https://doi.org/10.1016/j.jngse.2019.102925.

[17] Liu, X., Y. JinB. Lin. 2022. Classification and evaluation for stimulated reservoir volume (SRV) estimation models using microseismic events based on three typical grid structures. *Journal of Petroleum Science and Engineering,* 211, 110-169, doi: https://doi.org/10.1016/j.petrol.2022.110169.

[18] Wen, Q., S. Zhang, L. Wang Y. Liu. 2005. Influenceof proppant embedment on fracture long-term flow conductivity. *Natural Gas Industry,* 25, 65-68+9-10, doi:

[19] Lu, Q., Y. He, W. Xu, R. TianF. Zhang. 2014. Volume Fracturing Fracture Network Configuration's Influence on Fracture Diverting Capacity. *Journal of Changzhou University (Natural Science Edition),* 26, 70-74, doi: 10.3969/j.issn 2095-0411.

[20] Wen, Q., S. Wang, X. Duan, Y. Li, F. WangX. Jin. 2016. Experimental investigation of proppant settling in complex hydraulic-natural fracture system in shale reservoirs. *Journal of Natural Gas Science and Engineering,* 33, 70-80, doi: https://doi.org/10.1016/j.jngse.2016.05.010.

[21] Liang, X., F. Zhou, T. Liang, Y. Huang, D. WeiS. Ma. 2020. The effect of combined proppants upon the fracture conductivity in tight gas reservoirs. *Energy Reports,* 6, 879-884, doi: https://doi.org/10.1016/j.egyr.2020.04.007.

[22] Wang, Z., B. Lin, G. Chen, Y. DaiA. Chen. 2023. Experimental study on evaluation of conglomerate reservoir support-type fracture conductivity in Xinjiang oilfield. *Energy,* 278, 127877, doi: https://doi.org/10.1016/j.energy.2023.127877.

[23] Shi , X., X. GE , Y. ZHANG, W. Huang , y. zhan , y. gu c. mou 2023. Experimental study of

propped fracture conductivity in tight dolomite reservoir. *Petroleum Geology and Recovery Efficiency,* 30**,** 167-172, doi: 10.13673/j.pgre.202204019.

[24] Katende, A., C. Allen, J. Rutqvist, S. NakagawaM. Radonjic. 2023. Experimental and numerical investigation of proppant embedment and conductivity reduction within a fracture in the Caney Shale, Southern Oklahoma, USA. *Fuel,* 341**,** 127571, doi: https://doi.org/10.1016/j.fuel.2023.127571.

[25] Liu, K., H. HeD. Yin. 2023. Theoretical and experimental study on the long-term conductivity of heterogeneous artificial fractures in tight reservoirs. *Energy Reports,* 9**,** 3881-3895, doi: https://doi.org/10.1016/j.egyr.2023.02.049.

[26] Li, K., Y. Gao, Y. LyuM. Wang. 2015. New Mathematical Models for Calculating Proppant Embedment and Fracture Conductivity. *SPE Journal,* 20**,** 496-507, doi: 10.2118/155954-PA.

[27] Liu, Z., H. Zhao, D. Wang, P. YuanY. He. 2024. Establishment and application of propped hydraulic fracture conductivity theoretical model based on fracturing efficiency index. *Gas Science and Engineering,* 121**,** 205199, doi: https://doi.org/10.1016/j.jgsce.2023.205199.

[28] Sun, Z., H. Wang, B. Zhang, Y. Li, Z. PengS. Zhang. 2024. Intelligent construction method and application of large-scale three-dimensional complex discrete fracture network model based on particle swarm optimization algorithm. Computers and Geotechnics, 171, 306-316, doi: https://doi.org/10.1016/j.compgeo.2024.106316.

[29] Nwabia, F. N.J. Y. Leung. 2024. A Practical probabilistic history matching framework for characterizing fracture network parameters of shale gas reservoirs. *Gas Science and Engineering,* 126**,** 305-326, doi: https://doi.org/10.1016/j.jgsce.2024.205326.

[30] Sun, Z., H. Wang, B. Zhang, Y. Li, Z. PengS. Zhang. 2024. Intelligent construction method and application of large-scale three-dimensional complex discrete fracture network model based on particle swarm optimization algorithm. *Computers and Geotechnics,* 171**,** 306-316, doi: https://doi.org/10.1016/j.compgeo.2024.106316.

[31] Wu, D., S. Li, N. Zhang, Y. Guo, L. LiuZ. Wang. 2023. A novel permeability model for hydrate-bearing sediments integrating pore morphology evolution based on modified Kozeny-Carman equation. *Energy,* 277**,** 127723, doi: https://doi.org/10.1016/j.energy.2023.127723.

[32] Xiao, B., H. Zhu, F. Chen, G. LongY. Li. 2023. A fractal analytical model for Kozeny-Carman

constant and permeability of roughened porous media composed of particles and converging-diverging capillaries. *Powder Technology,* 420**,** 118256, doi: https://doi.org/10.1016/j.powtec.2023.118256.

Manuscript Click here to access/download;Manuscript;Manuscript.docx

Experimental and theoretical investigation on the conductivity of complex fracture network in unconventional gas reservoirs

Jinjian Gao[1*], Lanxiao Hu[2]

1 Airport Department, Jiangxi Flight University, Nanchang, Jiangxi, China

2 College of Energy Resources, Chengdu University of Technology, Chengdu, Sichuan, China

*Corresponding author: Jinjian Gao

E-mail: gaojinjian1990@163.com

These authors contributed equally to this work.

Abstract

With the depletion of conventional oil and gas resources, unconventional oil and gas resources have become crucial as alternative hydrocarbon sources. Unconventional gas reservoirs typically have low porosity and permeability, requiring reservoir stimulation to create complex fracture network. Unlike single fracture, the fracture network exhibit complicated structures due to multiple fractures and their various distribution manners. To study the conductivity of complex fracture network, a new conductivity chamber is designed based on a discretized fracture network model. Experiments and orthogonal analysis are conducted to investigate the effect of critical influential factors such as fracture network structure, fracture height, proppant concentration, proppant diameter, and proppant type on the conductivity of complex fracture network. The results show that compared to a single fracture, the T-shape, F-shape, E-shape, ╪-shape, Ɪ –shape fracture networks conductivity increase by 49%, 131%, 150%, 188%, 201%, respectively. Closure pressure and secondary fracture distribution significantly impact the conductivity of complex fracture network. Proppant diameter and proppant concentration also have substantial effects. Compared with existing experimental studies focusing on single fracture conductivity, this study presents experimental research on the conductivity of complex fracture network, thereby addressing a research gap in this field. Through synergistic integration of the Kozeny-Carman hydrodynamic theory with hydraulic-electrical analogy principle, an innovative model of fracture network conductivity is formulated. The advantage of new model of fracture network conductivity is that it combines a theoretical method and an experimental method. This study provides valuable insights for optimizing volume fracturing in tight reservoirs, facilitating their rapid and efficient development.

Keywords: Complex Fracture Network; New Conductivity Chamber; Fracture Conductivity; Secondary Fracture; Closure Pressure; New Model of Fracture Network Conductivity

1 Introduction

Unconventional gas reservoirs generally have no natural productivity without fracturing. Hydraulic fracturing has become one of the key technologies in the development of unconventional gas reservoirs, enabling them to achieve industrial gas flows and making the development of unconventional gas reservoirs feasible [1-5]. Typically, the fractures formed in conventional reservoirs are single, symmetrical, bi-wing fractures. However, unconventional gas reservoirs have many natural fractures and possess high brittleness [6-8], which tend to create complex fracture network rather than the single fracture. These observations have been extensively found in the North American hydraulic fracturing practices [9-13]. During the fracturing process, brittle rocks and natural fractures continuously open or shear, forming a complex network of artificial and natural fractures. This approach leads to a substantial enhancement of the stimulated reservoir volume [14-17]. The technique to enhance production and ultimate recovery by creating such complex fracture network is known as volume fracturing, which plays an increasingly important role in the development of unconventional oil and gas reservoirs.

As is well known, fracture conductivity is a key parameter for volume fracturing. In recent years, experiments of fracture conductivity have evolved from single proppant types and sizes to multiple types and mixed sizes. Wen et al. [18] pioneered the use of the long-term fracture conductivity instrument in China to study proppant embedding by using formation cores. However, research object was single fracture. Lu et al. [19] simulated fracture networks using various grooves with rectangular cross-sections. They examined the conductivity of five different network structures: "一", "T", "L", "F", and "╪". It proves that the fracture network created by volume fracturing have different influence on fracture conductivity. This experiment ignored crushing and embedment of proppant. Wen et al. [20] designed a complex fracture network simulation device to study proppant settlement in "一", "T", "H", and "TF" network configurations based on discretized fracture network model. This provides a foundation for the experiment in this article. Liang et al. [21] evaluated the fracture conductivity with various ratios of sands to ceramic proppants by use of standard Fracture Conductivity Evaluation System under different effective closure stresses.

Wang et al. [22] conducted triaxial experiments to explore the effects of displacement, stress difference, and weak surfaces. Shi et al. [23] examined the fracture conductivity of tight dolomite reservoirs, analyzing various influencing factors. They found that proppant diameter and sand concentration significantly impacted fracture conductivity. Katende et al. [24] employed experimental and analytical simulation methods to study proppant embedment and fracture conductivity within Caney Shale fractures. Improved fracture conductivity is attributed to Proppant diameter. Liu et al. [25] conducted experiments on long-term non-uniform sand spreading in hydraulic fractures of tight reservoirs, considering different influencing factors. Based on the conductivity loss mechanism, the calculation model of the long-term conductivity of heterogeneous artificial fractures is established.

In order to guide hydraulic fracturing field, the single hydraulic fracture conductivity model has been established. There are two types of models, one is an empirical model based on experimental results, and the other is a theoretical model derived from fluid mechanics in porous medium and capillary model. Li et al. [26] created a new mathematical model to calculate fracture conductivity and proppant embedment. Moreover, factors affecting the fracture conductivity have been analyzed including proppant diameter, closure pressure, and proppant type. Liu et al. [27] proposed an innovative fracture conductivity calculation model based on a "fracturing efficiency index". Liu et al. [28] established a new model for predicting the fracture conductivity considering the particle size redistribution caused by proppant crushing based on Kozeny–Carman equation. However, the most important point has been overlooked: the fracture network is composed of multiple fractures.

In previous research, experimental research on fracture conductivity has mainly focused on single fracture. The experimental instrument for fracture conductivity is still traditional diversion chamber which cannot meet the requirements of testing the conductivity of complex fracture network. There is very little research on the calculation model of the conductivity of fracture network.

Firstly, a new diversion chamber was designed and manufactured based on a discrete fracture network model. Then, rock slab is combined into fracture network.

Experiments with different fracture structures will be conducted using a new type of diversion chamber. Orthogonal experiments were used to study the primary and secondary factors affecting the conductivity of complex fracture network. Finally, based on the Kozeny-Carman equation and the principle of hydraulic-electrical analogy, a new model of fracture network conductivity is established.

2 Experimental methods

Traditional conductivity measurement chambers can only test the conductivity of a single fracture, falling short of testing the conductivity of complex fracture network. In this study, we propose a new conductivity measurement chamber based on the experimental observations obtained from the settlement and transportation of proppant in the complex fracture networks and discrete fracture networks [29, 30]. This new chamber features a larger inner cavity space enabling the investigation of fracture networks and can be integrated with the fracture conductivity measurement setup.

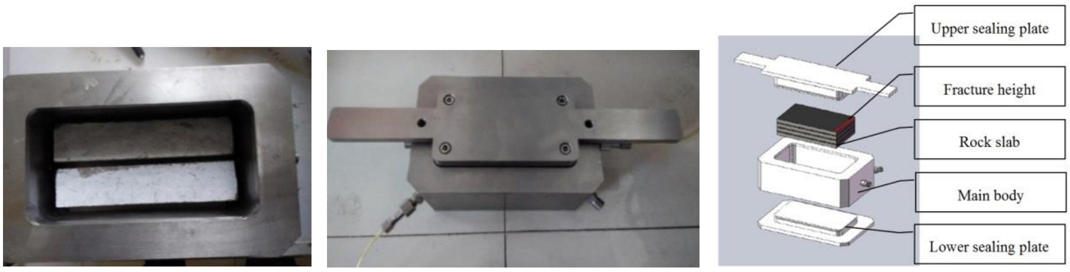

(a) Internal diagram **(b) External image** **(c) Explosion view**

Fig 1. Structure diagram of new conductivity measurement chamber

Fig 1 illustrates the internal structure of the new conductivity measurement chamber with a dimension of 170mm×90mm×50mm. The chamber is equipped with stainless steel pads to adjust the fracture height. The compressive strength of stainless steel is 520 MPa. Various shapes of rock slabs simulate different fracture networks, the surrounding space is sealed with foam rubber. There are four interfaces to monitor the inlet and outlet pressures and flow rates. Compared with the standard conductivity measurement chamber, the new proposed chamber possesses the following features:

(1) The diversion chamber, constructed from stainless steel, undergoes wire cutting internally to ensure smooth cavity walls.

(2) The rock slab is positioned 10mm from the edge wall on one side. This arrangement ensures even gas distribution at the fracture's inlet and outlet, mitigating the end-effect and enhancing test accuracy.

(3) Due to the application of sealant on both sides of the rock slab, pressure measurement on the walls is not feasible. Pressure measuring ports are located on the inlet and outlet side walls to obtain the pressure differences. The effective fracture length is 160mm.

(4) Filter plugs are installed in the gas inlet and pressure measuring ports to prevent the leakage of the crushed proppant during the experimental process.

(5) The maximum closure pressure is 100 MPa.

The experimental instrument primarily comprises six components, as illustrated in Fig 2.

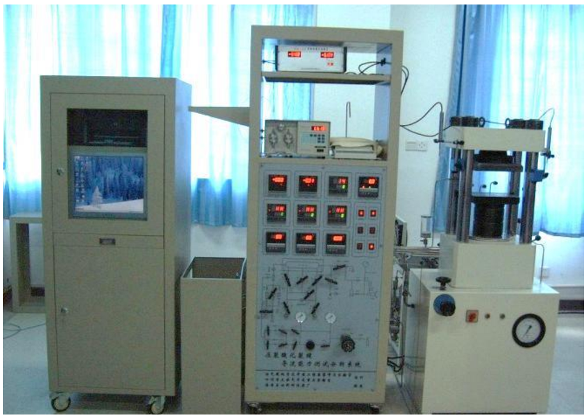

Fig 2. Fracture conductivity instrument of FCES-100

This experiment uses the volume equivalence principle, where the void volume in the fracture network equates to an imaginary single fracture's volume. The fracture network width is assumed to be the same as the width of the imaginary single fracture. This width is calculated by dividing the total volume by the surface area parallel to the seepage direction:

$$w_f = V / S \tag{1}$$

Where w_f is equivalent fracture width (cm), V is proppant volume (cm^3), S is horizontal area of fracture network (cm^2).

Calculation equation for fracture conductivity can be written as:

$$kw_f = \frac{Q\mu Lw_f}{A_f \Delta P} \quad (2)$$

Where k is permeability (μm^2), Q is flow rate(cm^3/s), μ is gas viscosity (mPa·s), L is fracture conductivity length (cm), A_f is cross section area of fracture network (cm^2), ΔP is pressure difference between inlet and outlet (0.1MPa).

We construct several typical fracture network structures based on the discretized fracture network model. Fig 3 summarizes these structures, including "─" type, "T" type, "F" type, "E" type, "+" type, "╪" type, and "Ɨ" type. For the conductivity measurements, it is assumed that the width of the secondary fracture is equal to that of the main fracture.

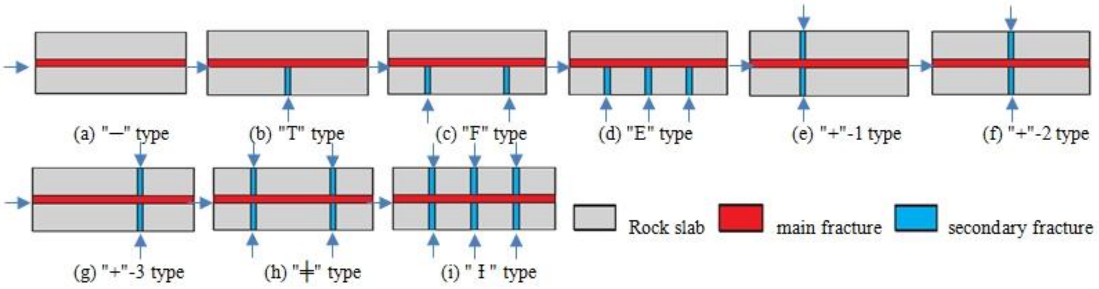

Fig 3. Schematics of different types of fracture networks

Taking the "─" type fracture network as an example, the experimental scheme for changing the sand concentration is as follows: the fracture height is 50mm, and the sand concentrations are 1.5, 2, 2.5, 3, 3.5, and 4 kg/m^2. Because unconventional reservoirs commonly employ low sand ratios and small-sized proppants during fracturing operations [31]. The proppant is 20-40 mesh ceramic proppant. The experimental scheme for changing the fracture height is as follows: the fracture heights are 23, 36 and 50 mm, with a sand concentration of 4 kg/m^2. The rock slabs provided by the laboratory can only be processed into sizes of 23, 36, and 50mm. The length-to-height ratio of fractures was designed based on field observations and theoretical frameworks widely adopted in unconventional reservoir engineering. Specifically, we focused on a range of 0.14 to 0.35 for this ratio, which aligns with typical fracture geometries observed in shale and tight sandstone reservoirs under similar stress conditions. The proppant used is 20-40 mesh ceramic proppant. The experimental scheme for changing the proppant

diameter is as follows: the fracture height is 50 mm, the sand concentration is 4 kg/m², and the proppants are 20-40, 40-70, and 80-100 mesh ceramic proppants. For the experimental scheme involving different types of proppants, only the proppant type is changed to ceramsite and quartz sand. The experimental scheme for other fracture structures is similar to that of the "—" type fracture.

Experimental procedure: Cut the rock slab and combine it into a fracture network. Place a specific amount of proppant in the fractures. Seal the gap and unpropped fracture with sealant, and install the sealing plate in the measurement chamber. Connect the inlet and outlet pressure and flow ports to the chamber. Pressurize the diversion chamber with closure pressures of 10, 20, 30, 40, 50, and 60MPa.

3 Results and discussion

3.1 Experimental results of "—type" fracture network

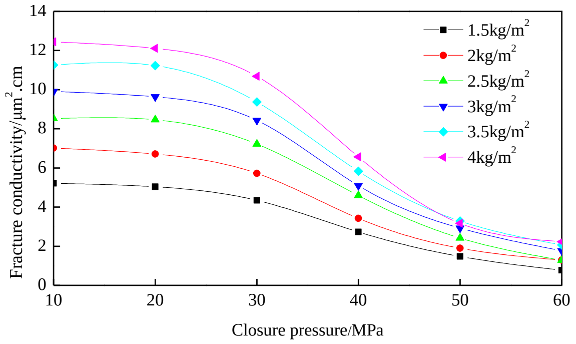

Fig 4. Fracture conductivity of "—" fracture network with different ceramic concentrations

Fig 4 shows the conductivity of fracture network under different closure pressures and proppant concentrations. The trend of the fracture network's conductivity with closure pressure is similar across different proppant concentrations. As closure pressure increases, the conductivity of the fracture network decreases. When the proppant concentration is 4 kg/m², the conductivity is 12.446 μm²·cm at 10 MPa, decreases to 10.681 μm²·cm at 30 MPa, and drops to 2.228 μm²·cm at 60 MPa. Based on the analysis of six sets of curves, the decrease in conductivity under high closure pressure (60MPa) is between 81.7% and 85.1% compared to low closure pressures (10MPa). At low closure pressure, the proppant stays intact. The large pores formed by the proppant results in high permeability and greater fracture conductivity. The decrease in

conductivity is due to the elastic deformation of the proppant, which slightly reduces pore size. At high closure pressure, the proppant breaks. The broken ceramic proppant has low porosity, resulting in lower permeability and significantly reduced fracture conductivity. In this experiment, rock slabs with high brittleness and high Young's modulus were used. There was less proppant embedment, and the fracture conductivity was greater than that of rock slabs with low Young's modulus. The closure pressure is generally the minimum principal stress. The smaller the minimum stress is, the greater the conductivity capacity will be.

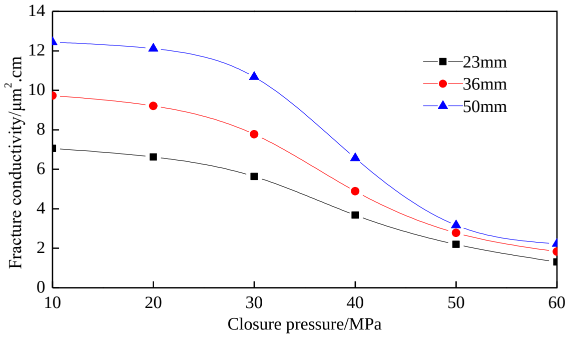

Fig 5. Fracture conductivity of "—" fracture network with different fracture heights

From Fig 5, it can be observed that an increase in closure pressure leads to a decrease in fracture conductivity. The conductivity of the fracture network increases with fracture height. The fracture conductivity increases exponentially with fracture height. To increase fracture height in the deep part of the fracture, it's necessary to use high displacement and high viscosity fracturing fluid to carry the proppant, forming a high and long propped fracture to improve the conductivity.

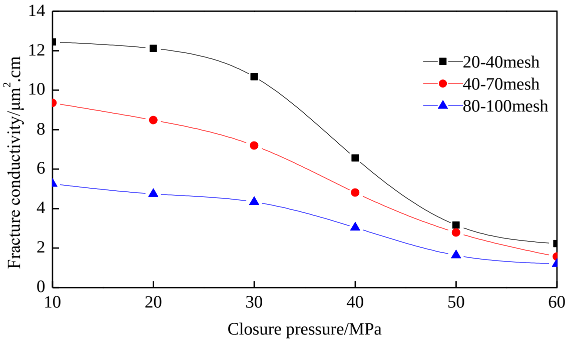

Fig 6. Fracture conductivity of "—" fracture network with different proppant diameters

Fig 6 shows that the conductivity of fracture network increases with the proppant diameter. The 20-40 mesh proppant has the highest conductivity, while the 80-100

mesh proppant has the lowest due to its smaller particle diameter. The difference in conductivity among the three proppant diameters is significant under low-pressure conditions but less notable under high-pressure conditions. Due to the complex structure and various fracture widths of fracture network, larger proppant cannot easily enter the small fractures. Based on the experimental results, the optimal Proppant diameter for volume fracturing is 40-70 mesh, which has a smaller decrease in conductivity compared to the 20-40 mesh proppant and can be carried into the narrows fractures.

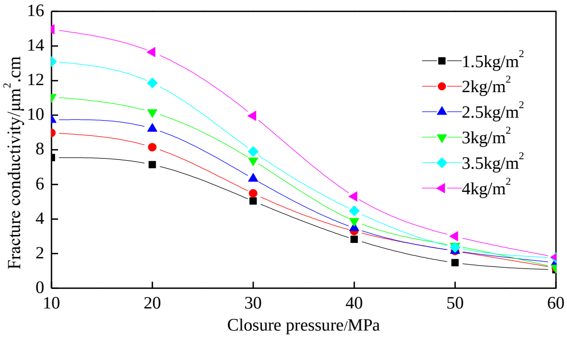

Fig 7. Fracture conductivity of "—" fracture network with different quartz sand concentrations

Comparing Fig 4 and Fig 7, the fracture conductivity of quartz sand at 10 MPa is slightly higher than that of ceramic proppant. However, under high pressure, it is lower than that of ceramic proppant. The fracture conductivity decreases by 92.1% with the closure pressure, indicating that the quartz sand possess lower conductivity. It can be concluded that when the closure pressure is greater than 30 MPa, quartz sand cannot effectively provide higher conductivity and is unsuitable for high closure pressure conditions.

3.2 The influence of fracture network structure on conductivity

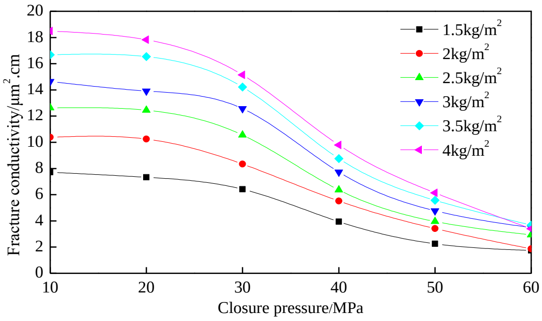

(a) Concentration of ceramic proppant

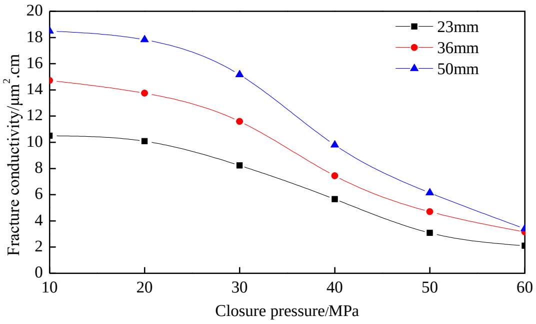

(b) Fracture height

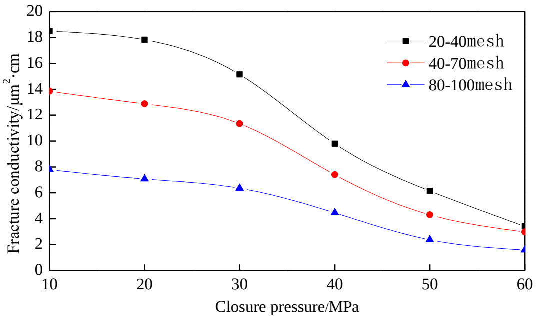

(c) Proppant diameter

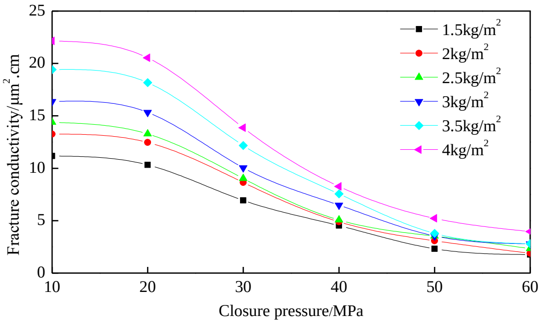

(d) Concentration of quartz sand

Fig 8. Experimental results of "T" fracture network

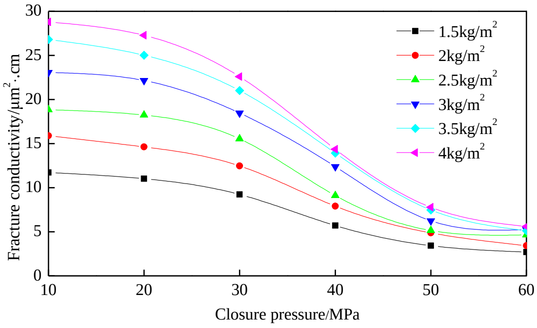

(a) Concentration of ceramic proppant

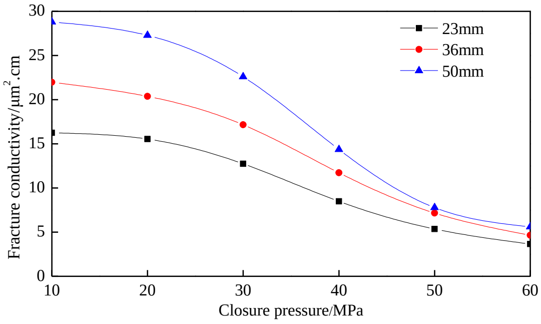

(b) Fracture height

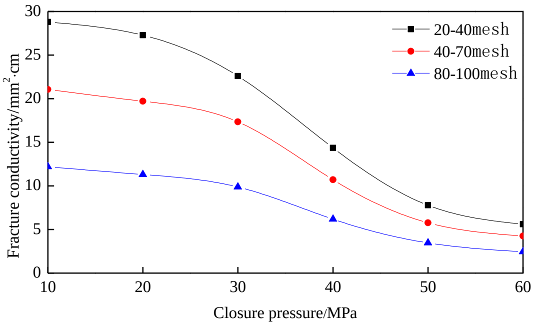

(c) Proppant diameter

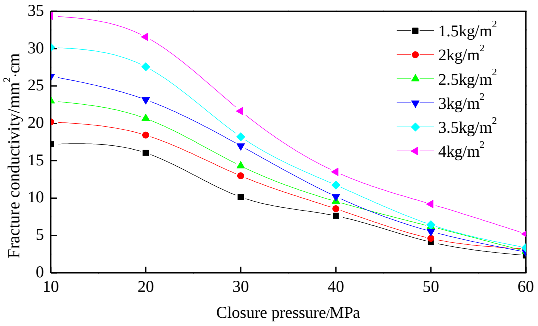

(d) Concentration of quartz sand

Fig 9. Experimental results of "F" fracture network

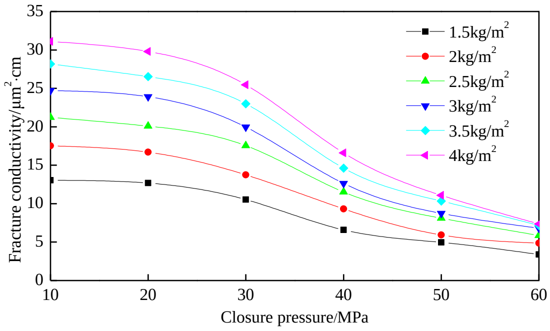

(a) Concentration of ceramic proppant

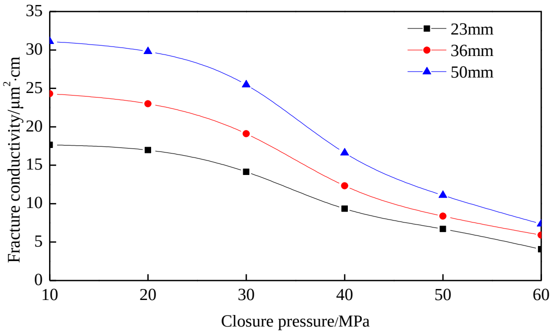

(b) Fracture height

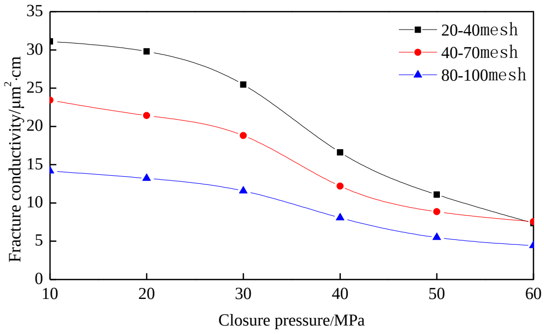

(c) Proppant diameter

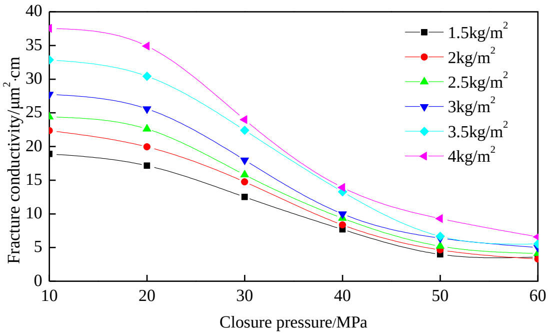

(d) Concentration of quartz sand

Fig 10. Experimental results of "E" fracture network

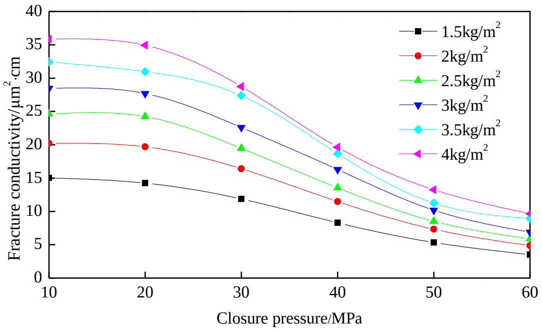

(a) Concentration of ceramic proppant

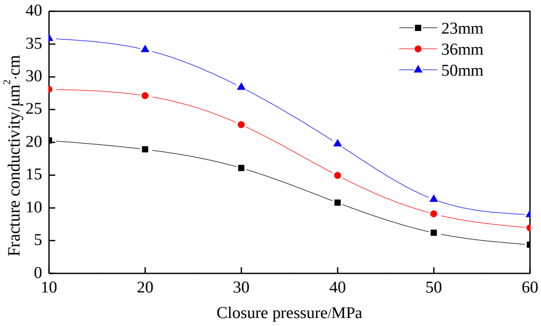

(b) Fracture height

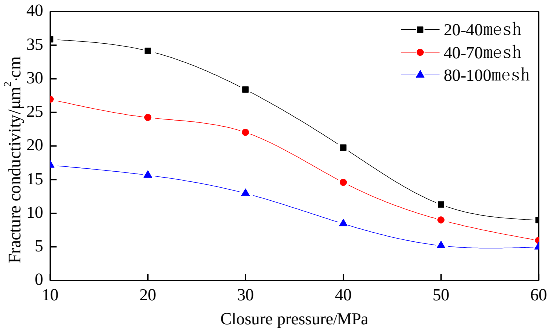

(c) Proppant diameter

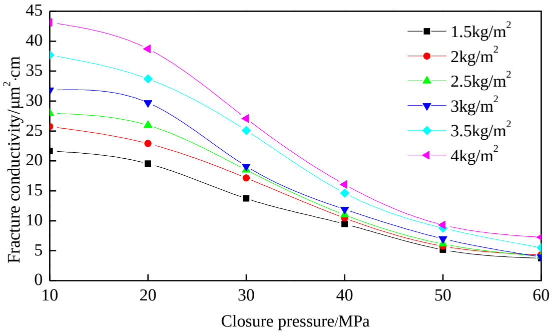

(d) Concentration of quartz sand

Fig 11. Experimental results of "╪" fracture network

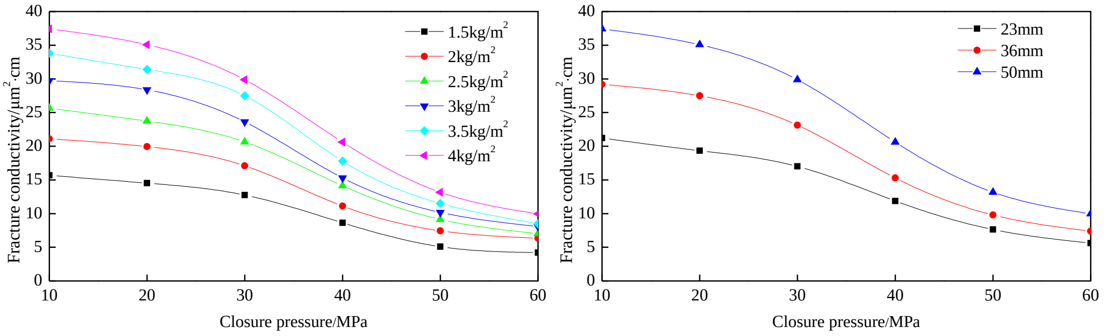

(a) Concentration of ceramic proppant **(b) Fracture height**

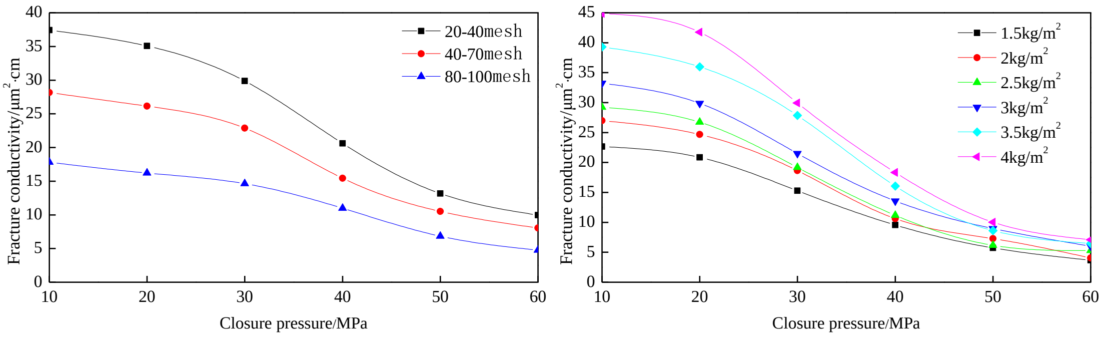

(c) Proppant diameter **(d) Concentration of quartz sand**

Fig 12. Experimental results of “Ɨ” fracture network

Fig 8-12 shows the conductivity of different fracture networks under different conditions.

The conductivity of different types of fracture networks are analyzed under the same conditions.

Table 1. Influence of secondary fracture number on fracture conductivity

Fracture network	"T" type	"F" type	"E" type	"╪" type	" Ɨ" type
Number of secondary fractures	1	2	3	4	6
Initial fracture conductivity growth factor	1.49	2.31	2.50	2.88	3.01
Final conductivity /Initial conductivity	18.4%	19.5%	23.7%	26.9%	29.3%

From Table 1, it can be seen that compared to the "─ type" fracture network, the initial fracture conductivity continuously increases with the number of secondary fractures. The growth factors of the fracture network conductivity are 1.49, 2.31, 2.50, 2.88, and 3.01 for "T", "F", "E", "╪", and "Ɨ" types, respectively. The ratio of the final conductivity (60MPa) to the initial conductivity (10MPa) also indicates that more secondary fractures lead to greater final fracture conductivity. Sufficient secondary fractures under high pressure can ensure that the formation maintains a certain degree of diversion capacity.

Table 2. Influence of secondary fracture position on fracture conductivity

Fracture network	“+”-1 type	“+”-2 type	“+”-3 type
Distance from air outlet /mm	120	80	40
Initial fracture conductivity growth factor	1.20	1.66	2.82
Final conductivity /Initial conductivity	20.6%	22.1%	24.9%

From Table 2, it can be evidently observed that the nearer the secondary fractures are to the outlet of the chamber, the more significant the increase in the conductivity of the fracture network. From the ratio of the final conductivity (60MPa) to the initial conductivity (10MPa), it can be observed that the closer the secondary fractures are to the outlet, the greater the conductivity of the fracture network under high pressure. The closer the secondary fracture is to the outlet, the smaller the resistance during the flow process and the lower the energy loss in the secondary fractures. When the fluid enters the main fracture, the closer it is to the outlet, the lower the energy of the fluid in the main fracture, which means that the energy loss of mixing the fluid in the main and secondary fractures is the smallest and the increase in fracture conductivity is the largest.

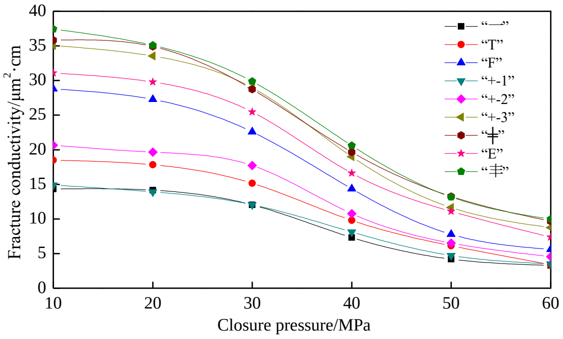

Fig 13. Comparison of the conductivity of nine types of fracture network

Using the 20-40 mesh ceramic proppant, with a sand concentration of 4 kg/m² and a fracture height of 50 mm, conductivity obtained from nine types of fracture network were plotted in Fig 13. The order of conductivity is as follows: "—" type < "+"-1 type < "T" type < "+"-2 type < "F" type < "E" type < "+"-3 type < "╪" type < "Ɨ" type. The following observations have been found: (1) The conductivity of the "—" and "+"-1 fracture networks are close to each other and exhibit lowest conductivity values. Although the "+"-1 fracture network has two secondary fractures, their locations are far away from the outlet, resulting in high pressure consumption during flow and

significant energy loss when the fluid converges from the secondary fractures into the main fracture. (2) The conductivity of the "+"-2 fracture network is slightly higher than that of the "T" fracture network. The secondary fracture positions are the same, but the "+"-2 fracture network has an additional secondary fracture. (3) The conductivity of the "F" and "E" fracture networks is similar and significantly improved. Both have one secondary fracture closest to the outlet, indicating that the position of the secondary fracture significantly impacts the conductivity. (4) The conductivity of the "+"-3, "╪", and "Ɨ" types of fracture networks is the highest because they have two secondary fractures closest to the outlet. The "Ɨ" type has 6 secondary fractures, the "╪" type has 4, and the "+"-3 type has 2. Therefore, the "Ɨ" type has the highest conductivity, followed by the "╪" type, and the "+"-3 type has the lowest among the top three.

3.3 Orthogonal experimental analysis

This study considered seven influencing factors with the assumption of no interactions between them. Among these factors, proppant type was assigned two distinct levels, while the remaining six factors each had three levels. Through orthogonal array reference tables, the optimal experimental layout was identified as $L_{18}(2\times3^7)$. The designed orthogonal experiments evaluated outcomes using conductivity as the performance metric, with detailed results provided in Table 3.

Table 3. Results of the orthogonal experiments

Number	Proppant type	Number of secondary fractures	Distance from air outlet /mm	Fracture height /mm	Sand concentration /(kg/m^2)	Proppant diameter /mesh	Closure pressure /MPa	Error	Conductivity /μm^2·cm
1	Ceramsite	1	120	23	2	20-40	20	1	4.623
2		1	80	36	3	40-70	40	2	5.027
3		1	40	50	4	80-100	60	3	2.994
4		2	120	23	3	40-70	60	3	1.215
5		2	80	36	4	80-100	20	1	6.948
6		2	40	50	2	20-40	40	2	10.936
7		4	120	36	2	80-100	40	3	2.073
8		4	80	50	3	20-40	60	1	6.875
9		4	40	23	4	40-70	20	2	15.804
10	Quartz sand	1	120	50	4	40-70	40	1	6.305
11		1	80	23	2	80-100	60	2	1.147
12		1	40	36	3	20-40	20	3	17.339

13		2	120	36	4	20-40	60	2	1.674
14		2	80	50	2	40-70	20	3	6.624
15		2	40	23	3	80-100	40	1	3.402
16		4	120	50	3	80-100	20	2	6.378
17		4	80	23	4	20-40	40	3	8.627
18		4	40	36	2	40-70	60	1	2.774

The F critical value is selected according to the F value critical value table with a confidence interval of 0.01. According to the verification results in Table 4, the closure pressure and the position of secondary fractures have the greatest impact on the conductivity of the fracture network. The proppant diameter and the sand concentration also significantly affect conductivity, while the number of secondary fractures, proppant type and the fracture height exhibit a weaker impact.

Table 4. Analyses of the variances

Source of variance	Sum of squares	free degree	Mean sum of squares	F value	F critical value	Significance
Proppant type	0.275	1	0.275	0.088	3.46	
Number of secondary fractures	11.536	2	5.768	1.851	3.11	
Distance from air outlet	80.685	2	40.343	12.945	3.11	High saliency
Fracture height	2.631	2	1.315	0.422	3.11	
Sand concentration	19.490	2	9.745	3.127	3.11	Saliency
Proppant diameter	61.517	2	30.758	9.870	3.11	Saliency
Closure pressure	140.412	2	70.206	22.527	3.11	High saliency
Error	9.349	3	3.116			

Under high closure pressure, the proppant is crushed, leading to a significant reduction in fracture width and pore size, sharply decreasing permeability and conductivity. The structure of fracture network, including the number and position of secondary fractures, significantly impacts conductivity, especially the position of secondary fractures. Fracture conductivity increases as secondary fractures approach the outlet, with closer positioning minimizing fluid mixing-induced energy losses under equivalent fracture. Under comparable spatial configurations, an increase in secondary fracture number directly enhances fracture network conductivity. The flow diversion effect created by secondary fractures significantly mitigates gas flow energy dissipation within the fracture network, thereby enhancing the fracture conductivity. Larger sand concentration results in wider fracture width, which significantly enhances conductivity. Under the same conditions, higher fracture height increases conductivity,

but the impact is not as significant as the discussed five conditions. The influence of the type of proppant on fracture conductivity is the weakest.

4 Analytical investigation of fracture network conductivity

Based on experimental data of fracture network conductivity and discretized fracture network model, the sand layer inside the fracture is divided into two types: the sand layer on the fracture wall and the sand layer inside the fracture. Considering factors such as proppant embedding, fragmentation, and deformation, an analytical model of single fracture conductivity is first established. Leveraging the hydraulic-electrical analogy principle, a theoretical model for fracture network conductivity is rigorously derived through multiphysical coupling analysis. The accuracy analysis of the analytical model is carried out.

Certain simplified assumptions are necessary regarding the internal gas flow in the fracture network and the complex sand laying method of the proppant:

1. The shape of the fracture network is fish bone-like
2. Gas flow is Darcy flow;
3. Energy loss caused by gas collisions is neglected;
4. Secondary fractures are orthogonal to the main fracture;
5. The fluid flow in the rock matrix is neglected.

4.1 A new model of conductivity of fracture network

The basic assumptions of the analytical model for the conductivity of a single fracture are as follows: The proppant is a rigid sphere that does not deform or break; The proppant is arranged in a prismatic pattern. Based on the Kozeny-Carman model [32, 33], the proppant sand layer is treated as a capillary model (Fig 14). The new model assumes that all capillary are horizontal and uniform. In reality, both horizontal and vertical capillaries exist within the proppant. At low pressure, gas predominantly mainly flows along horizontal directions. Under high closure pressures, proppant crushing increases gas flow through vertical capillaries, leading to the decrease of the model's accuracy.

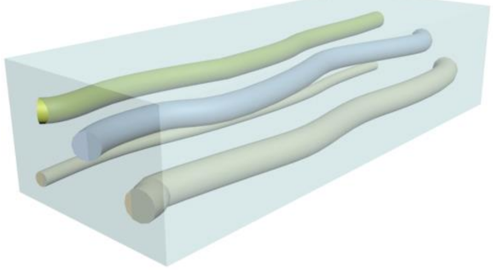

Fig 14. Schematic of the capillary porous rock physical model

The pores formed in the fracture can be divided into two types (Fig 15): one type is the pores near the fracture wall, which can be partially affected by the proppant embedding. The other type is the internal pores formed by the proppant, which is influenced by the Proppant diameter.

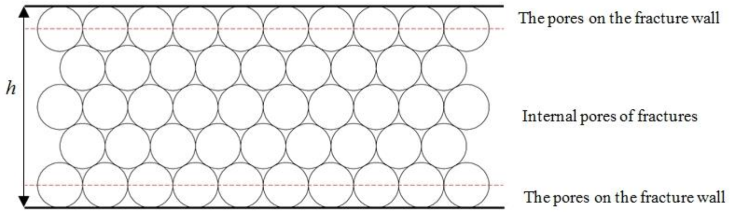

Fig 15. Schematic diagram of proppant bedding pores

(1) Calculation of the number of sand layers

$$h = c / \rho_p \tag{3}$$

Where c is proppant concentration (kg/m^2), ρ_p is bulk density of proppant (kg/m^3), h is proppant stacking height (m).

$$n = \left(\frac{c / \rho_p - 2r_p}{\sqrt{3} r_p} + 1 \right) \tag{4}$$

$$m = (n)_{\text{int}} \tag{5}$$

Where n is number of sand layers, r_p is proppant radius (m), m is the integer of n.

(2) Calculation of the proppant layers inside the fracture and the proppant layer near the fracture wall

Use N to represent the total number of proppants in the sand layer. The placement method of proppants is cumulative layer by layer, with the same number

placed in each layer

$$N=\left[n\left(\frac{H-2r_p}{\sqrt{3}r_p}+1\right)-A\right]\left(\frac{L}{2r_p}\right) \tag{6}$$

Where H is fracture height (m), L is fracture length (m), A is constant, N is total number of proppant placement (integer). $m=3i-2$, $A=2i-2$; $m=3i-1$, $A=2i-1$; $m=3i$, $A=2i$; m、 I、 A=1、2、3……

Due to the difference between the proppant layers adjacent to the fracture wall and other proppant layers, its layer number needs to be calculated.

$$N_f=\left[2\left(\frac{H-2r_p}{\sqrt{3}r_p}+1\right)-B\right]\left(\frac{L}{2r_p}\right) \tag{7}$$

Where N_f is the number of proppants adjacent to the fracture wall (integer), B is constant. $m=3i-2$, $B=0$; $m=3i-1$、 $m=3i$, $B=1$.

(3) Calculation of fracture width

The stacked proppant exhibits a triangular distribution under high closure pressure. The height of adjacent layers of proppant can be calculated by $2r_p\sqrt{6}/3$. Based on the fracture height, the fracture width can be evaluated by:

$$w=(n-1)2r_p\sqrt{6}/3+2r_p \tag{8}$$

Where w is fracture width (m).

(4) Porosity calculation

The porosity of the sand layer is the ratio of the pore volume of the sand layer to the total volume of the fractures supported by the proppant. According to the definition of porosity, the calculation equation for porosity is:

$$\phi=\frac{LHw-4N\pi r_p^{\,3}/3}{LHw} \tag{9}$$

Where ϕ is porosity of main and secondary fracture.

(5) Permeability calculation

Based on the assumption that the sand layer is viewed as a capillary model, the relationship between permeability and pore size is obtained using the Kozeny-Carman

equation.

$$K=\frac{10^{12}\phi r^2}{8\tau^2} \tag{10}$$

Where r is pore radius supporting fractures (m), τ is tortuosity (based on this configuration, it is a constant of 1.1547), K is fracture permeability (μm^2).

$$\tau=\left(\frac{L_w}{L}\right)^2 \tag{11}$$

Where L_w is actual length of capillary tube (m).

Substitute the porosity calculation equation 9 into 10 to obtain the single fracture permeability calculation equation, as follows:

$$K=\frac{\left[LHw-N\left(4\pi r_p^3/3\right)\right]r^2\times10^{12}}{8LHw\tau^2} \tag{12}$$

Calculation for pore radius of the propped fractures:

$$r=\frac{2}{n+1}r_1+\frac{n-1}{n+1}r_2 \tag{13}$$

Where r_1 is fracture wall pore radius (m), r_2 is internal pore radius of fractures (m)

The pore channel near the fracture wall is enclosed by proppant hemisphere and the fracture plane, which is different from the pore channel composed of proppant inside the fracture. The calculation equation for the pore radius adjacent to the fracture wall is described as:

$$r_1=\sqrt{\frac{2LHr_p-N_f\dfrac{2\pi r_p{}^3}{3}}{N_1\pi L}} \tag{14}$$

Where N_1 is the number of pores on the fracture wall surface.

The pores inside the fracture are composed of proppants, and their calculation equation is as follows:

$$r_2=\sqrt{\frac{LH\left[(n-1)2r_p\dfrac{\sqrt{6}}{3}\right]-\left(N\dfrac{4\pi r_p{}^3}{3}-N_f\dfrac{2\pi r_p{}^3}{3}\right)}{N_2\pi L}} \tag{15}$$

Where N_2 is number of internal pores in fracture.

$$N_1 = 2\left[\left(H-2r_p\right)/\left(\sqrt{3}r_p\right)\right]-C \tag{16}$$

$m=3i-2$, $C=0$; $m=3i-1/m=3i, C=1$.

$$N_2 = (n-1)\left[\left(H-2r_p\right)/\left(\sqrt{3}r_p\right)\right]-D \tag{17}$$

$m=3i-2/m=3i-1$, $D=2i-2$; $m=3i$, $D=2i-1$.

(6) Calculation of fracture conductivity

The equation for calculating the conductivity of a single fracture without considering embedding, proppant fragmentation, etc. is as follows:

$$Kw = \frac{\left[LHw - N\left(4\pi r_p^3/3\right)\right]r^2 \times 10^{12}}{8LH\tau^2} \tag{18}$$

The breakage of proppant is not considered in the analytical model of conductivity of single fracture. Based on experimental data from the "一 type" fracture network, the analytical model for single fracture conductivity is modified.

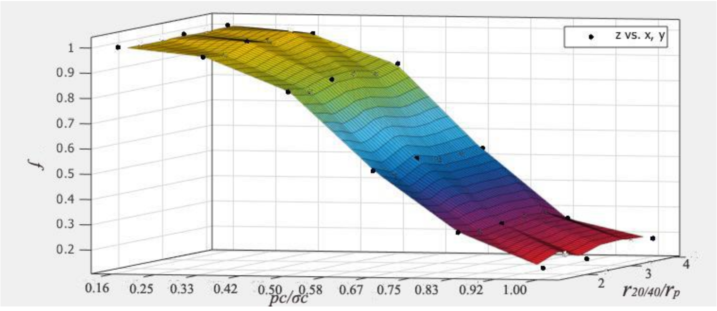

Fig 16. Results of the correction factor

Regression is performed to improve the accuracy of the derived analytical model and to apply for the facture network conductivity calculation. The z-values are the ratio of experimental measured conductivity to the calculated ones; the ratio of closure pressure to proppant compressive strength is treated as x; the ratio of proppant radius to 20-40 mesh proppant radius are plotted as the y-axis (Fig 16).

$$f = 0.1946e^{-\left(\frac{P_c/\sigma_c - 0.227}{0.56}\right)^2}\left(1.932\frac{r_{20/40}}{r_p} - 0.9304\right) \tag{19}$$

Where f is correction factor, P_c is closure pressure (MPa), σ_c is proppant compressive strength (MPa), $r_{20/40}$ is average radius of 20-40 mesh proppant (m), r_p

is average radius of proppant (m).

The modified equation for calculating the conductivity of single fracture is as follows:

$$(Kw)_f = \frac{\left[LHw - N\left(4\pi r_p^3/3\right)\right] r^2 \times 10^{12}}{8LH\tau^2} \cdot 0.1946 e^{-\left(\frac{P_c/\sigma_p - 0.227}{0.56}\right)^2} \left(1.932\frac{r_{20/40}}{r_p} - 0.9304\right) \tag{20}$$

Where $(Kw)_f$ is the conductivity of single fracture considering the closure pressure, proppant fragmentation ($\mu m^2 \cdot cm$).

The principle of hydraulic-electrical analogy is that the seepage law of fluid flow in porous media has a certain similarity to the law of charge flow in conductive materials. These two physical processes share similar forms of governing equation and flow states.

During the experiment, a fracture network was constructed using rock slabs and proppants, as shown in Fig 17.

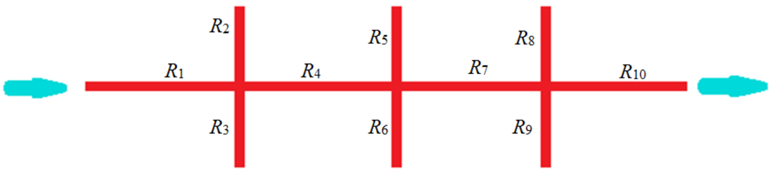

Fig 17. Schematics of "I" fracture network

Due to the equal inlet pressures of the main and secondary fractures, the incoming gas enters the fracture network from the main fracture R_1 and secondary fractures R_2, R_3, R_5, R_6, R_8, and R_9. The flow rate inside the fracture is inversely related to the seepage resistance. The gas flowing into the main fracture R_1 and secondary fractures R_2 and R_3 converges at the intersection point, and then flows into the main fracture R_4. Gas flows in other fractures, R_5~R_{10}, follows the similar patterns that are similar to the series-parallel current flow. In a parallel circuit, the potential is equal, and the current magnitude is inversely related to the resistance. In a series circuit, the current is equal, and the potential is additive (Fig. 18).

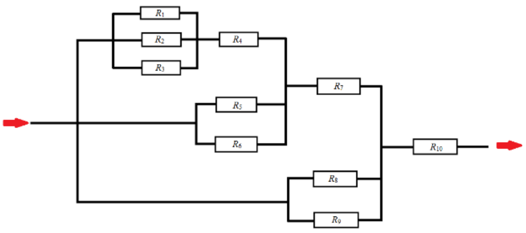

Fig 18. Equivalent circuit diagram of "I" fracture network

The total flow rate inside the chamber remains unchanged, and the inlet and outlet flow rates are equal. This is similar to the equal current in series-parallel circuits. The outlet pressure is affected by the structure of the fracture network and seepage resistance, similar to how the negative electrode potential in a circuit is affected by resistance and the structure of the circuit. Seepage resistance is influenced by factors like permeability, fracture length, and width, while electrical resistance is influenced by resistivity, resistance length, and cross-sectional area. The two are similar in this regard.

The analytical model of fracture network conductivity is established based on a single fracture conductivity model. Firstly, using the modified single fracture conductivity model (equations (3~20)), the conductivity of the main and secondary fractures is obtained. Then, using equations (21), the seepage resistance R_1~R_{3j+1} of the main and secondary fractures is calculated.

$$R_k = \frac{\mu L}{(Kw)_f H} \qquad (k=1、2、3\cdots\cdots 3j+1) \qquad (21)$$

Where μ is viscosity of air under standard conditions, 0.01789mPa.s, R_k is seepage resistance (mPa·s/(μm^2.cm)).

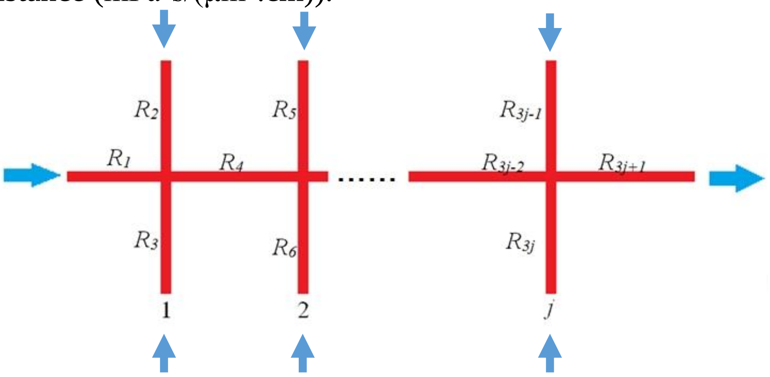

Fig 19. Schematics of shark's fin shape fracture network

According to the equivalent circuit diagram and Ohm's law, the seepage resistance is calculated. When the secondary fracture is closing or the sand concentration is zero, the seepage resistance of the crack is infinite.

As shown in Fig 19, there are 2j secondary fractures, and the general equation for calculating the seepage resistance is shown in equation 22:

$$R_{t1} = \frac{1}{\frac{1}{R_1} + \frac{1}{R_2} + \frac{1}{R_3}} \qquad (j=1)$$

$$R_{tj} = \frac{1}{\frac{1}{R_{t(j-1)} + R_{3j-2}} + \frac{1}{R_{3j-1}} + \frac{1}{R_{3j}}} \quad (j \ge 2) \qquad (22)$$

$$R = R_{tj} + R_{3j+1}$$

According to equations 22 obtain the seepage resistance of complex fracture networks, and then use equations 23 to obtain the permeability of complex fracture networks.

$$K_{nw} = \frac{\mu L}{RwH} \qquad (23)$$

Where K_{nw} is the permeability of complex fracture network (μm^2).

$$F_{nwc} = K_{nw} w_f \qquad (24)$$

Where F_{nwc} is the conductivity of complex fracture network (μm^2.cm).

4.2 Verification of the fracture network conductivity model

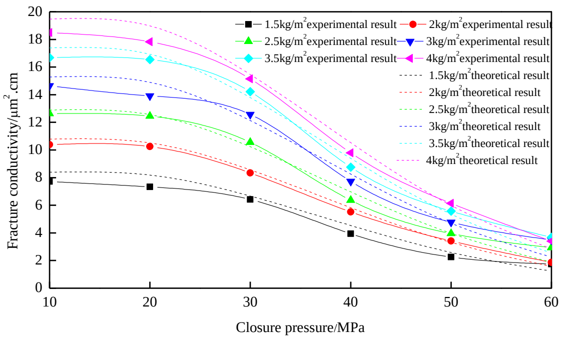

Fig 20. Comparison between theoretical and experimental results under different concentrations of "T" type

Figure 20 demonstrates strong congruence between the analytical model-predicted fracture network conductivity and experimental measurements. The error for different sand concentrations is about 13%. When the closure pressure is less than 50 MPa, the

root mean square error is 5.82%. Under high closing pressure, the error is relatively large, but the difference is small, and the error is within a reasonable range.

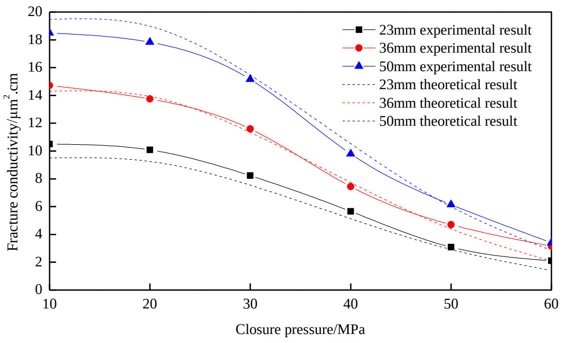

Fig 21. Comparison between theoretical and experimental results different fracture heights of "T" type

As shown in Fig 21, the theoretical calculation results still remain in good agreement with the experimental data curves under different fracture heights. The error between theoretical and experimental results across different fracture heights is 13.16%, indicating high accuracy. When the closure pressure is below 50 MPa, the theoretical and experimental results exhibit excellent consistency with minimal deviation, with an error of 5.53%.

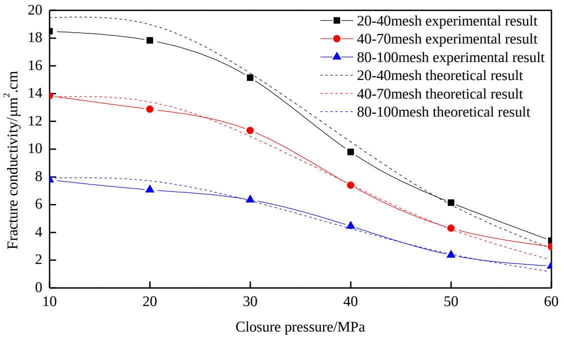

Fig 22. Comparison between theoretical and experimental results different proppant diameters of "T" type

From Fig 22, the root mean square error for different proppant diameters is 11.27%, which meets engineering accuracy requirements. Under low closure pressure conditions, the root mean square error is 3.96%, demonstrating high precision.

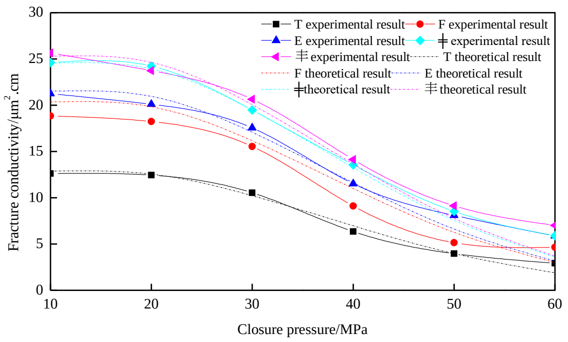

Fig 23. Comparison between theoretical and experimental results under different fracture structures

As shown in Figure 23, under identical conditions but different fracture network structures, the theoretical model results exhibit strong agreement with the experimental data. The root mean square error is 9.81%, with the primary source of error attributed to lower measurement accuracy under high closure pressure (60 MPa). At low closure pressures, the root mean square error decreases to 8.20%.

The new model established in this study demonstrates high accuracy, with a root mean square error below 14%, meeting the requirements for engineering applications. The theoretical results show excellent agreement with experimental data when the closure pressure is below 50 MPa. However, the accuracy decreases under high closure pressures, primarily due to limitations in the precision and reliability of experimental measurements under such conditions. Additionally, the new model assumes that all capillary flow pathways are horizontally oriented, whereas in reality, both horizontal and vertical capillaries exist within the proppant. At low pressure, gas predominantly flows along horizontal directions with minimal vertical movement. Under high closure pressures, proppant crushing increases gas flow through vertical capillaries, causing the model to underestimate experimental results and resulting in larger deviations.

4.3 Application of the fracture network conductivity model

The fracture conductivity of different numbers of secondary fractures was calculated based on the new model.

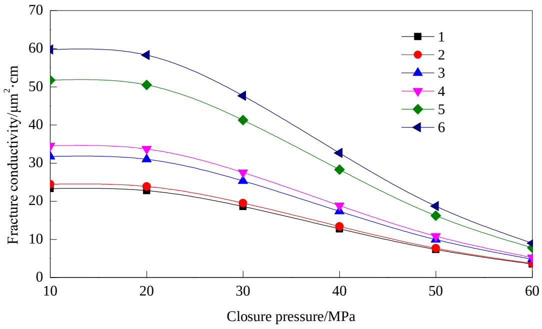

Fig 24. Conductivity of fracture network under different numbers of secondary fractures

From Fig 24, the more secondary fractures, the greater the conductivity of the fracture network. The increase of conductivity caused by the increase in the number of secondary fractures were 5%, 29.63%, 8.79%, 49.89%, and 15.45%, respectively. The more secondary fractures there are, the more seepage channels in the matrix increase, and the seepage resistance decreases.

The aspect ratio is defined as the ratio of the main fracture length to the secondary fracture length. This parameter governs the geometry of the fracture network and serves as a critical parameter in hydraulic fracturing design. In this study, aspect ratios of 2, 3, 4, 5, and 6 were investigated.

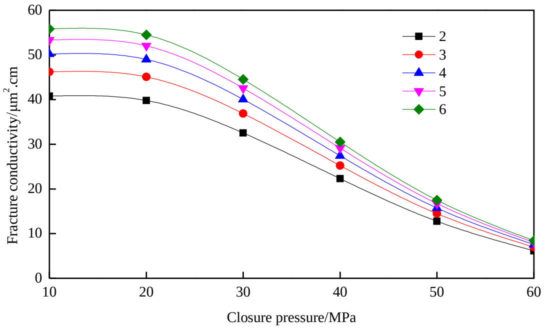

Fig.25 Conductivity of fracture network under different aspect ratio

As shown in Figure 25, the conductivity of fracture network increases with the aspect ratio. However, the rate of increase diminishes as the aspect ratio rises. At a closure pressure of 20 MPa, when the aspect ratio increases from 2 to 3, the conductivity rises from 39.82 μm²·cm to 45.10 μm²·cm, representing a 13.26% improvement. In contrast, when the aspect ratio increases from 5 to 6, the conductivity only improves from 52.04 μm²·cm to 54.49 μm²·cm, corresponding to a

smaller gain of 4.61%. These results indicate that while higher aspect ratios enhance conductivity, the incremental gain becomes progressively smaller. For practical fracture network design, it is recommended to select an aspect ratio between 4 and 5.

Conclusions

In this study, experiments are conducted to investigate the effects of critical influential factors on the conductivity of the fracture network. A new model of fracture network conductivity is established. The following conclusion can be drawn:

1. The experiment showed that as closure pressure increases, the conductivity of the fracture network decreases. Quartz sand proppants show a similar trend, but the conductivities are lower compared with ceramics.
2. The higher the sand concentration, the greater the conductivity. As the fracture height and proppant diameter increase, the conductivity increases. The closer the position of the secondary fracture is to the outlet, the greater the increase in the conductivity of the fracture network.
3. The orders of the conductivity is as follows: "-" type < "+"-1 type < "T" type < "+"-2 type < "F" type < "E" type < "+"-3 type < "╪" type < "Ɨ" type. Orthogonal experimental analysis shows that closure pressure and the position of secondary fractures significantly impact the conductivity of the fracture network. The proppant diameter, and sand concentration also have a greater impact, while the number of secondary fractures, type of proppant and fracture height have a weaker impact.
4. An analytical model of single fracture conductivity was established based on the Kozeny-Carman equation and experimental data. Based on the principle of hydroelectric similarity, a new model of the fracture network conductivity was ultimately established.
5. For fracture network fracturing optimization, it is recommended to use smaller proppant particle sizes (40-70 mesh) with lower proppant concentration and high-strength proppant. In fracture network design, an aspect ratio of 4~5 is

preferred, and multiple secondary fractures should be designed near the wellbore to enhance conductivity.

Conflicts of interest

The authors declare on conflict of interest.

Funding

This work was supported by the Natural Science Foundation of Sichuan Province under Grant 2023NSFSC0945. The funder had no role in study design, data collection and analysis, decision to publish, or preparation of the manuscript.

Data Availability Statement

All relevant data are within the paper and its Supporting Information files.

References

1. Lu S, Huang W, Chen F, Li J, Wang M, Xue H, et al. Classification and evaluation criteria of shale oil and gas resources: Discussion and application. Petroleum Exploration and Development. 2012; 39(2): 268-276.
2. Nobakht M, Clarkson CR. Analysis of production data in shale gas reservoirs: Rigorous corrections for fluid and flow properties. Journal of Natural Gas Science and Engineering. 2012; 8: 85-98.
3. Hui G, Chen Z, Schultz R, Chen S, Song Z, Zhang Z, et al. Intricate unconventional fracture networks provide fluid diffusion pathways to reactivate pre-existing faults in unconventional reservoirs. Energy. 2023; 282: 128803.
4. Chen M, Geng J, Zhao L. A hybrid numerical model for coupled hydro-mechanical analysis during CO2 injection into heterogeneous unconventional reservoirs. Gas Science and Engineering. 2024; 124: 205-244.
5. Zhao S, Zhao Q, Zhang X, Jiang X, Wu H, Yan J. Key policy needs for the success of China's shale gas revolution. Energy Reports. 2024; 11(40): 15-20.
6. Alhemdi A, Gu M. Method to account for natural fracture induced elastic anisotropy in geomechanical characterization of shale gas reservoirs. Journal of Natural Gas Science and Engineering. 2022;101:104478.

7. Zhao Y, Yang H, Wu J, Liu C, Chang C, Yu W, et al. Choke management simulation for shale gas reservoirs with complex natural fractures using EDFM. Journal of Natural Gas Science and Engineering. 2022;107:104801.
8. Zhao Y, Xiao-Tao W, Li C-L, Liu Y, Xie C-L. Systematic prediction of the gas content, fractures, and brittleness in fractured shale reservoirs with TTI medium. Petroleum Science. 2024.
9. Cipolla CL, Warpinski NR, Mayerhofer MJ, Lolon EP, Vincent MC, editors. The Relationship Between Fracture Complexity, Reservoir Properties, and Fracture Treatment Design. SPE Annual Technical Conference and Exhibition; 2008. SPE-115769-MS.
10. Ebrahimi AN, Wittel FK, Araújo NAM, Herrmann HJ. Multi-scale approach to invasion percolation of rock fracture networks. Journal of Hydrology. 2014; 519: 353-63.
11. Liu Y, Xiu N, Ding Y, Wang X, Lu Y, Dou J, et al. Analysis of multi-factor coupling effect on hydraulic fracture network in shale reservoirs. Natural Gas Industry. 2015; 2(2): 162-166.
12. Ren L, Wang Z, Zhao J, Lin R, Wu J, Song Y, et al. Shale gas effective fracture network volume prediction and analysis based on flow back data: A case study of southern Sichuan Basin shale. Geoenergy Science and Engineering. 2023; 228: 211963.
13. Matthäi SK, Bui CM, Hansika H, Perera MSA. Influence of inertial and centrifugal forces on rate and flow patterns in natural fracture networks. Advances in Water Resources. 2024: 104741.
14. Liu X. Shale-gas well test analysis and evaluation after hydraulic fracturing by stimulated reservoir volume (SRV). Natural Gas Industry . 2016; 3(6): 577-584.
15. Zhang Q, Su Y, Wang W, Lu M, Ren L. Performance analysis of fractured wells with elliptical SRV in shale reservoirs. Journal of Natural Gas Science and Engineering. 2017; 45: 380-390.
16. Jiang R, Zhang F, Cui Y, Qiao X, Zhang C. Production performance analysis of fractured vertical wells with SRV in triple media gas reservoirs using elliptical flow. Journal of Natural Gas Science and Engineering. 2019; 68: 102925.
17. Liu X, Jin Y, Lin B. Classification and evaluation for stimulated reservoir volume (SRV) estimation models using microseismic events based on three typical grid structures. Journal of Petroleum Science and Engineering. 2022; 211: 110-169.
18. Wen Q, Zhang S, Wang L, Liu Y. Influenceof proppant embedment on fracture long-term flow conductivity. Natural Gas Industry. 2005; 25(05): 65-8+9-10.
19. Lu Q, He Y, Xu W, Tian R, Zhang F. Volume Fracturing Fracture Network Configuration's

Influence on Fracture Diverting Capacity. Journal of Changzhou University (Natural Science Edition). 2014; 26(02): 70-74..

20. Wen Q, Wang S, Duan X, Li Y, Wang F, Jin X. Experimental investigation of proppant settling in complex hydraulic-natural fracture system in shale reservoirs. Journal of Natural Gas Science and Engineering. 2016;33:70-80.
21. Liang X, Zhou F, Liang T, Huang Y, Wei D, Ma S. The effect of combined proppants upon the fracture conductivity in tight gas reservoirs. Energy Reports. 2020; 6: 879-884.
22. Wang Z, Lin B, Chen G, Dai Y, Chen A. Experimental study on evaluation of conglomerate reservoir support-type fracture conductivity in Xinjiang oilfield. Energy. 2023; 278: 127877.
23. Shi X, Ge X, Zhang Y, Huang W, Zhan Y, Gu Y, et al. Experimental study of propped fracture conductivity in tight dolomite reservoir. Petroleum Geology and Recovery Efficiency. 2023; 30(04): 167-172.
24. Katende A, Allen C, Rutqvist J, Nakagawa S, Radonjic M. Experimental and numerical investigation of proppant embedment and conductivity reduction within a fracture in the Caney Shale, Southern Oklahoma, USA. Fuel. 2023; 341: 127571.
25. Liu K, He H, Yin D. Theoretical and experimental study on the long-term conductivity of heterogeneous artificial fractures in tight reservoirs. Energy Reports. 2023; 9: 3881-3895.
26. Li K, Gao Y, Lyu Y, Wang M. New Mathematical Models for Calculating Proppant Embedment and Fracture Conductivity. SPE Journal. 2015; 20(03): 496-507.
27. Liu Z, Zhao H, Wang D, Yuan P, He Y. Establishment and application of propped hydraulic fracture conductivity theoretical model based on fracturing efficiency index. Gas Science and Engineering. 2024; 121: 199-205.
28. Liu Y, Wu L, Guo J, He S, Wu Y. Model for fracture conductivity considering particle size redistribution caused by proppant crushing. Geoenergy Science and Engineering. 2024; 240: 213081.
29. Nwabia FN, Leung JY. A Practical probabilistic history matching framework for characterizing fracture network parameters of shale gas reservoirs. Gas Science and Engineering. 2024; 126: 305-26.
30. Sun Z, Wang H, Zhang B, Li Y, Peng Z, Zhang S. Intelligent construction method and application of large-scale three-dimensional complex discrete fracture network model based on particle

swarm optimization algorithm. Computers and Geotechnics. 2024; 171: 306-16.

31. Tan Y, Pan Z, Liu J, Feng X-T, Connell LD. Laboratory study of proppant on shale fracture permeability and compressibility. Fuel. 2018;222:83-97

32. Wu D, Li S, Zhang N, Guo Y, Liu L, Wang Z. A novel permeability model for hydrate-bearing sediments integrating pore morphology evolution based on modified Kozeny-Carman equation. Energy. 2023; 277: 127723.

33. Xiao B, Zhu H, Chen F, Long G, Li Y. A fractal analytical model for Kozeny-Carman constant and permeability of roughened porous media composed of particles and converging-diverging capillaries. Powder Technology. 2023; 420: 118256.

Click here to access/download
Supporting Information
original data.xlsx

Experimental and theoretical investigation on the conductivity of complex fracture network in unconventional gas reservoirs

Jinjian Gao[1*], Lanxiao Hu[2]

1 Airport Department, Jiangxi Flight University, Nanchang, Jiangxi, China

2 College of Energy Resources, Chengdu University of Technology, Chengdu, Sichuan, China

*Corresponding author: Jinjian Gao

E-mail: gaojinjian1990@163.com

These authors contributed equally to this work.

Abstract

With the depletion of conventional oil and gas resources, unconventional oil and gas resources have become crucial as alternative hydrocarbon sources. Unconventional gas reservoirs typically have low porosity and permeability, requiring reservoir stimulation to create complex fracture network. Unlike single fracture, the fracture network exhibit complicated structures due to multiple fractures and their various distribution manners. To study the conductivity of complex fracture network, a new conductivity chamber is designed based on a discretized fracture network model. Experiments and orthogonal analysis are conducted to investigate the effect of critical influential factors such as fracture network structure, fracture height, proppant concentration, proppant diameter, and proppant type on the conductivity of complex fracture network. The results show that compared to a single fracture, the T-shape, F-shape, E-shape, ╪-shape, Ɨ –shape fracture networks conductivity increase by 49%, 131%, 150%, 188%, 201%, respectively. Closure pressure and secondary fracture distribution significantly impact the conductivity of complex fracture network. Proppant diameter and proppant concentration also have substantial effects. Compared with existing experimental studies focusing on single fracture conductivity, this study presents experimental research on the conductivity of complex fracture network, thereby addressing a research gap in this field. Through synergistic integration of the Kozeny-Carman hydrodynamic theory with hydraulic-electrical analogy principle, an innovative model of fracture network conductivity is formulated. The advantage of new model of fracture network conductivity is that it combines a theoretical method and an experimental method. This study provides valuable insights for optimizing volume fracturing in tight reservoirs, facilitating their rapid and efficient development.

Keywords: Complex Fracture Network; New Conductivity Chamber; Fracture Conductivity; Secondary Fracture; Closure Pressure; New Model of Fracture Network Conductivity

1 Introduction

Unconventional gas reservoirs generally have no natural productivity without fracturing. Hydraulic fracturing has become one of the key technologies in the development of unconventional gas reservoirs, enabling them to achieve industrial gas flows and making the development of unconventional gas reservoirs feasible [1-5]. Typically, the fractures formed in conventional reservoirs are single, symmetrical, bi-wing fractures. However, unconventional gas reservoirs have many natural fractures and possess high brittleness [6-8], which tend to create complex fracture network rather than the single fracture. These observations have been extensively found in the North American hydraulic fracturing practices [9-13]. During the fracturing process, brittle rocks and natural fractures continuously open or shear, forming a complex network of artificial and natural fractures. This approach leads to a substantial enhancement of the stimulated reservoir volume [14-17]. The technique to enhance production and ultimate recovery by creating such complex fracture network is known as volume fracturing, which plays an increasingly important role in the development of unconventional oil and gas reservoirs.

As is well known, fracture conductivity is a key parameter for volume fracturing. In recent years, experiments of fracture conductivity have evolved from single proppant types and sizes to multiple types and mixed sizes. Wen et al. [18] pioneered the use of the long-term fracture conductivity instrument in China to study proppant embedding by using formation cores. However, research object was single fracture. Lu et al. [19] simulated fracture networks using various grooves with rectangular cross-sections. They examined the conductivity of five different network structures: "一", "T", "L", "F", and "╪". It proves that the fracture network created by volume fracturing have different influence on fracture conductivity. This experiment ignored crushing and embedment of proppant. Wen et al. [20] designed a complex fracture network simulation device to study proppant settlement in "一", "T", "H", and "TF" network configurations based on discretized fracture network model. This provides a foundation for the experiment in this article. Liang et al. [21] evaluated the fracture conductivity with various ratios of sands to ceramic proppants by use of standard Fracture Conductivity Evaluation System under different effective closure stresses.

Wang et al. [22] conducted triaxial experiments to explore the effects of displacement, stress difference, and weak surfaces. Shi et al. [23] examined the fracture conductivity of tight dolomite reservoirs, analyzing various influencing factors. They found that proppant diameter and sand concentration significantly impacted fracture conductivity. Katende et al. [24] employed experimental and analytical simulation methods to study proppant embedment and fracture conductivity within Caney Shale fractures. Improved fracture conductivity is attributed to Proppant diameter. Liu et al. [25] conducted experiments on long-term non-uniform sand spreading in hydraulic fractures of tight reservoirs, considering different influencing factors. Based on the conductivity loss mechanism, the calculation model of the long-term conductivity of heterogeneous artificial fractures is established.

In order to guide hydraulic fracturing field, the single hydraulic fracture conductivity model has been established. There are two types of models, one is an empirical model based on experimental results, and the other is a theoretical model derived from fluid mechanics in porous medium and capillary model. Li et al. [26] created a new mathematical model to calculate fracture conductivity and proppant embedment. Moreover, factors affecting the fracture conductivity have been analyzed including proppant diameter, closure pressure, and proppant type. Liu et al. [27] proposed an innovative fracture conductivity calculation model based on a "fracturing efficiency index". Liu et al. [28] established a new model for predicting the fracture conductivity considering the particle size redistribution caused by proppant crushing based on Kozeny–Carman equation. However, the most important point has been overlooked: the fracture network is composed of multiple fractures.

In previous research, experimental research on fracture conductivity has mainly focused on single fracture. The experimental instrument for fracture conductivity is still traditional diversion chamber which cannot meet the requirements of testing the conductivity of complex fracture network. There is very little research on the calculation model of the conductivity of fracture network.

Firstly, a new diversion chamber was designed and manufactured based on a discrete fracture network model. Then, rock slab is combined into fracture network.

Experiments with different fracture structures will be conducted using a new type of diversion chamber. Orthogonal experiments were used to study the primary and secondary factors affecting the conductivity of complex fracture network. Finally, based on the Kozeny-Carman equation and the principle of hydraulic-electrical analogy, a new model of fracture network conductivity is established.

2 Experimental methods

Traditional conductivity measurement chambers can only test the conductivity of a single fracture, falling short of testing the conductivity of complex fracture network. In this study, we propose a new conductivity measurement chamber based on the experimental observations obtained from the settlement and transportation of proppant in the complex fracture networks and discrete fracture networks [29, 30]. This new chamber features a larger inner cavity space enabling the investigation of fracture networks and can be integrated with the fracture conductivity measurement setup.

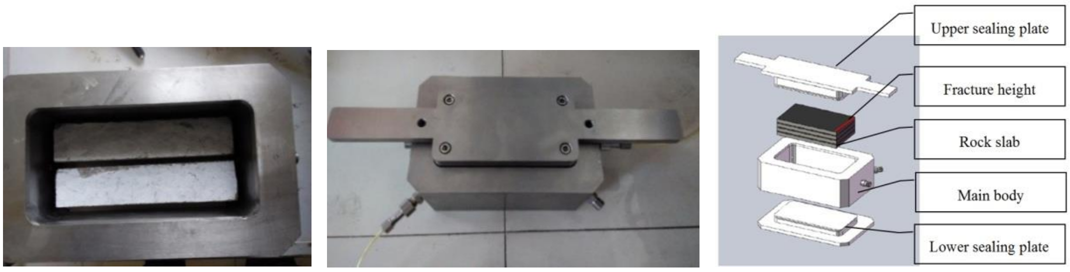

(a) Internal diagram **(b) External image** **(c) Explosion view**

Fig 1. Structure diagram of new conductivity measurement chamber

Fig 1 illustrates the internal structure of the new conductivity measurement chamber with a dimension of 170mm×90mm×50mm. The chamber is equipped with stainless steel pads to adjust the fracture height. The compressive strength of stainless steel is 520 MPa. Various shapes of rock slabs simulate different fracture networks, the surrounding space is sealed with foam rubber. There are four interfaces to monitor the inlet and outlet pressures and flow rates. Compared with the standard conductivity measurement chamber, the new proposed chamber possesses the following features:

(1) The diversion chamber, constructed from stainless steel, undergoes wire cutting internally to ensure smooth cavity walls.

(2) The rock slab is positioned 10mm from the edge wall on one side. This arrangement ensures even gas distribution at the fracture's inlet and outlet, mitigating the end-effect and enhancing test accuracy.

(3) Due to the application of sealant on both sides of the rock slab, pressure measurement on the walls is not feasible. Pressure measuring ports are located on the inlet and outlet side walls to obtain the pressure differences. The effective fracture length is 160mm.

(4) Filter plugs are installed in the gas inlet and pressure measuring ports to prevent the leakage of the crushed proppant during the experimental process.

(5) The maximum closure pressure is 100 MPa.

The experimental instrument primarily comprises six components, as illustrated in Fig 2.

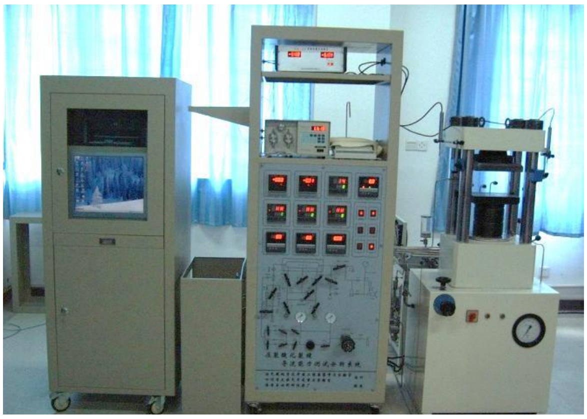

Fig 2. Fracture conductivity instrument of FCES-100

This experiment uses the volume equivalence principle, where the void volume in the fracture network equates to an imaginary single fracture's volume. The fracture network width is assumed to be the same as the width of the imaginary single fracture. This width is calculated by dividing the total volume by the surface area parallel to the seepage direction:

$$w_f = V / S \tag{1}$$

Where w_f is equivalent fracture width (cm), V is proppant volume (cm^3), S is horizontal area of fracture network (cm^2).

Calculation equation for fracture conductivity can be written as:

$$kw_f = \frac{Q\mu L w_f}{A_f \Delta P} \quad (2)$$

Where k is permeability (μm^2), Q is flow rate(cm^3/s), μ is gas viscosity (mPa·s), L is fracture conductivity length (cm), A_f is cross section area of fracture network (cm^2), ΔP is pressure difference between inlet and outlet (0.1MPa).

We construct several typical fracture network structures based on the discretized fracture network model. Fig 3 summarizes these structures, including "—" type, "T" type, "F" type, "E" type, "+" type, "╪" type, and "Ɨ" type. For the conductivity measurements, it is assumed that the width of the secondary fracture is equal to that of the main fracture.

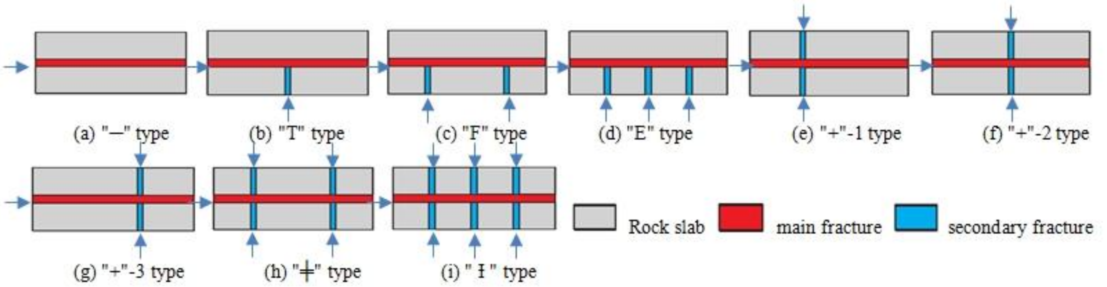

Fig 3. Schematics of different types of fracture networks

Taking the "—" type fracture network as an example, the experimental scheme for changing the sand concentration is as follows: the fracture height is 50mm, and the sand concentrations are 1.5, 2, 2.5, 3, 3.5, and 4 kg/m². Because unconventional reservoirs commonly employ low sand ratios and small-sized proppants during fracturing operations [31]. The proppant is 20-40 mesh ceramic proppant. The experimental scheme for changing the fracture height is as follows: the fracture heights are 23, 36 and 50 mm, with a sand concentration of 4 kg/m². The rock slabs provided by the laboratory can only be processed into sizes of 23, 36, and 50mm. The length-to-height ratio of fractures was designed based on field observations and theoretical frameworks widely adopted in unconventional reservoir engineering. Specifically, we focused on a range of 0.14 to 0.35 for this ratio, which aligns with typical fracture geometries observed in shale and tight sandstone reservoirs under similar stress conditions. The proppant used is 20-40 mesh ceramic proppant. The experimental scheme for changing the proppant

diameter is as follows: the fracture height is 50 mm, the sand concentration is 4 kg/m², and the proppants are 20-40, 40-70, and 80-100 mesh ceramic proppants. For the experimental scheme involving different types of proppants, only the proppant type is changed to ceramsite and quartz sand. The experimental scheme for other fracture structures is similar to that of the "—" type fracture.

Experimental procedure: Cut the rock slab and combine it into a fracture network. Place a specific amount of proppant in the fractures. Seal the gap and unpropped fracture with sealant, and install the sealing plate in the measurement chamber. Connect the inlet and outlet pressure and flow ports to the chamber. Pressurize the diversion chamber with closure pressures of 10, 20, 30, 40, 50, and 60MPa.

3 Results and discussion

3.1 Experimental results of "—type" fracture network

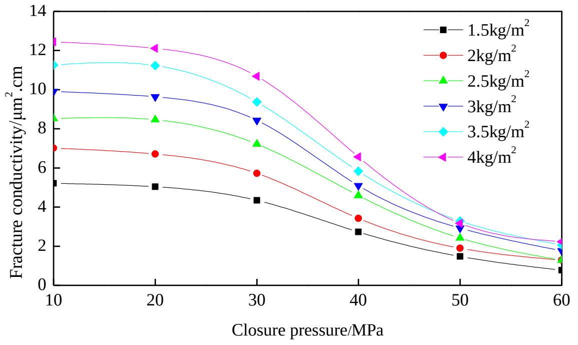

Fig 4. Fracture conductivity of "—" fracture network with different ceramic concentrations

Fig 4 shows the conductivity of fracture network under different closure pressures and proppant concentrations. The trend of the fracture network's conductivity with closure pressure is similar across different proppant concentrations. As closure pressure increases, the conductivity of the fracture network decreases. When the proppant concentration is 4 kg/m², the conductivity is 12.446 μm²·cm at 10 MPa, decreases to 10.681 μm²·cm at 30 MPa, and drops to 2.228 μm²·cm at 60 MPa. Based on the analysis of six sets of curves, the decrease in conductivity under high closure pressure (60MPa) is between 81.7% and 85.1% compared to low closure pressures (10MPa). At low closure pressure, the proppant stays intact. The large pores formed by the proppant results in high permeability and greater fracture conductivity. The decrease in

conductivity is due to the elastic deformation of the proppant, which slightly reduces pore size. At high closure pressure, the proppant breaks. The broken ceramic proppant has low porosity, resulting in lower permeability and significantly reduced fracture conductivity. In this experiment, rock slabs with high brittleness and high Young's modulus were used. There was less proppant embedment, and the fracture conductivity was greater than that of rock slabs with low Young's modulus. The closure pressure is generally the minimum principal stress. The smaller the minimum stress is, the greater the conductivity capacity will be.

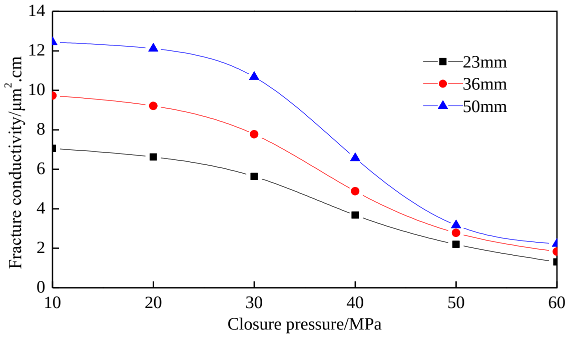

Fig 5. Fracture conductivity of "—" fracture network with different fracture heights

From Fig 5, it can be observed that an increase in closure pressure leads to a decrease in fracture conductivity. The conductivity of the fracture network increases with fracture height. The fracture conductivity increases exponentially with fracture height. To increase fracture height in the deep part of the fracture, it's necessary to use high displacement and high viscosity fracturing fluid to carry the proppant, forming a high and long propped fracture to improve the conductivity.

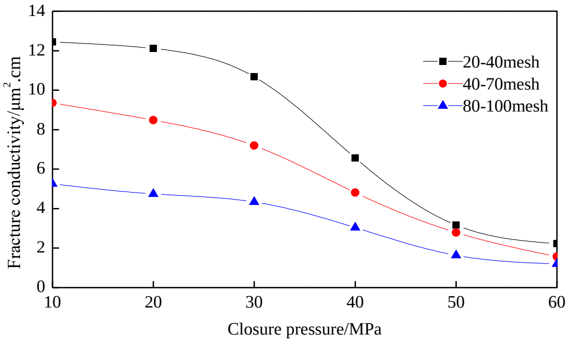

Fig 6. Fracture conductivity of "—" fracture network with different proppant diameters

Fig 6 shows that the conductivity of fracture network increases with the proppant diameter. The 20-40 mesh proppant has the highest conductivity, while the 80-100

mesh proppant has the lowest due to its smaller particle diameter. The difference in conductivity among the three proppant diameters is significant under low-pressure conditions but less notable under high-pressure conditions. Due to the complex structure and various fracture widths of fracture network, larger proppant cannot easily enter the small fractures. Based on the experimental results, the optimal Proppant diameter for volume fracturing is 40-70 mesh, which has a smaller decrease in conductivity compared to the 20-40 mesh proppant and can be carried into the narrows fractures.

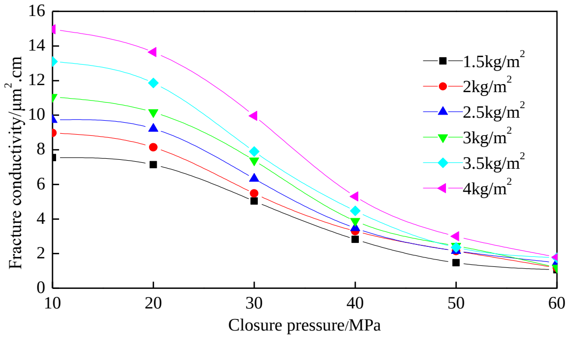

Fig 7. Fracture conductivity of "—" fracture network with different quartz sand concentrations

Comparing Fig 4 and Fig 7, the fracture conductivity of quartz sand at 10 MPa is slightly higher than that of ceramic proppant. However, under high pressure, it is lower than that of ceramic proppant. The fracture conductivity decreases by 92.1% with the closure pressure, indicating that the quartz sand possess lower conductivity. It can be concluded that when the closure pressure is greater than 30 MPa, quartz sand cannot effectively provide higher conductivity and is unsuitable for high closure pressure conditions.

3.2 The influence of fracture network structure on conductivity

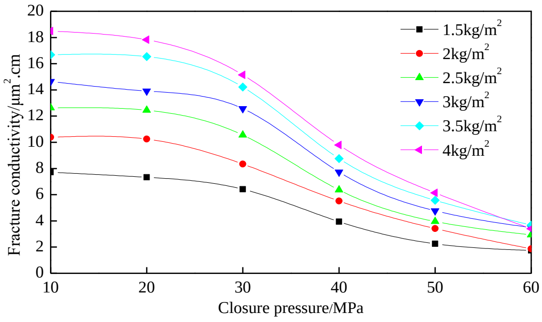

(a) Concentration of ceramic proppant

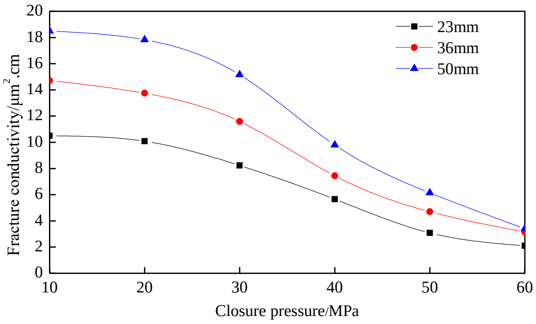

(b) Fracture height

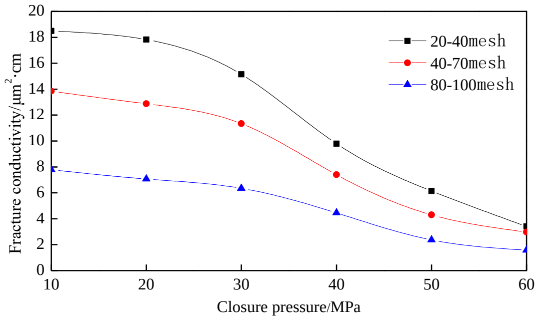

(c) Proppant diameter

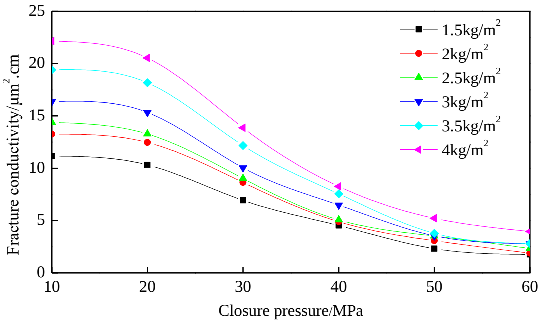

(d) Concentration of quartz sand

Fig 8. Experimental results of "T" fracture network

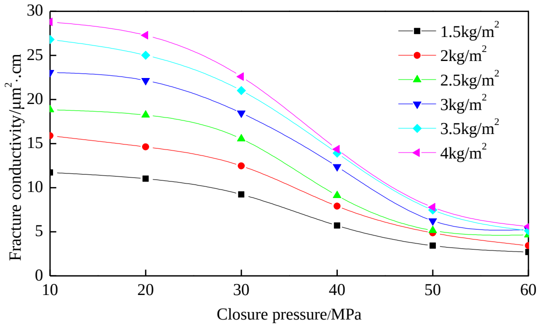

(a) Concentration of ceramic proppant

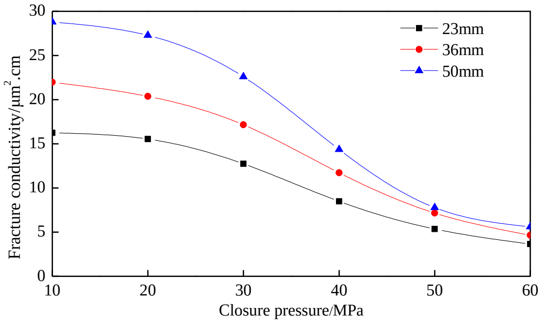

(b) Fracture height

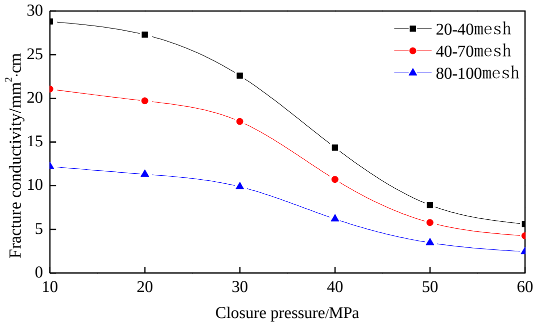

(c) Proppant diameter

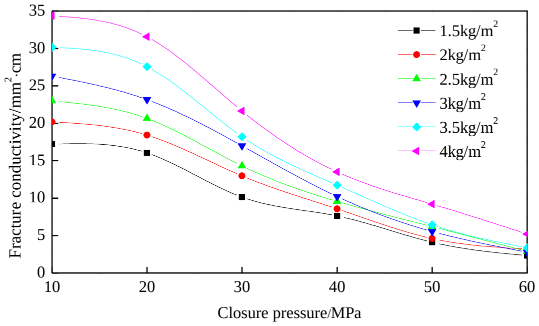

(d) Concentration of quartz sand

Fig 9. Experimental results of "F" fracture network

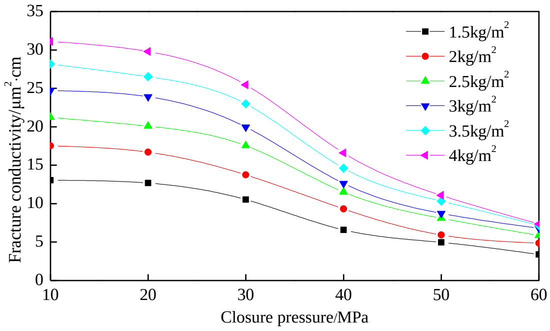

(a) Concentration of ceramic proppant

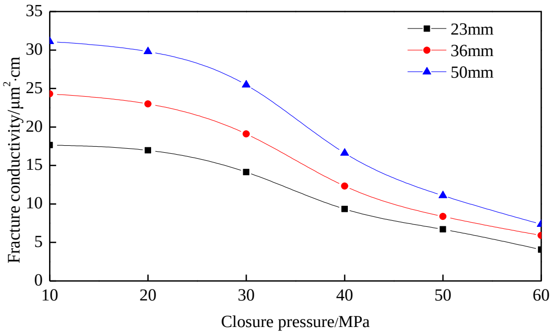

(b) Fracture height

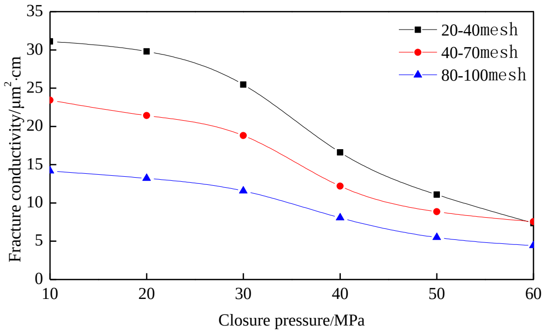

(c) Proppant diameter

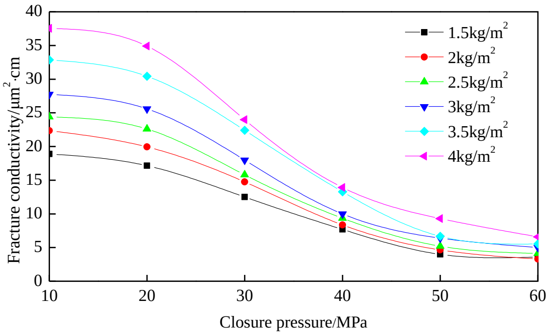

(d) Concentration of quartz sand

Fig 10. Experimental results of "E" fracture network

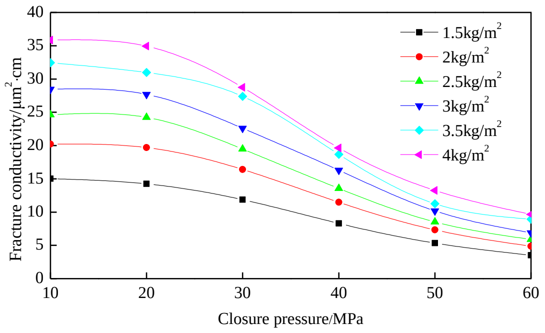

(a) Concentration of ceramic proppant

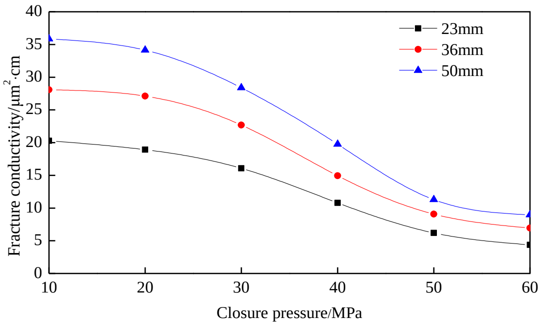

(b) Fracture height

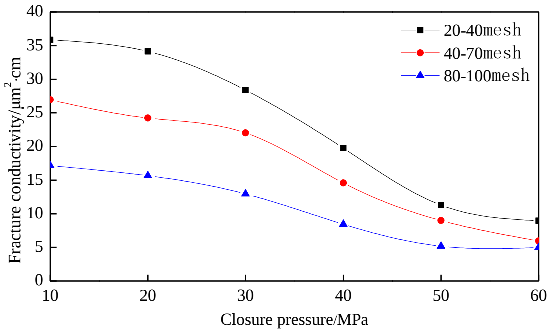

(c) Proppant diameter

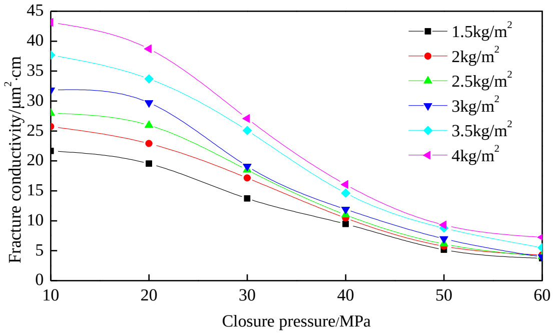

(d) Concentration of quartz sand

Fig 11. Experimental results of "╪" fracture network

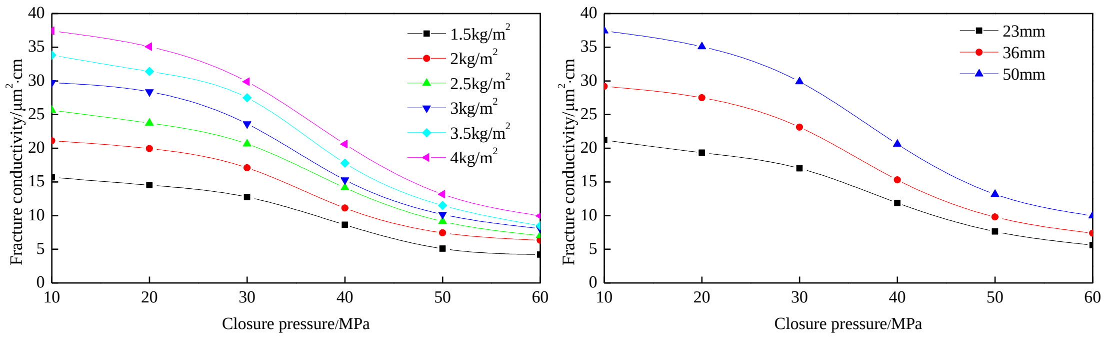

(a) Concentration of ceramic proppant **(b) Fracture height**

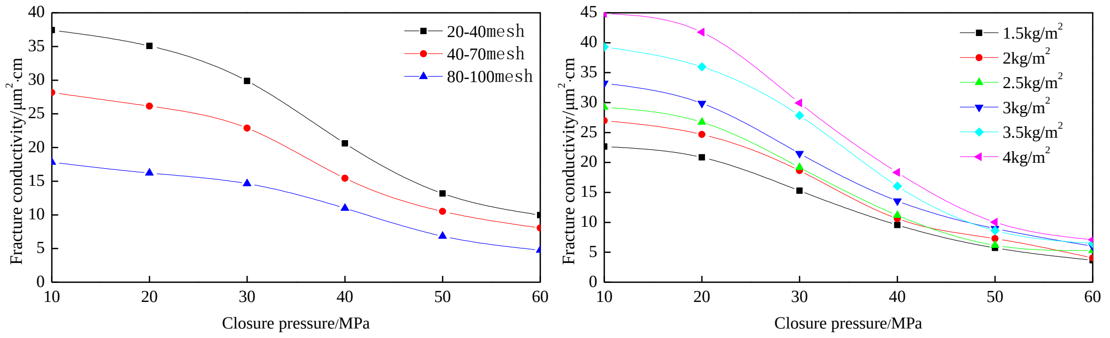

(c) Proppant diameter **(d) Concentration of quartz sand**

Fig 12. Experimental results of "Ɨ" fracture network

Fig 8-12 shows the conductivity of different fracture networks under different conditions.

The conductivity of different types of fracture networks are analyzed under the same conditions.

Table 1. Influence of secondary fracture number on fracture conductivity

Fracture network	"T" type	"F" type	"E" type	"╪" type	" Ɨ" type
Number of secondary fractures	1	2	3	4	6
Initial fracture conductivity growth factor	1.49	2.31	2.50	2.88	3.01
Final conductivity /Initial conductivity	18.4%	19.5%	23.7%	26.9%	29.3%

From Table 1, it can be seen that compared to the "─ type" fracture network, the initial fracture conductivity continuously increases with the number of secondary fractures. The growth factors of the fracture network conductivity are 1.49, 2.31, 2.50, 2.88, and 3.01 for "T", "F", "E", "╪", and "Ɨ" types, respectively. The ratio of the final conductivity (60MPa) to the initial conductivity (10MPa) also indicates that more secondary fractures lead to greater final fracture conductivity. Sufficient secondary fractures under high pressure can ensure that the formation maintains a certain degree of diversion capacity.

Table 2. Influence of secondary fracture position on fracture conductivity

Fracture network	“+”-1 type	“+”-2 type	“+”-3 type
Distance from air outlet /mm	120	80	40
Initial fracture conductivity growth factor	1.20	1.66	2.82
Final conductivity /Initial conductivity	20.6%	22.1%	24.9%

From Table 2, it can be evidently observed that the nearer the secondary fractures are to the outlet of the chamber, the more significant the increase in the conductivity of the fracture network. From the ratio of the final conductivity (60MPa) to the initial conductivity (10MPa), it can be observed that the closer the secondary fractures are to the outlet, the greater the conductivity of the fracture network under high pressure. The closer the secondary fracture is to the outlet, the smaller the resistance during the flow process and the lower the energy loss in the secondary fractures. When the fluid enters the main fracture, the closer it is to the outlet, the lower the energy of the fluid in the main fracture, which means that the energy loss of mixing the fluid in the main and secondary fractures is the smallest and the increase in fracture conductivity is the largest.

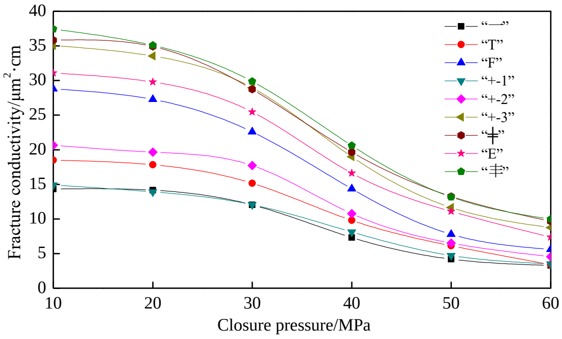

Fig 13. Comparison of the conductivity of nine types of fracture network

Using the 20-40 mesh ceramic proppant, with a sand concentration of 4 kg/m² and a fracture height of 50 mm, conductivity obtained from nine types of fracture network were plotted in Fig 13. The order of conductivity is as follows: "—" type < "+"-1 type < "T" type < "+"-2 type < "F" type < "E" type < "+"-3 type < "╪" type < "丰" type. The following observations have been found: (1) The conductivity of the "—" and "+"-1 fracture networks are close to each other and exhibit lowest conductivity values. Although the "+"-1 fracture network has two secondary fractures, their locations are far away from the outlet, resulting in high pressure consumption during flow and

significant energy loss when the fluid converges from the secondary fractures into the main fracture. (2) The conductivity of the "+"-2 fracture network is slightly higher than that of the "T" fracture network. The secondary fracture positions are the same, but the "+"-2 fracture network has an additional secondary fracture. (3) The conductivity of the "F" and "E" fracture networks is similar and significantly improved. Both have one secondary fracture closest to the outlet, indicating that the position of the secondary fracture significantly impacts the conductivity. (4) The conductivity of the "+"-3, "╪", and "Ɨ" types of fracture networks is the highest because they have two secondary fractures closest to the outlet. The "Ɨ" type has 6 secondary fractures, the "╪" type has 4, and the "+"-3 type has 2. Therefore, the "Ɨ" type has the highest conductivity, followed by the "╪" type, and the "+"-3 type has the lowest among the top three.

3.3 Orthogonal experimental analysis

This study considered seven influencing factors with the assumption of no interactions between them. Among these factors, proppant type was assigned two distinct levels, while the remaining six factors each had three levels. Through orthogonal array reference tables, the optimal experimental layout was identified as $L_{18}(2\times3^7)$. The designed orthogonal experiments evaluated outcomes using conductivity as the performance metric, with detailed results provided in Table 3.

Table 3. Results of the orthogonal experiments

Number	Proppant type	Number of secondary fractures	Distance from air outlet /mm	Fracture height /mm	Sand concentration /(kg/m^2)	Proppant diameter /mesh	Closure pressure /MPa	Error	Conductivity /μm^2·cm
1	Ceramsite	1	120	23	2	20-40	20	1	4.623
2		1	80	36	3	40-70	40	2	5.027
3		1	40	50	4	80-100	60	3	2.994
4		2	120	23	3	40-70	60	3	1.215
5		2	80	36	4	80-100	20	1	6.948
6		2	40	50	2	20-40	40	2	10.936
7		4	120	36	2	80-100	40	3	2.073
8		4	80	50	3	20-40	60	1	6.875
9		4	40	23	4	40-70	20	2	15.804
10	Quartz sand	1	120	50	4	40-70	40	1	6.305
11		1	80	23	2	80-100	60	2	1.147
12		1	40	36	3	20-40	20	3	17.339

13		2	120	36	4	20-40	60	2	1.674
14		2	80	50	2	40-70	20	3	6.624
15		2	40	23	3	80-100	40	1	3.402
16		4	120	50	3	80-100	20	2	6.378
17		4	80	23	4	20-40	40	3	8.627
18		4	40	36	2	40-70	60	1	2.774

The F critical value is selected according to the F value critical value table with a confidence interval of 0.01. According to the verification results in Table 4, the closure pressure and the position of secondary fractures have the greatest impact on the conductivity of the fracture network. The proppant diameter and the sand concentration also significantly affect conductivity, while the number of secondary fractures, proppant type and the fracture height exhibit a weaker impact.

Table 4. Analyses of the variances

Source of variance	Sum of squares	free degree	Mean sum of squares	F value	F critical value	Significance
Proppant type	0.275	1	0.275	0.088	3.46	
Number of secondary fractures	11.536	2	5.768	1.851	3.11	
Distance from air outlet	80.685	2	40.343	12.945	3.11	High saliency
Fracture height	2.631	2	1.315	0.422	3.11	
Sand concentration	19.490	2	9.745	3.127	3.11	Saliency
Proppant diameter	61.517	2	30.758	9.870	3.11	Saliency
Closure pressure	140.412	2	70.206	22.527	3.11	High saliency
Error	9.349	3	3.116			

Under high closure pressure, the proppant is crushed, leading to a significant reduction in fracture width and pore size, sharply decreasing permeability and conductivity. The structure of fracture network, including the number and position of secondary fractures, significantly impacts conductivity, especially the position of secondary fractures. Fracture conductivity increases as secondary fractures approach the outlet, with closer positioning minimizing fluid mixing-induced energy losses under equivalent fracture. Under comparable spatial configurations, an increase in secondary fracture number directly enhances fracture network conductivity. The flow diversion effect created by secondary fractures significantly mitigates gas flow energy dissipation within the fracture network, thereby enhancing the fracture conductivity. Larger sand concentration results in wider fracture width, which significantly enhances conductivity. Under the same conditions, higher fracture height increases conductivity,

but the impact is not as significant as the discussed five conditions. The influence of the type of proppant on fracture conductivity is the weakest.

4 Analytical investigation of fracture network conductivity

Based on experimental data of fracture network conductivity and discretized fracture network model, the sand layer inside the fracture is divided into two types: the sand layer on the fracture wall and the sand layer inside the fracture. Considering factors such as proppant embedding, fragmentation, and deformation, an analytical model of single fracture conductivity is first established. Leveraging the hydraulic-electrical analogy principle, a theoretical model for fracture network conductivity is rigorously derived through multiphysical coupling analysis. The accuracy analysis of the analytical model is carried out.

Certain simplified assumptions are necessary regarding the internal gas flow in the fracture network and the complex sand laying method of the proppant:

1. The shape of the fracture network is fish bone-like
2. Gas flow is Darcy flow;
3. Energy loss caused by gas collisions is neglected;
4. Secondary fractures are orthogonal to the main fracture;
5. The fluid flow in the rock matrix is neglected.

4.1 A new model of conductivity of fracture network

The basic assumptions of the analytical model for the conductivity of a single fracture are as follows: The proppant is a rigid sphere that does not deform or break; The proppant is arranged in a prismatic pattern. Based on the Kozeny-Carman model [32, 33], the proppant sand layer is treated as a capillary model (Fig 14). The new model assumes that all capillary are horizontal and uniform. In reality, both horizontal and vertical capillaries exist within the proppant. At low pressure, gas predominantly mainly flows along horizontal directions. Under high closure pressures, proppant crushing increases gas flow through vertical capillaries, leading to the decrease of the model's accuracy.

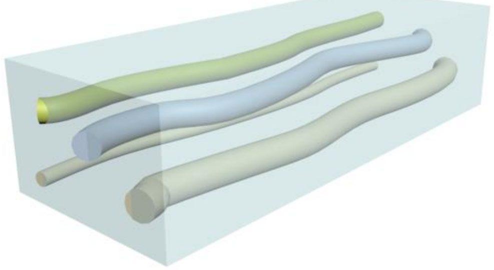

Fig 14. Schematic of the capillary porous rock physical model

The pores formed in the fracture can be divided into two types (Fig 15): one type is the pores near the fracture wall, which can be partially affected by the proppant embedding. The other type is the internal pores formed by the proppant, which is influenced by the Proppant diameter.

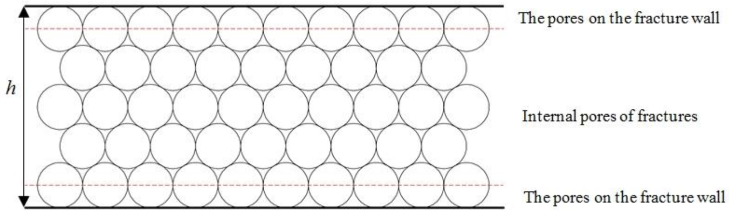

Fig 15. Schematic diagram of proppant bedding pores

(1) Calculation of the number of sand layers

$$h = c / \rho_p \tag{3}$$

Where c is proppant concentration (kg/m^2), ρ_p is bulk density of proppant (kg/m^3), h is proppant stacking height (m).

$$n = \left(\frac{c / \rho_p - 2r_p}{\sqrt{3} r_p} + 1 \right) \tag{4}$$

$$m = (n)_{\text{int}} \tag{5}$$

Where n is number of sand layers, r_p is proppant radius (m), m is the integer of n.

(2) Calculation of the proppant layers inside the fracture and the proppant layer near the fracture wall

Use N to represent the total number of proppants in the sand layer. The placement method of proppants is cumulative layer by layer, with the same number

placed in each layer

$$N=\left[n\left(\frac{H-2r_p}{\sqrt{3}r_p}+1\right)-A\right]\left(\frac{L}{2r_p}\right) \quad (6)$$

Where H is fracture height (m), L is fracture length (m), A is constant, N is total number of proppant placement (integer). $m=3i-2$, $A=2i-2$; $m=3i-1$, $A=2i-1$; $m=3i$, $A=2i$; m、 I、 A=1、2、3……

Due to the difference between the proppant layers adjacent to the fracture wall and other proppant layers, its layer number needs to be calculated.

$$N_f=\left[2\left(\frac{H-2r_p}{\sqrt{3}r_p}+1\right)-B\right]\left(\frac{L}{2r_p}\right) \quad (7)$$

Where N_f is the number of proppants adjacent to the fracture wall (integer), B is constant. $m=3i-2$, $B=0$; $m=3i-1$、 $m=3i$, $B=1$.

(3) Calculation of fracture width

The stacked proppant exhibits a triangular distribution under high closure pressure. The height of adjacent layers of proppant can be calculated by $2r_p\sqrt{6}/3$. Based on the fracture height, the fracture width can be evaluated by:

$$w=(n-1)2r_p\sqrt{6}/3+2r_p \quad (8)$$

Where w is fracture width (m).

(4) Porosity calculation

The porosity of the sand layer is the ratio of the pore volume of the sand layer to the total volume of the fractures supported by the proppant. According to the definition of porosity, the calculation equation for porosity is:

$$\phi=\frac{LHw-4N\pi r_p^{\,3}/3}{LHw} \quad (9)$$

Where ϕ is porosity of main and secondary fracture.

(5) Permeability calculation

Based on the assumption that the sand layer is viewed as a capillary model, the relationship between permeability and pore size is obtained using the Kozeny-Carman

equation.

$$K = \frac{10^{12} \phi r^2}{8\tau^2} \tag{10}$$

Where r is pore radius supporting fractures (m), τ is tortuosity (based on this configuration, it is a constant of 1.1547), K is fracture permeability (μm^2).

$$\tau = \left(\frac{L_w}{L}\right)^2 \tag{11}$$

Where L_w is actual length of capillary tube (m).

Substitute the porosity calculation equation 9 into 10 to obtain the single fracture permeability calculation equation, as follows:

$$K = \frac{\left[LHw - N\left(4\pi r_p^3/3\right)\right] r^2 \times 10^{12}}{8LHw\tau^2} \tag{12}$$

Calculation for pore radius of the propped fractures:

$$r = \frac{2}{n+1} r_1 + \frac{n-1}{n+1} r_2 \tag{13}$$

Where r_1 is fracture wall pore radius (m), r_2 is internal pore radius of fractures (m)

The pore channel near the fracture wall is enclosed by proppant hemisphere and the fracture plane, which is different from the pore channel composed of proppant inside the fracture. The calculation equation for the pore radius adjacent to the fracture wall is described as:

$$r_1 = \sqrt{\frac{2LHr_p - N_f \frac{2\pi r_p{}^3}{3}}{N_1 \pi L}} \tag{14}$$

Where N_1 is the number of pores on the fracture wall surface.

The pores inside the fracture are composed of proppants, and their calculation equation is as follows:

$$r_2 = \sqrt{\frac{LH\left[(n-1) 2r_p \frac{\sqrt{6}}{3}\right] - \left(N \frac{4\pi r_p{}^3}{3} - N_f \frac{2\pi r_p{}^3}{3}\right)}{N_2 \pi L}} \tag{15}$$

Where N_2 is number of internal pores in fracture.

$$N_1 = 2\left[\left(H-2r_p\right)/\left(\sqrt{3}r_p\right)\right]-C \tag{16}$$

$m=3i-2$, $C=0$; $m=3i-1/m=3i, C=1$.

$$N_2 = (n-1)\left[\left(H-2r_p\right)/\left(\sqrt{3}r_p\right)\right]-D \tag{17}$$

$m=3i-2/m=3i-1$, $D=2i-2$; $m=3i$, $D=2i-1$.

(6) Calculation of fracture conductivity

The equation for calculating the conductivity of a single fracture without considering embedding, proppant fragmentation, etc. is as follows:

$$Kw = \frac{\left[LHw - N\left(4\pi r_p^3/3\right)\right]r^2 \times 10^{12}}{8LH\tau^2} \tag{18}$$

The breakage of proppant is not considered in the analytical model of conductivity of single fracture. Based on experimental data from the "一 type" fracture network, the analytical model for single fracture conductivity is modified.

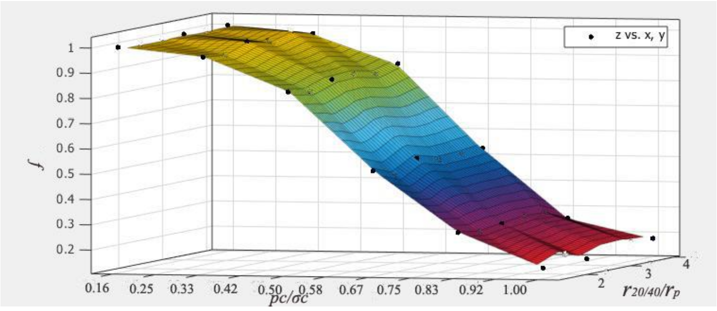

Fig 16. Results of the correction factor

Regression is performed to improve the accuracy of the derived analytical model and to apply for the facture network conductivity calculation. The z-values are the ratio of experimental measured conductivity to the calculated ones; the ratio of closure pressure to proppant compressive strength is treated as x; the ratio of proppant radius to 20-40 mesh proppant radius are plotted as the y-axis (Fig 16).

$$f = 0.1946e^{-\left(\frac{P_c/\sigma_c - 0.227}{0.56}\right)^2}\left(1.932\frac{r_{20/40}}{r_p} - 0.9304\right) \tag{19}$$

Where f is correction factor, P_c is closure pressure (MPa), σ_c is proppant compressive strength (MPa), $r_{20/40}$ is average radius of 20-40 mesh proppant (m), r_p

is average radius of proppant (m).

The modified equation for calculating the conductivity of single fracture is as follows:

$$(Kw)_f = \frac{\left[LHw - N\left(4\pi r_p^3/3\right)\right] r^2 \times 10^{12}}{8LH\tau^2} \cdot 0.1946 e^{-\left(\frac{P_c/\sigma_p - 0.227}{0.56}\right)^2} \left(1.932\frac{r_{20/40}}{r_p} - 0.9304\right) \tag{20}$$

Where $(Kw)_f$ is the conductivity of single fracture considering the closure pressure, proppant fragmentation (μm^2·cm).

The principle of hydraulic-electrical analogy is that the seepage law of fluid flow in porous media has a certain similarity to the law of charge flow in conductive materials. These two physical processes share similar forms of governing equation and flow states.

During the experiment, a fracture network was constructed using rock slabs and proppants, as shown in Fig 17.

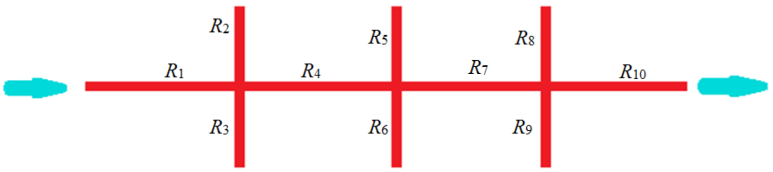

Fig 17. Schematics of "I" fracture network

Due to the equal inlet pressures of the main and secondary fractures, the incoming gas enters the fracture network from the main fracture R_1 and secondary fractures R_2, R_3, R_5, R_6, R_8, and R_9. The flow rate inside the fracture is inversely related to the seepage resistance. The gas flowing into the main fracture R_1 and secondary fractures R_2 and R_3 converges at the intersection point, and then flows into the main fracture R_4. Gas flows in other fractures, R_5~R_{10}, follows the similar patterns that are similar to the series-parallel current flow. In a parallel circuit, the potential is equal, and the current magnitude is inversely related to the resistance. In a series circuit, the current is equal, and the potential is additive (Fig. 18).

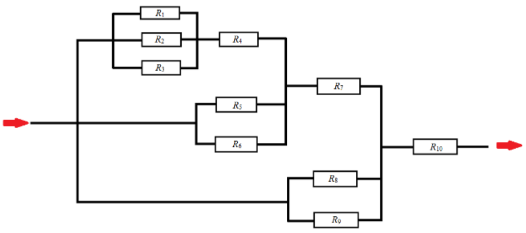

Fig 18. Equivalent circuit diagram of "I" fracture network

The total flow rate inside the chamber remains unchanged, and the inlet and outlet flow rates are equal. This is similar to the equal current in series-parallel circuits. The outlet pressure is affected by the structure of the fracture network and seepage resistance, similar to how the negative electrode potential in a circuit is affected by resistance and the structure of the circuit. Seepage resistance is influenced by factors like permeability, fracture length, and width, while electrical resistance is influenced by resistivity, resistance length, and cross-sectional area. The two are similar in this regard.

The analytical model of fracture network conductivity is established based on a single fracture conductivity model. Firstly, using the modified single fracture conductivity model (equations (3~20)), the conductivity of the main and secondary fractures is obtained. Then, using equations (21), the seepage resistance R_1~R_{3j+1} of the main and secondary fractures is calculated.

$$R_k = \frac{\mu L}{(Kw)_f H} \qquad (k=1、2、3\cdots\cdots 3j+1) \qquad (21)$$

Where μ is viscosity of air under standard conditions, 0.01789mPa.s, R_k is seepage resistance (mPa·s/(μm^2.cm)).

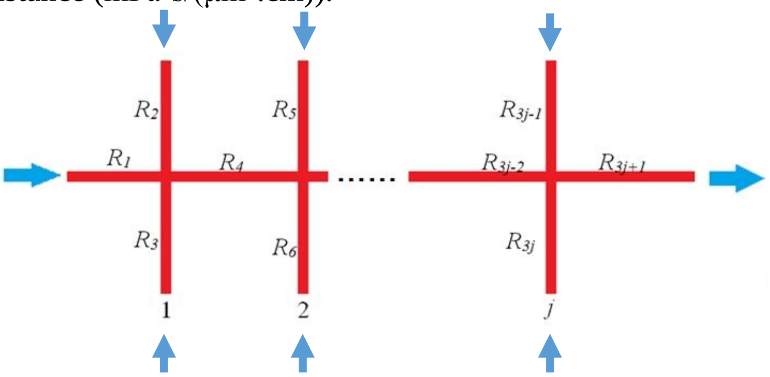

Fig 19. Schematics of shark's fin shape fracture network

According to the equivalent circuit diagram and Ohm's law, the seepage resistance is calculated. When the secondary fracture is closing or the sand concentration is zero, the seepage resistance of the crack is infinite.

As shown in Fig 19, there are 2*j* secondary fractures, and the general equation for calculating the seepage resistance is shown in equation 22:

$$
\begin{aligned}
&R_{t1} = \frac{1}{\dfrac{1}{R_1} + \dfrac{1}{R_2} + \dfrac{1}{R_3}} \qquad (j=1) \\
&R_{tj} = \frac{1}{\dfrac{1}{R_{t(j-1)} + R_{3j-2}} + \dfrac{1}{R_{3j-1}} + \dfrac{1}{R_{3j}}} \quad (j \geq 2) \\
&R = R_{tj} + R_{3j+1}
\end{aligned}
\tag{22}
$$

According to equations 22 obtain the seepage resistance of complex fracture networks, and then use equations 23 to obtain the permeability of complex fracture networks.

$$K_{nw} = \frac{\mu L}{RwH} \tag{23}$$

Where K_{nw} is the permeability of complex fracture network (μm^2).

$$F_{nwc} = K_{nw} w_f \tag{24}$$

Where F_{nwc} is the conductivity of complex fracture network (μm^2.cm).

4.2 Verification of the fracture network conductivity model

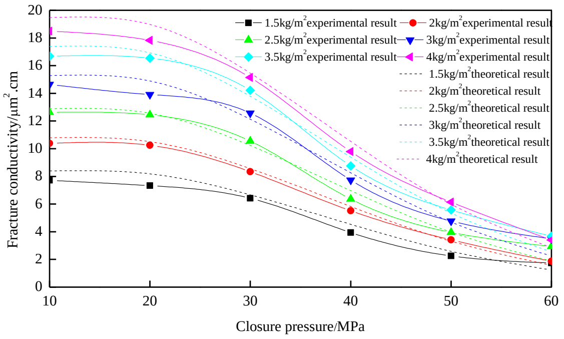

Fig 20. Comparison between theoretical and experimental results under different concentrations of "T" type

Figure 20 demonstrates strong congruence between the analytical model-predicted fracture network conductivity and experimental measurements. The error for different sand concentrations is about 13%. When the closure pressure is less than 50 MPa, the

root mean square error is 5.82%. Under high closing pressure, the error is relatively large, but the difference is small, and the error is within a reasonable range.

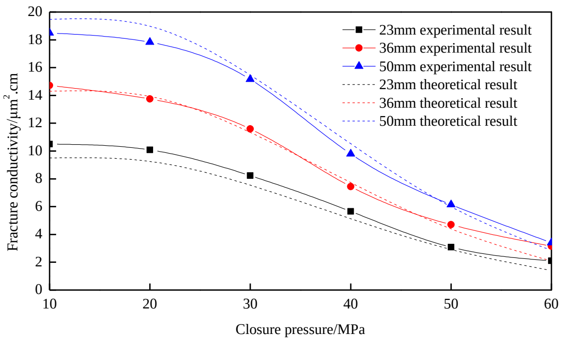

Fig 21. Comparison between theoretical and experimental results different fracture heights of "T" type

As shown in Fig 21, the theoretical calculation results still remain in good agreement with the experimental data curves under different fracture heights. The error between theoretical and experimental results across different fracture heights is 13.16%, indicating high accuracy. When the closure pressure is below 50 MPa, the theoretical and experimental results exhibit excellent consistency with minimal deviation, with an error of 5.53%.

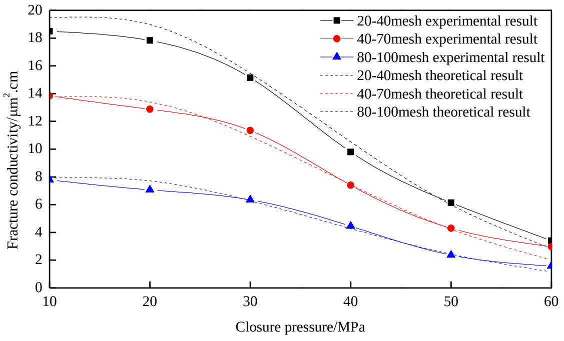

Fig 22. Comparison between theoretical and experimental results different proppant diameters of "T" type

From Fig 22, the root mean square error for different proppant diameters is 11.27%, which meets engineering accuracy requirements. Under low closure pressure conditions, the root mean square error is 3.96%, demonstrating high precision.

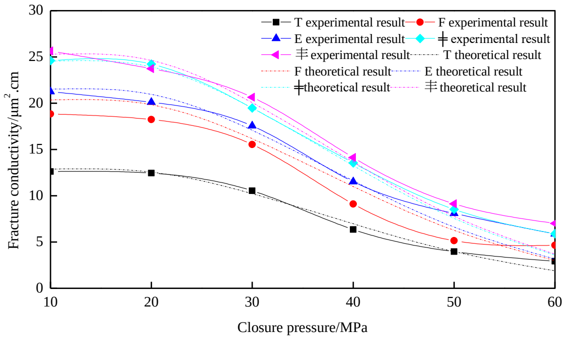

Fig 23. Comparison between theoretical and experimental results under different fracture structures

As shown in Figure 23, under identical conditions but different fracture network structures, the theoretical model results exhibit strong agreement with the experimental data. The root mean square error is 9.81%, with the primary source of error attributed to lower measurement accuracy under high closure pressure (60 MPa). At low closure pressures, the root mean square error decreases to 8.20%.

The new model established in this study demonstrates high accuracy, with a root mean square error below 14%, meeting the requirements for engineering applications. The theoretical results show excellent agreement with experimental data when the closure pressure is below 50 MPa. However, the accuracy decreases under high closure pressures, primarily due to limitations in the precision and reliability of experimental measurements under such conditions. Additionally, the new model assumes that all capillary flow pathways are horizontally oriented, whereas in reality, both horizontal and vertical capillaries exist within the proppant. At low pressure, gas predominantly flows along horizontal directions with minimal vertical movement. Under high closure pressures, proppant crushing increases gas flow through vertical capillaries, causing the model to underestimate experimental results and resulting in larger deviations.

4.3 Application of the fracture network conductivity model

The fracture conductivity of different numbers of secondary fractures was calculated based on the new model.

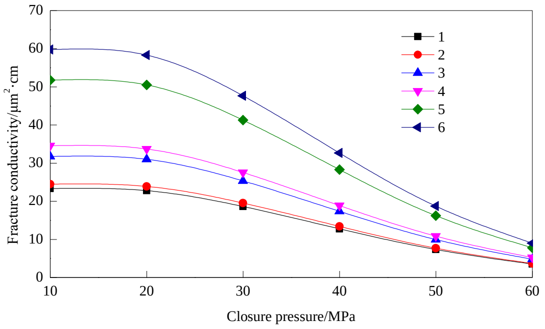

Fig 24. Conductivity of fracture network under different numbers of secondary fractures

From Fig 24, the more secondary fractures, the greater the conductivity of the fracture network. The increase of conductivity caused by the increase in the number of secondary fractures were 5%, 29.63%, 8.79%, 49.89%, and 15.45%, respectively. The more secondary fractures there are, the more seepage channels in the matrix increase, and the seepage resistance decreases.

The aspect ratio is defined as the ratio of the main fracture length to the secondary fracture length. This parameter governs the geometry of the fracture network and serves as a critical parameter in hydraulic fracturing design. In this study, aspect ratios of 2, 3, 4, 5, and 6 were investigated.

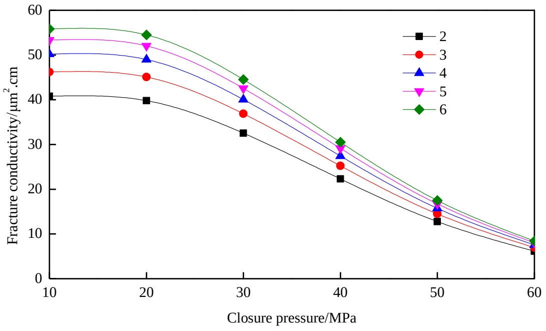

Fig.25 Conductivity of fracture network under different aspect ratio

As shown in Figure 25, the conductivity of fracture network increases with the aspect ratio. However, the rate of increase diminishes as the aspect ratio rises. At a closure pressure of 20 MPa, when the aspect ratio increases from 2 to 3, the conductivity rises from 39.82 $\mu m^2 \cdot cm$ to 45.10 $\mu m^2 \cdot cm$, representing a 13.26% improvement. In contrast, when the aspect ratio increases from 5 to 6, the conductivity only improves from 52.04 $\mu m^2 \cdot cm$ to 54.49 $\mu m^2 \cdot cm$, corresponding to a

smaller gain of 4.61%. These results indicate that while higher aspect ratios enhance conductivity, the incremental gain becomes progressively smaller. For practical fracture network design, it is recommended to select an aspect ratio between 4 and 5.

Conclusions

In this study, experiments are conducted to investigate the effects of critical influential factors on the conductivity of the fracture network. A new model of fracture network conductivity is established. The following conclusion can be drawn:

1. The experiment showed that as closure pressure increases, the conductivity of the fracture network decreases. Quartz sand proppants show a similar trend, but the conductivities are lower compared with ceramics.
2. The higher the sand concentration, the greater the conductivity. As the fracture height and proppant diameter increase, the conductivity increases. The closer the position of the secondary fracture is to the outlet, the greater the increase in the conductivity of the fracture network.
3. The orders of the conductivity is as follows: "-" type < "+"-1 type < "T" type < "+"-2 type < "F" type < "E" type < "+"-3 type < "╪" type < "Ɨ" type. Orthogonal experimental analysis shows that closure pressure and the position of secondary fractures significantly impact the conductivity of the fracture network. The proppant diameter, and sand concentration also have a greater impact, while the number of secondary fractures, type of proppant and fracture height have a weaker impact.
4. An analytical model of single fracture conductivity was established based on the Kozeny-Carman equation and experimental data. Based on the principle of hydroelectric similarity, a new model of the fracture network conductivity was ultimately established.
5. For fracture network fracturing optimization, it is recommended to use smaller proppant particle sizes (40-70 mesh) with lower proppant concentration and high-strength proppant. In fracture network design, an aspect ratio of 4~5 is

preferred, and multiple secondary fractures should be designed near the wellbore to enhance conductivity.

Conflicts of interest

The authors declare on conflict of interest.

Funding

This work was supported by the Natural Science Foundation of Sichuan Province under Grant 2023NSFSC0945. The funder had no role in study design, data collection and analysis, decision to publish, or preparation of the manuscript.

Data Availability Statement

All relevant data are within the paper and its Supporting Information files.

References

1. Lu S, Huang W, Chen F, Li J, Wang M, Xue H, et al. Classification and evaluation criteria of shale oil and gas resources: Discussion and application. Petroleum Exploration and Development. 2012; 39(2): 268-276.
2. Nobakht M, Clarkson CR. Analysis of production data in shale gas reservoirs: Rigorous corrections for fluid and flow properties. Journal of Natural Gas Science and Engineering. 2012; 8: 85-98.
3. Hui G, Chen Z, Schultz R, Chen S, Song Z, Zhang Z, et al. Intricate unconventional fracture networks provide fluid diffusion pathways to reactivate pre-existing faults in unconventional reservoirs. Energy. 2023; 282: 128803.
4. Chen M, Geng J, Zhao L. A hybrid numerical model for coupled hydro-mechanical analysis during CO2 injection into heterogeneous unconventional reservoirs. Gas Science and Engineering. 2024; 124: 205-244.
5. Zhao S, Zhao Q, Zhang X, Jiang X, Wu H, Yan J. Key policy needs for the success of China's shale gas revolution. Energy Reports. 2024; 11(40): 15-20.
6. Alhemdi A, Gu M. Method to account for natural fracture induced elastic anisotropy in geomechanical characterization of shale gas reservoirs. Journal of Natural Gas Science and Engineering. 2022;101:104478.

7. Zhao Y, Yang H, Wu J, Liu C, Chang C, Yu W, et al. Choke management simulation for shale gas reservoirs with complex natural fractures using EDFM. Journal of Natural Gas Science and Engineering. 2022;107:104801.
8. Zhao Y, Xiao-Tao W, Li C-L, Liu Y, Xie C-L. Systematic prediction of the gas content, fractures, and brittleness in fractured shale reservoirs with TTI medium. Petroleum Science. 2024.
9. Cipolla CL, Warpinski NR, Mayerhofer MJ, Lolon EP, Vincent MC, editors. The Relationship Between Fracture Complexity, Reservoir Properties, and Fracture Treatment Design. SPE Annual Technical Conference and Exhibition; 2008. SPE-115769-MS.
10. Ebrahimi AN, Wittel FK, Araújo NAM, Herrmann HJ. Multi-scale approach to invasion percolation of rock fracture networks. Journal of Hydrology. 2014; 519: 353-63.
11. Liu Y, Xiu N, Ding Y, Wang X, Lu Y, Dou J, et al. Analysis of multi-factor coupling effect on hydraulic fracture network in shale reservoirs. Natural Gas Industry. 2015; 2(2): 162-166.
12. Ren L, Wang Z, Zhao J, Lin R, Wu J, Song Y, et al. Shale gas effective fracture network volume prediction and analysis based on flow back data: A case study of southern Sichuan Basin shale. Geoenergy Science and Engineering. 2023; 228: 211963.
13. Matthäi SK, Bui CM, Hansika H, Perera MSA. Influence of inertial and centrifugal forces on rate and flow patterns in natural fracture networks. Advances in Water Resources. 2024: 104741.
14. Liu X. Shale-gas well test analysis and evaluation after hydraulic fracturing by stimulated reservoir volume (SRV). Natural Gas Industry . 2016; 3(6): 577-584.
15. Zhang Q, Su Y, Wang W, Lu M, Ren L. Performance analysis of fractured wells with elliptical SRV in shale reservoirs. Journal of Natural Gas Science and Engineering. 2017; 45: 380-390.
16. Jiang R, Zhang F, Cui Y, Qiao X, Zhang C. Production performance analysis of fractured vertical wells with SRV in triple media gas reservoirs using elliptical flow. Journal of Natural Gas Science and Engineering. 2019; 68: 102925.
17. Liu X, Jin Y, Lin B. Classification and evaluation for stimulated reservoir volume (SRV) estimation models using microseismic events based on three typical grid structures. Journal of Petroleum Science and Engineering. 2022; 211: 110-169.
18. Wen Q, Zhang S, Wang L, Liu Y. Influenceof proppant embedment on fracture long-term flow conductivity. Natural Gas Industry. 2005; 25(05): 65-8+9-10.
19. Lu Q, He Y, Xu W, Tian R, Zhang F. Volume Fracturing Fracture Network Configuration's

Influence on Fracture Diverting Capacity. Journal of Changzhou University (Natural Science Edition). 2014; 26(02): 70-74..

20. Wen Q, Wang S, Duan X, Li Y, Wang F, Jin X. Experimental investigation of proppant settling in complex hydraulic-natural fracture system in shale reservoirs. Journal of Natural Gas Science and Engineering. 2016;33:70-80.

21. Liang X, Zhou F, Liang T, Huang Y, Wei D, Ma S. The effect of combined proppants upon the fracture conductivity in tight gas reservoirs. Energy Reports. 2020; 6: 879-884.

22. Wang Z, Lin B, Chen G, Dai Y, Chen A. Experimental study on evaluation of conglomerate reservoir support-type fracture conductivity in Xinjiang oilfield. Energy. 2023; 278: 127877.

23. Shi X, Ge X, Zhang Y, Huang W, Zhan Y, Gu Y, et al. Experimental study of propped fracture conductivity in tight dolomite reservoir. Petroleum Geology and Recovery Efficiency. 2023; 30(04): 167-172.

24. Katende A, Allen C, Rutqvist J, Nakagawa S, Radonjic M. Experimental and numerical investigation of proppant embedment and conductivity reduction within a fracture in the Caney Shale, Southern Oklahoma, USA. Fuel. 2023; 341: 127571.

25. Liu K, He H, Yin D. Theoretical and experimental study on the long-term conductivity of heterogeneous artificial fractures in tight reservoirs. Energy Reports. 2023; 9: 3881-3895.

26. Li K, Gao Y, Lyu Y, Wang M. New Mathematical Models for Calculating Proppant Embedment and Fracture Conductivity. SPE Journal. 2015; 20(03): 496-507.

27. Liu Z, Zhao H, Wang D, Yuan P, He Y. Establishment and application of propped hydraulic fracture conductivity theoretical model based on fracturing efficiency index. Gas Science and Engineering. 2024; 121: 199-205.

28. Liu Y, Wu L, Guo J, He S, Wu Y. Model for fracture conductivity considering particle size redistribution caused by proppant crushing. Geoenergy Science and Engineering. 2024; 240: 213081.

29. Nwabia FN, Leung JY. A Practical probabilistic history matching framework for characterizing fracture network parameters of shale gas reservoirs. Gas Science and Engineering. 2024; 126: 305-26.

30. Sun Z, Wang H, Zhang B, Li Y, Peng Z, Zhang S. Intelligent construction method and application of large-scale three-dimensional complex discrete fracture network model based on particle

swarm optimization algorithm. Computers and Geotechnics. 2024; 171: 306-16.

31. Tan Y, Pan Z, Liu J, Feng X-T, Connell LD. Laboratory study of proppant on shale fracture permeability and compressibility. Fuel. 2018;222:83-97

32. Wu D, Li S, Zhang N, Guo Y, Liu L, Wang Z. A novel permeability model for hydrate-bearing sediments integrating pore morphology evolution based on modified Kozeny-Carman equation. Energy. 2023; 277: 127723.

33. Xiao B, Zhu H, Chen F, Long G, Li Y. A fractal analytical model for Kozeny-Carman constant and permeability of roughened porous media composed of particles and converging-diverging capillaries. Powder Technology. 2023; 420: 118256.

Response to Reviewers and Editor

Journal: **PLOS ONE**

Manuscript ID: **PONE-D-25-07188**

Paper Title: **Experimental and theoretical investigation on the conductivity of complex fracture network in unconventional gas reservoirs**

Authors: **Jinjian Gao, Lanxiao Hu**

Dear Editor and Reviewers,

First and foremost, I would like to express my sincere gratitude for the thorough review and insightful comments provided on our manuscript titled " Experimental and theoretical investigation on the conductivity of complex fracture network in unconventional gas reservoirs ". Those comments are all valuable and very helpful for revising and improving our paper, as well as the important guiding significance to our researches. We have carefully considered each point raised and have made the following revisions to our manuscript.

Academic Editor

Comment: *Thank you for submitting your manuscript to PLOS ONE. After careful consideration, we feel that it has merit but does not fully meet PLOS ONE's publication criteria as it currently stands. Therefore, we invite you to submit a revised version of the manuscript that addresses the points raised during the review process.*

Response: We thank the Academic Editor for allowing us to revise our manuscript. We would also thank the reviewers for their valuable comments that help us substantially improve the quality of our manuscript. The reviewers' comments are responded to points-by-points, and the manuscript has been thoroughly revised based on the comments and suggestions made by the two reviewers.

Reviewer #1

Comment #1: *The control range of some experimental variables can be further expanded when studying the influence of different factors on the conductivity of fracture networks. For example, more proppant types or wider closure pressure intervals can be added to obtain more comprehensive data.*

Response: Thank you very much for your constructive suggestions. We sincerely appreciate your insightful feedback on improving the comprehensiveness of our study. Regarding your suggestion to expand the control range of experimental variables (e.g., including more proppant types or wider closure pressure intervals), we regret to inform you that due to current experimental limitations, it is challenging to further broaden the experimental parameters in this study. However, based on extensive literature reviews and preliminary research, unconventional reservoirs commonly employ low sand ratios and small-sized proppants during fracturing operations. Therefore, the proppant concentration in this study was set to 1.5~4 kg/m², which aligns with field practices. Additionally, when closure pressure exceeds 50 MPa, most types of proppants are significantly crushed, leading to minimal changes in fracture conductivity as closure pressure further increases. This phenomenon has been widely reported in prior studies (Tan Y, Pan Z, Liu J, Feng X-T, Connell LD. Laboratory study of proppant on shale fracture permeability and compressibility. Fuel. 2018; 222: 83-97.). For this reason, the closure pressure range in our experiments was selected as 10~60 MPa to balance practical relevance and experimental feasibility.

Once again, we deeply appreciate your valuable suggestions, which have highlighted important directions for our future research. We will prioritize expanding the experimental variables (e.g., proppant types and pressure ranges) in subsequent studies to obtain more comprehensive insights into fracture network conductivity.

Comment #2:. *In the process of model validation, the comparison analysis with actual gas reservoir production data can be added in addition to the comparison with experimental data.*

Response: Thank you for your valuable feedback and constructive suggestion to enhance the rigor of our model validation. We appreciate your insight into the importance of comparing experimental results with actual gas reservoir production data.

However, we regret that we are currently unable to supplement the manuscript with such a comparison due to the unavailability of field production data. Gas reservoir production data, such as hydrocarbon yields, are typically confidential or proprietary, making them challenging to acquire for academic research. To perform a meaningful comparison, our conductivity data would need to be integrated with a conductivity-to-production model to predict dynamic production behavior, which would then be validated against field data. This process constitutes a significant standalone study that requires extensive collaboration with industry partners, and we plan to address it as a dedicated follow-up research project.

To strengthen the validation of our current work, we have expanded the model verification by incorporating different fracture structures, fracture heights and proppant diameters. These additions ensure the robustness of our model in capturing key physical mechanisms under variable fracture conditions.

Changes: Please see lines 523-542 on pages 25 and 26, marked with yellow highlighting.

Comment #3:. *This paper does not fully discuss the influence of complex geological conditions (such as different rock types, stress distribution, etc.) on the conductivity of fracture networks.*

Response: The authors thank the reviewer for raising these points. In this experiment, rock slabs with high brittleness and high Young's modulus were used. There was less proppant embedment, and the fracture conductivity was greater than that of rock slabs with low Young's modulus. The closure pressure is generally the minimum principal stress. The smaller the minimum stress is, the greater the fracture conductivity will be. We have added some analyses of model applications.

The aspect ratio is defined as the ratio of the main fracture length to the secondary fracture length. This parameter governs the geometry of the fracture network and serves as a critical parameter in hydraulic fracturing design. In this study, aspect ratios of 2, 3, 4, 5, and 6 were investigated.

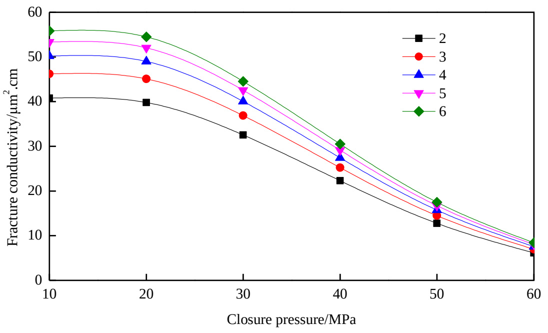

Fig.25 Curve of influence of aspect ratio on fracture conductivity

As shown in Figure 25, the conductivity of fracture network increases with the aspect ratio. However, the rate of increase diminishes as the aspect ratio rises. At a closure pressure of 20 MPa, when the aspect ratio increases from 2 to 3, the conductivity rises from 39.82 $\mu m^2 \cdot cm$ to 45.10 $\mu m^2 \cdot cm$, representing a 13.26% improvement. In contrast, when the aspect ratio increases from 5 to 6, the conductivity only improves from 52.04 $\mu m^2 \cdot cm$ to 54.49 $\mu m^2 \cdot cm$, corresponding to a smaller gain of 4.61%. These results indicate that while higher aspect ratios enhance conductivity, the incremental gain becomes progressively smaller. For practical fracture network design, it is recommended to select an aspect ratio between 4 and 5.

Changes: Please see lines 206-210 on pages 9, lines 565-579 on pages 27 marked with yellow highlighting.

Comment #4:. *The experimental scheme does not fully explain the selection basis of sand concentration, fracture height, proppant particle size, and other factors.*

Response: The authors thank the reviewer for suggestion. We have already explained the selection basis. Unconventional reservoirs commonly employ low sand ratios and small-sized proppants during fracturing operations [31]. The rock slabs provided by the laboratory can only be processed into sizes of 23mm, 36mm and 50mm. The length-to-height ratio of fractures was designed based on field observations and theoretical frameworks widely adopted in unconventional reservoir engineering. Specifically, we focused on a range of 0.14 to 0.35 for this ratio, which aligns with typical fracture geometries observed in shale and tight sandstone reservoirs under similar stress conditions (Cipolla CL, Warpinski NR, Mayerhofer MJ, Lolon EP, Vincent MC, editors. The Relationship Between Fracture Complexity, Reservoir Properties, and Fracture Treatment Design. SPE Annual Technical Conference and Exhibition; 2008. SPE-115769-MS).

Changes: Please see lines 168-177 on pages 7 marked with yellow highlighting.

Comment #5:. *In the part of model validation, the adaptability of the model under different fracture network structures and different working conditions is not fully analyzed..*

Response: The authors thank the reviewer for suggestion We supplemented the validation under the conditions of different fracture network structures, proppant diameters, and fracture heights.

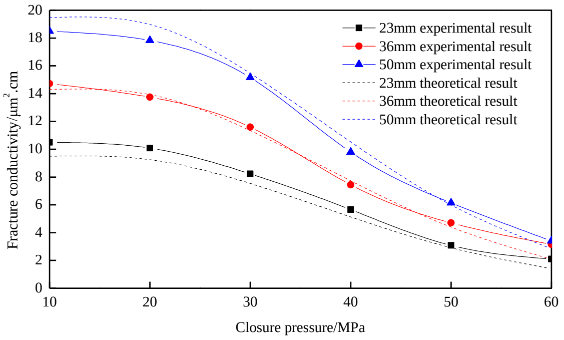

Fig 21. Comparison between theoretical and experimental results different fracture heights of "T" type

As shown in Fig 21, the theoretical calculation results still remain in good agreement with the experimental data curves under different fracture heights. The error between theoretical and experimental results across different fracture heights is 13.16%, indicating high accuracy. When the closure pressure is below 50 MPa, the theoretical and experimental results exhibit excellent consistency with minimal deviation, with an error of 5.53%.

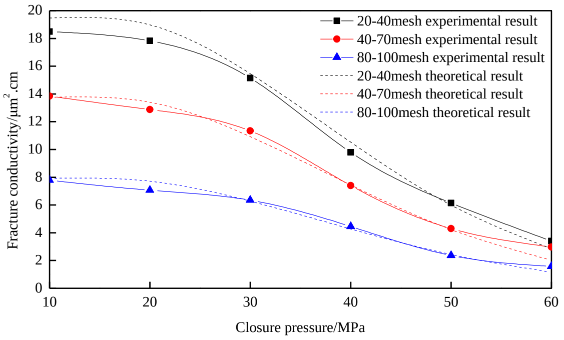

Fig 22. Comparison between theoretical and experimental results different proppant diameters of "T" type

From Fig 22, the root mean square error for different proppant diameters is 11.27%, which meets engineering accuracy requirements. Under low closure pressure conditions, the root mean square error is 3.96%, demonstrating high precision.

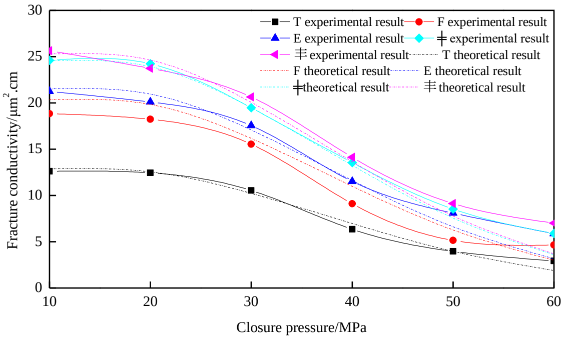

Fig 23. Comparison between theoretical and experimental results under different fracture structures

As shown in Figure 23, under identical conditions but different fracture network structures, the theoretical model results exhibit strong agreement with the experimental data. The root mean square error is 9.81%, with the primary source of error attributed to lower measurement accuracy under high closure pressure (60 MPa). At low closure pressures, the root mean square error decreases to 8.20%.

Changes: Please see lines 523-554 on pages 25 and 26, marked with yellow highlighting.

Reviewer #2

Comment #1:. *The paper designs a novel flow diversion chamber and establishes a fracture network conductivity model, showing some innovation. However, the abstract should more explicitly emphasize its breakthrough points compared to existing studies.*

Response: The authors thank the reviewer for comment. Compared with existing experimental studies focusing on single fracture conductivity, this study presents

experimental research on the conductivity of complex fracture network, thereby addressing a research gap in this field. Through synergistic integration of the Kozeny-Carman hydrodynamic theory with hydraulic-electrical analogy principles, an innovative model of fracture network conductivity is formulated. The advantage of new model of fracture network conductivity is that it combines a theoretical method and an experimental method.

Changes: Please see lines 34-41 on pages 2 marked with yellow highlighting.

Comment #2:. *The selection of flow chamber material (stainless steel) lacks justification for whether its compressive performance meets the requirement of 100 MPa closure pressure. Please supplement material mechanical parameters.*

Response: The authors thank the reviewer for comment. We have supplemented the compressive strength of stainless steel.

Changes: Please see lines 125-126 on pages 5 marked with yellow highlighting.

Comment #3:. *The orthogonal experiment does not clarify the rationale for selecting specific factor levels (e.g., fracture heights of 23/36/50 mm). Provide basis for parameter selection.*

Response: The authors thank the reviewer for comment. We have provided basis for parameter selection.

Because unconventional reservoirs commonly employ low sand ratios and small-sized proppants during fracturing operations [31]. The proppant is 20-40 mesh ceramic proppant. The experimental scheme for changing the fracture height is as follows: the fracture heights are 23, 36 and 50 mm, with a sand concentration of 4 kg/m². The rock slabs provided by the laboratory can only be processed into sizes of 23, 36, and 50mm. The length-to-height ratio of fractures was designed based on field observations and theoretical frameworks widely adopted in unconventional reservoir engineering. Specifically, we focused on a range of 0.14 to 0.35 for this ratio, which aligns with typical fracture geometries observed in shale and tight sandstone reservoirs under similar stress conditions.

This study considered seven influencing factors with the assumption of no interactions between them. Among these factors, proppant type was assigned two distinct levels, while the remaining six factors each had three levels. Through orthogonal array reference tables, the optimal experimental layout was identified as $L18(2\times3^7)$. The designed orthogonal experiments evaluated outcomes using conductivity as the performance metric, with detailed results provided in Table 3.

Changes: Please see lines 168-177 on pages 7; lines 314-319 on pages 15 marked with yellow highlighting.

Comment #4:. *The Kozeny-Carman equation assumes the proppant pack as a uniform capillary tube model, but actual fracture networks may have non-uniform distributions. Discuss potential impacts of this assumption on model accuracy.*

Response: The authors thank the reviewer for comment. The new model assumes that all capillary are horizontal and uniform. In reality, both horizontal and vertical capillaries exist within the proppant. At low pressure, gas predominantly mainly flows along horizontal directions. Under high closure pressures, proppant crushing increases gas flow through vertical capillaries, leading to the decrease of the model's accuracy.

Changes: Please see lines 363-368 on pages 17 marked with yellow highlighting.

Comment #5:. *Figure 20 only validates the "T-shaped" fracture network. Include comparisons with other types (e.g., "I-shaped," "E-shaped") to demonstrate universality.*

Response: The authors thank the reviewer for comment. We supplemented the verification under the conditions of different fracture network structures, proppant diameters, and fracture heights.

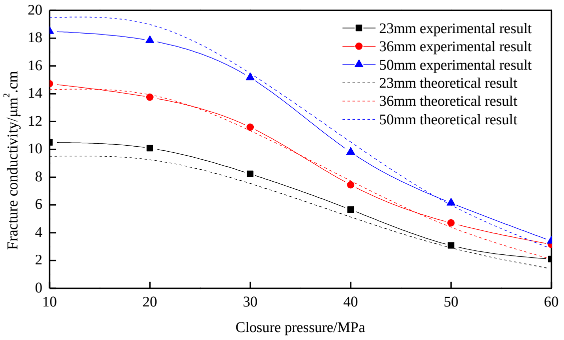

Fig 21. Comparison between theoretical and experimental results different fracture heights of "T" type

As shown in Fig 21, the theoretical calculation results still remain in good agreement with the experimental data curves under different fracture heights. The error between theoretical and experimental results across different fracture heights is 13.16%, indicating high accuracy. When the closure pressure is below 50 MPa, the theoretical and experimental results exhibit excellent consistency with minimal deviation, with an error of 5.53%.

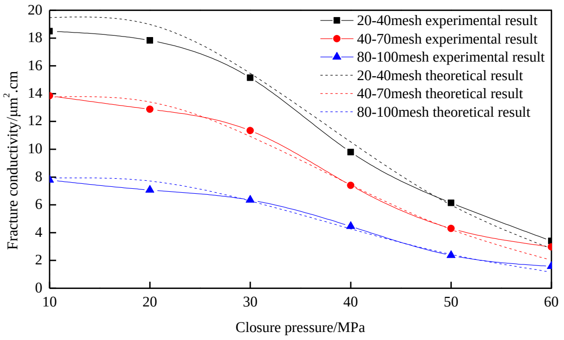

Fig 22. Comparison between theoretical and experimental results different proppant diameters of "T" type

From Fig 22, the root mean square error for different proppant diameters is 11.27%,

which meets engineering accuracy requirements. Under low closure pressure conditions, the root mean square error is 3.96%, demonstrating high precision.

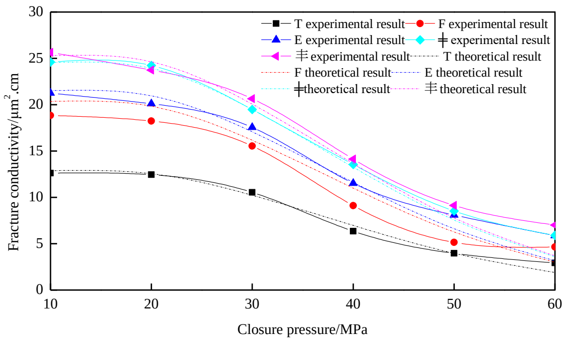

Fig 23. Comparison between theoretical and experimental results under different fracture structures

As shown in Figure 23, under identical conditions but different fracture network structures, the theoretical model results exhibit strong agreement with the experimental data. The root mean square error is 9.81%, with the primary source of error attributed to lower measurement accuracy under high closure pressure (60 MPa). At low closure pressures, the root mean square error decreases to 8.20%.

Changes: Please see lines 523-542 on pages 25 and 26 marked with yellow highlighting.

Comment #6:. *The model exhibits significant errors (13%) under high closure pressure, yet the error sources are not thoroughly analyzed. Add discussion on error origins..*

Response: The authors thank the reviewer for comment. The accuracy decreases under high closure pressures, primarily due to limitations in the precision and reliability of experimental measurements under such conditions. Additionally, the new model assumes that all capillary flow pathways are horizontally oriented, whereas in reality, both horizontal and vertical capillaries exist within the proppant. At low pressure, gas predominantly flows along horizontal directions with minimal vertical movement. Under high closure pressures, proppant crushing increases gas flow through vertical capillaries, causing the model to underestimate experimental results and resulting in larger deviations.

Changes: Please see lines 543-554 on pages 26 marked with yellow highlighting.

Comment #7:. *The critical F-value criteria in Table 4 are undefined. Specify the statistical testing basis.*

Response: The authors thank the reviewer for comment. The F critical value is selected according to the F value critical value table with a confidence interval of 0.01.

Changes: Please see lines 321-322 on pages 15 marked with yellow highlighting and Table 4.

Comment #8:. *The conclusion lacks clarity on how the findings guide field fracturing optimization (e.g., optimal proppant size selection, fracture network design). Include engineering application recommendations.*

Response: The authors thank the reviewer for comment. We added one conclusion: For fracture network fracturing optimization, it is recommended to use smaller proppant particle sizes (40-70 mesh) with lower proppant concentration and high-strength proppant. In fracture network design, an aspect ratio of 4~5 is preferred, and multiple secondary fractures should be designed near the wellbore to enhance conductivity.

Changes: Please see lines 602-606 on pages 28 marked with yellow highlighting.

Comment #9:. *Author abbreviations in some references (e.g., Refs. 23, 27) are inconsistent. Standardize formatting.*

Response: The authors thank the reviewer for carefully reading. We have revised the format and corrected the errors in the references according to the requirements of journal references.

Changes: Please see lines 672-674, lines 682-684 on pages 31 marked with yellow highlighting.

Comment #10:. *Some sentences contain grammatical errors (e.g., "Due the complex structure" should be "Due to..."). Perform full-text proofreading.*

Response: The authors thank the reviewer for reminder. We have corrected the errors. We have double-checked the manuscript and corrected the grammar errors in the revised manuscript. We also have revised our manuscript thoroughly, and the language is refined to make the manuscript more concise and logical.

Changes: Please see the revised manuscript and lines 225-226 on pages 10 marked with yellow highlighting.

Sincerely

Jinjin Gao
