# Supplementary material for: Experimental and theoretical investigation on the conductivity of complex fracture network in unconventional gas reservoirs
Source: PLoS One. 2025 Jun 13;20(6):e0325825. doi: 10.1371/journal.pone.0325825 (PMC12165408; doi:10.1371/journal.pone.0325825)
Supplement: Attachment — Submitted filename: Response to Reviewers and Editor .docx [file pone.0325825.s004.docx]

**Response to Reviewers and Editor**

Journal:	**PLOS ONE**

Manuscript ID:	**PONE-D-25-07188**

Paper Title:	**Experimental and theoretical investigation on the conductivity of complex fracture network in unconventional gas reservoirs**

Authors:	**Jinjian Gao, Lanxiao Hu**

Dear Editor and Reviewers,

First and foremost, I would like to express my sincere gratitude for the thorough review and insightful comments provided on our manuscript titled " Experimental and theoretical investigation on the conductivity of complex fracture network in unconventional gas reservoirs ". Those comments are all valuable and very helpful for revising and improving our paper, as well as the important guiding significance to our researches. We have carefully considered each point raised and have made the following revisions to our manuscript.

**Academic Editor**

**Comment:** *Thank you for submitting your manuscript to PLOS ONE. After careful consideration, we feel that it has merit but does not fully meet PLOS ONE’s publication criteria as it currently stands. Therefore, we invite you to submit a revised version of the manuscript that addresses the points raised during the review process.*

**Response:** We thank the Academic Editor for allowing us to revise our manuscript. We would also thank the reviewers for their valuable comments that help us substantially improve the quality of our manuscript. The reviewers' comments are responded to points-by-points, and the manuscript has been thoroughly revised based on the comments and suggestions made by the two reviewers.

**Reviewer #1**

**Comment #1:** *The control range of some experimental variables can be further expanded when studying the influence of different factors on the conductivity of fracture networks. For example, more proppant types or wider closure pressure intervals can be added to obtain more comprehensive data.*

**Response:** Thank you very much for your constructive suggestions. We sincerely appreciate your insightful feedback on improving the comprehensiveness of our study. Regarding your suggestion to expand the control range of experimental variables (e.g., including more proppant types or wider closure pressure intervals), we regret to inform you that due to current experimental limitations, it is challenging to further broaden the experimental parameters in this study. However, based on extensive literature reviews and preliminary research, unconventional reservoirs commonly employ low sand ratios and small-sized proppants during fracturing operations. Therefore, the proppant concentration in this study was set to 1.5~4 kg/m², which aligns with field practices. Additionally, when closure pressure exceeds 50 MPa, most types of proppants are significantly crushed, leading to minimal changes in fracture conductivity as closure pressure further increases. This phenomenon has been widely reported in prior studies (Tan Y, Pan Z, Liu J, Feng X-T, Connell LD. Laboratory study of proppant on shale fracture permeability and compressibility. Fuel. 2018; 222: 83-97. ). For this reason, the closure pressure range in our experiments was selected as 10~60 MPa to balance practical relevance and experimental feasibility.

Once again, we deeply appreciate your valuable suggestions, which have highlighted important directions for our future research. We will prioritize expanding the experimental variables (e.g., proppant types and pressure ranges) in subsequent studies to obtain more comprehensive insights into fracture network conductivity.

**Comment #2:***. In the process of model validation, the comparison analysis with actual gas reservoir production data can be added in addition to the comparison with experimental data.*

**Response:** Thank you for your valuable feedback and constructive suggestion to enhance the rigor of our model validation. We appreciate your insight into the importance of comparing experimental results with actual gas reservoir production data.

However, we regret that we are currently unable to supplement the manuscript with such a comparison due to the unavailability of field production data. Gas reservoir production data, such as hydrocarbon yields, are typically confidential or proprietary, making them challenging to acquire for academic research. To perform a meaningful comparison, our conductivity data would need to be integrated with a conductivity-to-production model to predict dynamic production behavior, which would then be validated against field data. This process constitutes a significant standalone study that requires extensive collaboration with industry partners, and we plan to address it as a dedicated follow-up research project.

To strengthen the validation of our current work, we have expanded the model verification by incorporating different fracture structures, fracture heights and proppant diameters. These additions ensure the robustness of our model in capturing key physical mechanisms under variable fracture conditions.

**Changes:** Please see lines 523-542 on pages 25 and 26, marked with yellow highlighting.

**Comment #3:***. This paper does not fully discuss the influence of complex geological conditions (such as different rock types, stress distribution, etc.) on the conductivity of fracture networks.*

**Response:** The authors thank the reviewer for raising these points. In this experiment, rock slabs with high brittleness and high Young's modulus were used. There was less proppant embedment, and the fracture conductivity was greater than that of rock slabs with low Young's modulus. The closure pressure is generally the minimum principal stress. The smaller the minimum stress is, the greater the fracture conductivity will be. We have added some analyses of model applications.

The aspect ratio is defined as the ratio of the main fracture length to the secondary fracture length. This parameter governs the geometry of the fracture network and serves as a critical parameter in hydraulic fracturing design. In this study, aspect ratios of 2, 3, 4, 5, and 6 were investigated.

**Fig.25 Curve of influence of aspect ratio on fracture conductivity**

As shown in Figure 25, the conductivity of fracture network increases with the aspect ratio. However, the rate of increase diminishes as the aspect ratio rises. At a closure pressure of 20 MPa, when the aspect ratio increases from 2 to 3, the conductivity rises from 39.82 μm²·cm to 45.10 μm²·cm, representing a 13.26% improvement. In contrast, when the aspect ratio increases from 5 to 6, the conductivity only improves from 52.04 μm²·cm to 54.49 μm²·cm, corresponding to a smaller gain of 4.61%. These results indicate that while higher aspect ratios enhance conductivity, the incremental gain becomes progressively smaller. For practical fracture network design, it is recommended to select an aspect ratio between 4 and 5.

**Changes:** Please see lines 206-210 on pages 9, lines 565-579 on pages 27 marked with yellow highlighting.

**Comment #4:***. The experimental scheme does not fully explain the selection basis of sand concentration, fracture height, proppant particle size, and other factors.*

**Response:** The authors thank the reviewer for suggestion. We have already explained the selection basis. Unconventional reservoirs commonly employ low sand ratios and small-sized proppants during fracturing operations ^[31]^. The rock slabs provided by the laboratory can only be processed into sizes of 23mm, 36mm and 50mm. The length-to-height ratio of fractures was designed based on field observations and theoretical frameworks widely adopted in unconventional reservoir engineering. Specifically, we focused on a range of 0.14 to 0.35 for this ratio, which aligns with typical fracture geometries observed in shale and tight sandstone reservoirs under similar stress conditions (Cipolla CL, Warpinski NR, Mayerhofer MJ, Lolon EP, Vincent MC, editors. The Relationship Between Fracture Complexity, Reservoir Properties, and Fracture Treatment Design. SPE Annual Technical Conference and Exhibition; 2008. SPE-115769-MS).

**Changes:** Please see lines 168-177 on pages 7 marked with yellow highlighting.

**Comment #5:***. In the part of model validation, the adaptability of the model under different fracture network structures and different working conditions is not fully analyzed..*

**Response:** The authors thank the reviewer for suggestion We supplemented the validation under the conditions of different fracture network structures, proppant diameters, and fracture heights.

**Fig 21. Comparison between theoretical and experimental results different fracture heights of "T" type**

As shown in Fig 21, the theoretical calculation results still remain in good agreement with the experimental data curves under different fracture heights. The error between theoretical and experimental results across different fracture heights is 13.16%, indicating high accuracy. When the closure pressure is below 50 MPa, the theoretical and experimental results exhibit excellent consistency with minimal deviation, with an error of 5.53%.

**Fig 22. Comparison between theoretical and experimental results different** **proppant diameters of "T" type**

From Fig 22, the root mean square error for different proppant diameters is 11.27%, which meets engineering accuracy requirements. Under low closure pressure conditions, the root mean square error is 3.96%, demonstrating high precision.

**Fig 23. Comparison between theoretical and experimental results under different** **fracture structures**

As shown in Figure 23, under identical conditions but different fracture network structures, the theoretical model results exhibit strong agreement with the experimental data. The root mean square error is 9.81%, with the primary source of error attributed to lower measurement accuracy under high closure pressure (60 MPa). At low closure pressures, the root mean square error decreases to 8.20%.

**Changes:** Please see lines 523-554 on pages 25 and 26, marked with yellow highlighting.

**Reviewer #2**

**Comment #1:***. The paper designs a novel flow diversion chamber and establishes a fracture network conductivity model, showing some innovation. However, the abstract should more explicitly emphasize its breakthrough points compared to existing studies.*

**Response:** The authors thank the reviewer for comment. Compared with existing experimental studies focusing on single fracture conductivity, this study presents experimental research on the conductivity of complex fracture network, thereby addressing a research gap in this field. Through synergistic integration of the Kozeny-Carman hydrodynamic theory with hydraulic-electrical analogy principles, an innovative model of fracture network conductivity is formulated. The advantage of new model of fracture network conductivity is that it combines a theoretical method and an experimental method.

**Changes:** Please see lines 34-41 on pages 2 marked with yellow highlighting.

**Comment #2:***. The selection of flow chamber material (stainless steel) lacks justification for whether its compressive performance meets the requirement of 100 MPa closure pressure. Please supplement material mechanical parameters.*

**Response:** The authors thank the reviewer for comment. We have supplemented the compressive strength of stainless steel.

**Changes:** Please see lines 125-126 on pages 5 marked with yellow highlighting.

**Comment #3:***. The orthogonal experiment does not clarify the rationale for selecting specific factor levels (e.g., fracture heights of 23/36/50 mm). Provide basis for parameter selection.*

**Response:** The authors thank the reviewer for comment. We have provided basis for parameter selection.

Because unconventional reservoirs commonly employ low sand ratios and small-sized proppants during fracturing operations ^[31]^. The proppant is 20-40 mesh ceramic proppant. The experimental scheme for changing the fracture height is as follows: the fracture heights are 23, 36 and 50 mm, with a sand concentration of 4 kg/m². The rock slabs provided by the laboratory can only be processed into sizes of 23, 36, and 50mm. The length-to-height ratio of fractures was designed based on field observations and theoretical frameworks widely adopted in unconventional reservoir engineering. Specifically, we focused on a range of 0.14 to 0.35 for this ratio, which aligns with typical fracture geometries observed in shale and tight sandstone reservoirs under similar stress conditions.

This study considered seven influencing factors with the assumption of no interactions between them. Among these factors, proppant type was assigned two distinct levels, while the remaining six factors each had three levels. Through orthogonal array reference tables, the optimal experimental layout was identified as L18(2×3⁷). The designed orthogonal experiments evaluated outcomes using conductivity as the performance metric, with detailed results provided in Table 3.

**Changes:** Please see lines 168-177 on pages 7; lines 314-319 on pages 15 marked with yellow highlighting.

**Comment #4:***. The Kozeny-Carman equation assumes the proppant pack as a uniform capillary tube model, but actual fracture networks may have non-uniform distributions. Discuss potential impacts of this assumption on model accuracy.*

**Response:** The authors thank the reviewer for comment. The new model assumes that all capillary are horizontal and uniform. In reality, both horizontal and vertical capillaries exist within the proppant. At low pressure, gas predominantly mainly flows along horizontal directions. Under high closure pressures, proppant crushing increases gas flow through vertical capillaries, leading to the decrease of the model's accuracy.

**Changes:** Please see lines 363-368 on pages 17 marked with yellow highlighting.

**Comment #5:***. Figure 20 only validates the "T-shaped" fracture network. Include comparisons with other types (e.g., "I-shaped," "E-shaped") to demonstrate universality.*

**Response:** The authors thank the reviewer for comment. We supplemented the verification under the conditions of different fracture network structures, proppant diameters, and fracture heights.

**Fig 21. Comparison between theoretical and experimental results different fracture heights of "T" type**

As shown in Fig 21, the theoretical calculation results still remain in good agreement with the experimental data curves under different fracture heights. The error between theoretical and experimental results across different fracture heights is 13.16%, indicating high accuracy. When the closure pressure is below 50 MPa, the theoretical and experimental results exhibit excellent consistency with minimal deviation, with an error of 5.53%.

**Fig 22. Comparison between theoretical and experimental results different** **proppant diameters of "T" type**

From Fig 22, the root mean square error for different proppant diameters is 11.27%, which meets engineering accuracy requirements. Under low closure pressure conditions, the root mean square error is 3.96%, demonstrating high precision.

**Fig 23. Comparison between theoretical and experimental results under different** **fracture structures**

As shown in Figure 23, under identical conditions but different fracture network structures, the theoretical model results exhibit strong agreement with the experimental data. The root mean square error is 9.81%, with the primary source of error attributed to lower measurement accuracy under high closure pressure (60 MPa). At low closure pressures, the root mean square error decreases to 8.20%.

**Changes:** Please see lines 523-542 on pages 25 and 26 marked with yellow highlighting.

**Comment #6:***. The model exhibits significant errors (13%) under high closure pressure, yet the error sources are not thoroughly analyzed. Add discussion on error origins..*

**Response:** The authors thank the reviewer for comment. The accuracy decreases under high closure pressures, primarily due to limitations in the precision and reliability of experimental measurements under such conditions. Additionally, the new model assumes that all capillary flow pathways are horizontally oriented, whereas in reality, both horizontal and vertical capillaries exist within the proppant. At low pressure, gas predominantly flows along horizontal directions with minimal vertical movement. Under high closure pressures, proppant crushing increases gas flow through vertical capillaries, causing the model to underestimate experimental results and resulting in larger deviations.

**Changes:** Please see lines 543-554 on pages 26 marked with yellow highlighting.

**Comment #7:***. The critical F-value criteria in Table 4 are undefined. Specify the statistical testing basis.*

**Response:** The authors thank the reviewer for comment. The F critical value is selected according to the F value critical value table with a confidence interval of 0.01.

**Changes:** Please see lines 321-322 on pages 15 marked with yellow highlighting and Table 4.

**Comment #8:***. The conclusion lacks clarity on how the findings guide field fracturing optimization (e.g., optimal proppant size selection, fracture network design). Include engineering application recommendations.*

**Response:** The authors thank the reviewer for comment. We added one conclusion: For fracture network fracturing optimization, it is recommended to use smaller proppant particle sizes (40-70 mesh) with lower proppant concentration and high-strength proppant. In fracture network design, an aspect ratio of 4~5 is preferred, and multiple secondary fractures should be designed near the wellbore to enhance conductivity.

**Changes:** Please see lines 602-606 on pages 28 marked with yellow highlighting.

**Comment #9:***. Author abbreviations in some references (e.g., Refs. 23, 27) are inconsistent. Standardize formatting.*

**Response:** The authors thank the reviewer for carefully reading. We have revised the format and corrected the errors in the references according to the requirements of journal references.

**Changes:** Please see lines 672-674, lines 682-684 on pages 31 marked with yellow highlighting.

**Comment #10:***. Some sentences contain grammatical errors (e.g., "Due the complex structure" should be "Due to..."). Perform full-text proofreading.*

**Response:** The authors thank the reviewer for reminder. We have corrected the errors. We have double-checked the manuscript and corrected the grammar errors in the revised manuscript. We also have revised our manuscript thoroughly, and the language is refined to make the manuscript more concise and logical.

**Changes:** Please see the revised manuscript and lines 225-226 on pages 10 marked with yellow highlighting.

Sincerely

Jinjin Gao
