# Supplementary material for: Experimental and theoretical investigation on the conductivity of complex fracture network in unconventional gas reservoirs
Source: PLoS One. 2025 Jun 13;20(6):e0325825. doi: 10.1371/journal.pone.0325825 (PMC12165408; doi:10.1371/journal.pone.0325825)
Supplement: Attachment — Submitted filename: Response to Reviewers and Editor - R2.docx [file pone.0325825.s005.docx]

**Response to Reviewers and Editor**

Journal:	**PLOS ONE**

Manuscript ID:	**PONE-D-25-07188R1**

Paper Title:	**Experimental and theoretical investigation on the conductivity of complex fracture network in unconventional gas reservoirs**

Authors:	**Jinjian Gao, Lanxiao Hu**

Dear Editor,

Thank you for guiding us through the final stage of manuscript preparation. We sincerely appreciate your emphasis on rigorous language and presentation standards.

**Academic Editor**

**Comment:** *This manuscript comes to the last step. Please fully check both English and presentations. A partially marked version is attached for your reference..*

**Response:** The authors thank the academic editor for carefully reading. Dr. Hu, the second author, who has four years of academic experience in Canada, was invited to check and revise the English language. The manuscript has undergone comprehensive formatting adjustments aligning with the journal's style specifications. We have double-checked the manuscript and corrected the grammar errors in the revised manuscript. We also have revised our manuscript thoroughly, and the language is refined to make the manuscript more concise and logical. The linguistic expressions have been refined into more academically rigorous English formulations

**Changes:** Please see the revised manuscript.

We would be honoured to implement any additional specific adjustments per your expert guidance. Thank you for your invaluable support in advancing this work.

Sincerely

Jinjin Gao

Corresponding Author
